# Transcription activator-like effectors from endosymbiotic bacteria control the reproduction of their fungal host

**DOI:** 10.1128/mbio.01824-23

**Published:** 2023-11-16

**Authors:** Ingrid Richter, Zerrin Uzum, Philipp Wein, Evelyn M. Molloy, Nadine Moebius, Timothy P. Stinear, Sacha J. Pidot, Christian Hertweck

**Affiliations:** 1 Department of Biomolecular Chemistry, Leibniz Institute for Natural Product Research and Infection Biology, Jena, Germany; 2 Department of Microbiology and Immunology, Doherty Institute, Melbourne, Australia; 3 Institute of Microbiology, Faculty of Biological Sciences, Friedrich Schiller University Jena, Jena, Germany; 4 Cluster of Excellence Balance of the Microverse, Friedrich Schiller University Jena, Jena, Germany; Karlsruhe Institute of Technology, Karlsruhe, Germany

**Keywords:** host control, *Mycetohabitans*, *Rhizopus microsporus*, sporulation, symbiosis

## Abstract

The phytopathogenic fungus *Rhizopus microsporus* harbors a bacterial endosymbiont (*Mycetohabitans rhizoxinica*) that produces a crucial virulence factor responsible for the characteristic symptoms of rice seedling blight. The persistence of this unique microbial alliance is ensured by evasion of fungal defense mechanisms by the resident endosymbionts and their strict control of fungal reproduction. A functional bacterial Type 3 secretion system (T3SS) is essential for the establishment of the symbiosis. Yet, the nature of the effector(s) secreted through the T3SS, and their effects on fungal physiology, has remained elusive. Using bioinformatic analysis, we identified genes encoding potential T3SS effectors, namely an alanine-tryptophan-arginine triad (AWR) peptide and a set of transcription activator-like (TAL) effectors, in the genomes of eight endofungal *Mycetohabitans* strains. Co-culture experiments demonstrated that the sporulating phenotype of apo-symbiotic *R. microsporus* is unaffected upon reinfection with *M. rhizoxinica* Δ*awr* compared to with wild-type *M. rhizoxinica*. In contrast, the ability of apo-symbiotic *R. microsporus* to produce mature sporangiophores when reinfected with *M. rhizoxinica* strains deficient in individual MTALs (*M. rhizoxinica* Δ*mtal1*, *M. rhizoxinica* Δ*mtal2*, and *M. rhizoxinica* Δ*mtal3*) is significantly reduced. *Trans*-complementation experiments showed restoration of fungal sporulation, thus confirming that TAL effectors produced by *M. rhizoxinica* (MTAL1, MTAL2, and MTAL3) are needed for fungal sporulation. Using fluorescence microscopy, we show that AWR- and MTAL-deficient *M. rhizoxinica* strains successfully colonize apo-symbiotic *R. microsporus*, revealing the importance of bacterial MTALs in establishing a stable symbiosis after fungal colonization. Our findings attribute a new function to members of the MTAL family of T3SS-associated effectors and provide deeper insights into host control by prokaryotic symbionts.

**IMPORTANCE:**

Interactions between fungi and bacteria are critically important in ecology, medicine, and biotechnology. In this study, we shed light on factors that promote the persistence of a toxin-producing, phytopathogenic *Rhizopus-Mycetohabitans* symbiosis that causes severe crop losses in Asia. We present an unprecedented case where bacterially produced transcription activator-like (TAL) effectors are key to maintaining a stable endosymbiosis. In their absence, fungal sporulation is abrogated, leading to collapse of the phytopathogenic alliance. The *Mycetohabitans* TAL (MTAL)-mediated mechanism of host control illustrates a unique role of bacterial effector molecules that has broader implications, potentially serving as a model to understand how prokaryotic symbionts interact with their eukaryotic hosts.

## INTRODUCTION

Bacteria that live in close association with eukaryotic hosts may control and exploit their host via pathogenic or mutualistic interactions (1). To take control of their eukaryotic hosts, bacteria have evolved dedicated secretion systems (Types 1 to 9) that secrete a wide range of effectors into the extracellular space or directly into the target cell (2–4). These sophisticated molecules are central to some of the most devastating diseases (5). For example, the human and animal pathogen *Burkholderia mallei*, the causative agent of the highly lethal disease glanders, depends on Type 3 secretion system (T3SS) effectors to invade the cytoplasm of its host (6). Plant-pathogenic *Xanthomonas* and *Ralstonia* species colonize their hosts with the help of T3SS-associated transcription activator-like (TAL) effectors that imitate plant transcription factors (7–9). The fungal pathogen *Janthinobacterium agaricidamnosum* secretes degradative enzymes and putative effector proteins via the Type 2 secretion system (T2SS) and T3SS. These effectors aid bacterial invasion of the white button mushroom, leading to rapid tissue decay and soft rot disease (10).

**Fig 1:**
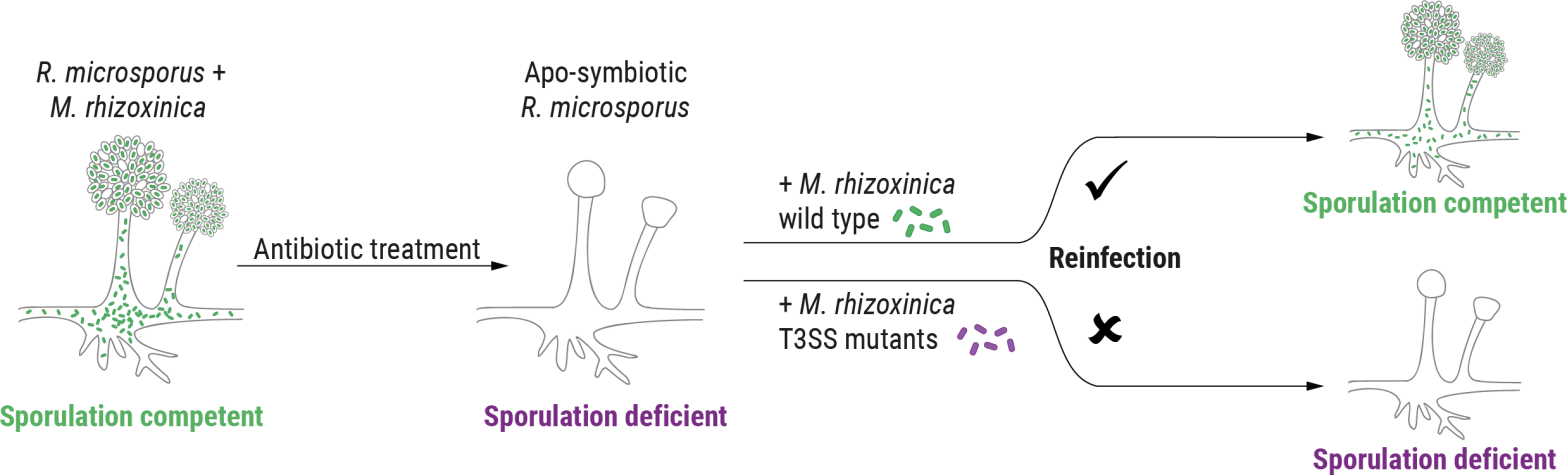
Schematic representation of *M. rhizoxinica-*dependent sporulation of *R. microsporus*. Mature sporangia are absent in fungus cured of endosymbionts by antibiotic treatment. Co-cultivation of wild-type *M. rhizoxinica* with the apo-symbiotic fungus leads to reinfection, and the host’s ability to form sporangiophores is restored. T3SS mutants fail to reinfect hyphae, causing a lack of sporulation.

The effectors and associated secretion systems utilized by pathogenic bacteria to control their hosts have been disproportionately studied compared to those employed by symbiotic bacteria, which remain underexplored. The endosymbiosis between the Mucoromycota fungus *Rhizopus microsporus* and the bacterium *Mycetohabitans rhizoxinica* (formerly *Burkholderia rhizoxinica*) is an intriguing example of host control by a symbiont (11–13). Fungal reproduction through spores relies exclusively on the presence of endobacteria (Fig. 1). When *R. microsporus* is cured of its endosymbiont, the fungal host is unable to reproduce vegetatively (13). This strict control of sporulation ensures that the endosymbiosis persists over generations because the endosymbionts are translocated into the fungal spores during host reproduction (13). Maintaining its endosymbiont has clear advantages for the fungus, such as killing rice seedlings with the bacterial secondary metabolite rhizoxin (14) and protection from micropredators (15).

Effectors have been repeatedly associated with the establishment and maintenance of the *R. microsporus–M. rhizoxinica* symbiosis. For example, *M. rhizoxinica* invades the fungal host by secreting effector proteins via the T2SS (16), while a functional T3SS is required for the formation of a stable symbiosis (17). Gene expression analyses further hinted at the importance of effectors secreted via the T3SS since nearly 60 T3SS-associated effector genes are over-expressed during establishment of the symbiosis (18). However, *Mycetohabitans* TAL (MTAL1) is the only effector that has so far been recognized as essential for the maintenance of the *R. microsporus–M. rhizoxinica* symbiosis (19). Using a combination of microfluidics and fluorescence microscopy, we showed that MTAL1 is crucial for evading host immune response and thus facilitates the intracellular survival of *M. rhizoxinica* (19). While these findings indicate that effectors secreted through the T3SS are important symbiosis factors, there is a lack of knowledge about whether they could be involved in controlling fungal reproduction and development.

Here, we show that homologous genes encoding T3SS-associated effectors, namely alanine-tryptophan-arginine triad (AWR) peptides and *Mycetohabitans* TALs (MTAL1, MTAL2, and MTAL3), are widespread in the genomes of endofungal *Mycetohabitans* symbionts. Furthermore, we demonstrate that MTALs are instrumental in controlling fungal reproduction in the phytopathogenic *R. microsporus–M. rhizoxinica* bacterial*–*fungal alliance.

## RESULTS

### AWR peptides are universally conserved in endofungal *Mycetohabitans* symbionts

**Fig 2:**
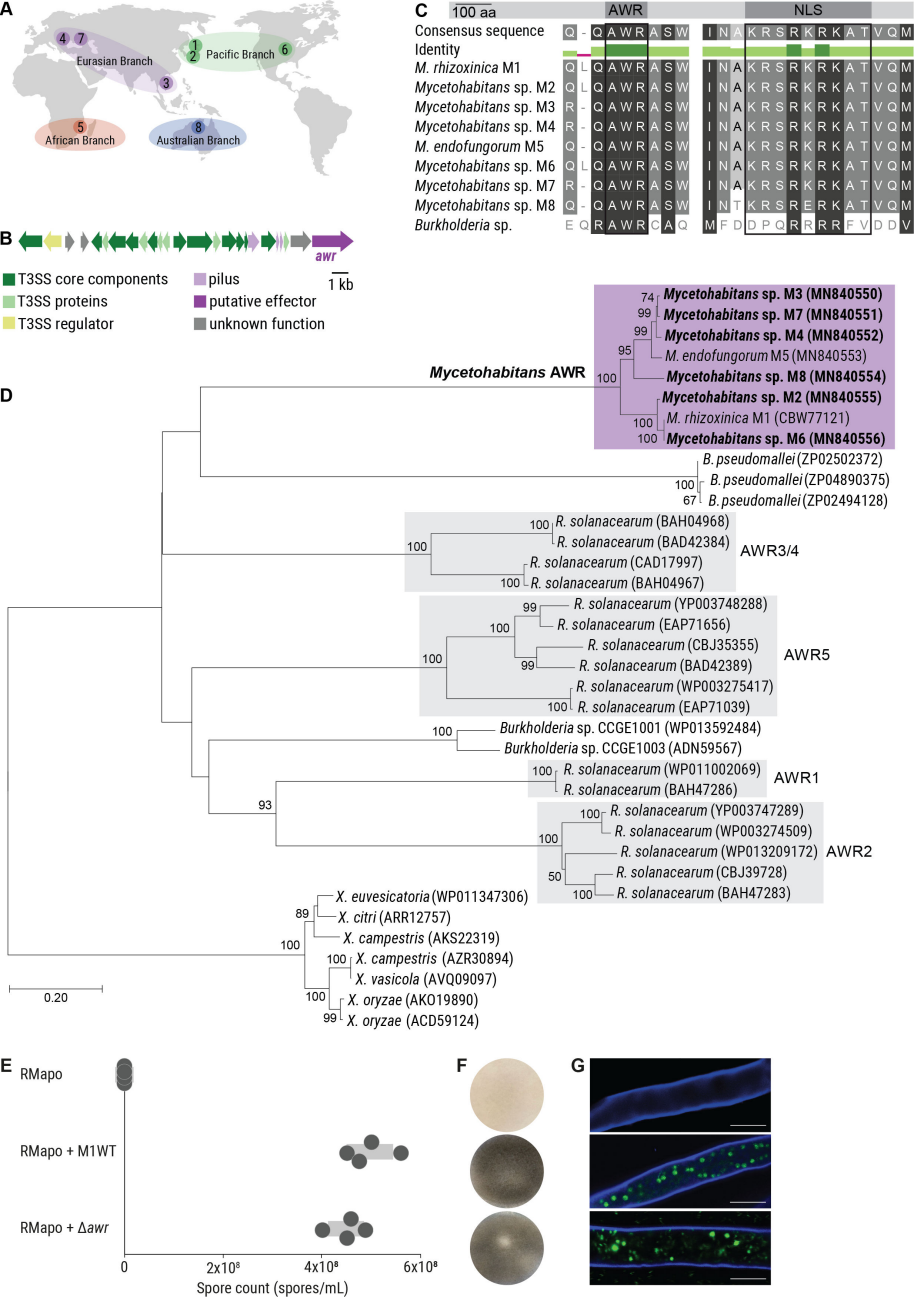
Identification of an AWR effector associated with the T3SS from endofungal *Mycetohabitans* species. (A) Map depicting global distribution of *R. microsporus* strains containing *Mycetohabitans* symbionts and their classification into four geographical branches. (B) Schematic representation of the T3SS gene cluster (*sct*) located on plasmid pBR01 that encodes the Type 3 secretion apparatus. The putative Type 3 effector-encoding gene (*awr*) is indicated in dark purple. (C) Multiple sequence alignment of the AWR peptides from endofungal *Mycetohabitans* strains, and a related *Burkholderia* species (CCGE1003). The functional domains of the protein (nuclear localization sequence, NLS) are labeled. (D) Phylogenetic tree of AWR homologs from plant pathogenic *Ralstonia solanacearum* and plant-associated *Burkholderia* species. The phylogenetic analyses were performed using MEGA7 (see Materials and Methods for details). Sequences newly identified in this study are highlighted in bold (see Table S3). GenBank accession numbers are given in brackets. (E) Spore count of apo-symbiotic *R. microsporus* incubated with solvent control (RMapo) or reinfected with *M. rhizoxinica* wild type (RMapo + M1WT), or AWR-deficient *M. rhizoxinica* (RMapo + Δ*awr*). Dots represent four independent replicates (*n* = 4 biological replicates) and grey bars mark ± one standard error of the mean. (F) Representative photographs of fungal cultures depicting the formation of sporangiophores (black mycelium). Apo-symbiotic *R. microsporus* incubated with solvent control does not sporulate (white mycelium). Strain order as given in panel E. (G) Localization of bacteria (green) inside the fungal hyphae (blue) was confirmed by fluorescence microscopy. Strain order as given in panel E. Scale bars: 10 µm.

Initial physical contact of *M. rhizoxinica* with *R. microsporus* induces the expression of approximately 60 T3SS-associated effector genes in *M. rhizoxinica* (18), hinting that the cognate effectors act as mediators of this bacterial*–*fungal interaction. Therefore, we sought to identify genes coding for putative Type 3 effector proteins by searching publicly available genomes of eight *Mycetohabitans* species that are endosymbionts of globally distributed *R. microsporus* strains (see Table S1). The fungal strains and their corresponding endosymbionts were isolated from varying habitats (e.g., arid and forest soils, food products, and human tissue) and are grouped into the following four, geographically distant branches: (i) Pacific branch containing *M. rhizoxinica* HKI-454 (M1), *Mycetohabitans* sp. HKI-512 (M2), and *Mycetohabitans* sp. HKI-513 (M6); (ii) Eurasian branch containing *Mycetohabitans* sp. HKI-455 (M3), *Mycetohabitans* sp. HKI-402 (M4), and *Mycetohabitans* sp. HKI-403 (M7); (iii) African branch containing *Mycetohabitans endofungorum* HKI-456 (M5); and (iv) Australian branch containing *Mycetohabitans* sp. HKI-404 (M8) (Fig. 2A; see also Table S1) (20).

Using T3SS prediction tools (see Materials and Methods for details) (21, 22), we found that each *Mycetohabitans* genome sequence harbors a single gene encoding an AWR peptide (Fig. 2B and C), a potential T3SS effector. Homologs belonging to the *awr* gene family, present in the genomes of a number of bacterial pathogens including phytopathogenic *Burkholderia* and *Ralstonia* strains, collectively contribute to bacterial virulence (23). For example, deletion of all five *awr* genes in *Ralstonia solanacearum* severely impairs its capacity to multiply in its host plant (23, 24).

To confirm the identified sequences as genuine *awr* orthologs, a phylogeny was inferred from alignment of the predicted endofungal *Mycetohabitans* AWR peptide sequences. These sequences were aligned with homologous AWR sequences from Gram-negative plant and animal pathogens as well as a number of *Burkholderia* symbionts (Fig. S1) (23). The tree was rooted in the *Xanthomonas* (γ-proteobacteria) sequences, which are more distantly related to the *Ralstonia* and *Burkholderia* (β-proteobacteria) sequences. According to the inferred phylogeny, *Mycetohabitans* AWR peptides form a monophyletic group with the nearest relative being a protein of unknown function found in the mammalian pathogen *Burkholderia pseudomallei* (GenBank accession number: ZP02502372) (25). In addition, all eight endofungal *Mycetohabitans* AWR peptides possess sequences at their C termini that are consistent with the previously described monopartite nuclear localization sequence (NLS), K(K/R)X(K/R) (Fig. 2C; see also Table S2) (26).

### AWR peptide plays no discernible role in *R. microsporus* sporulation

The association between *R. microsporus* ATCC62417 and *M. rhizoxinica* was used as a model system to probe whether the bacterial AWR plays a role in this bacterial–fungal symbiosis. To this end, we performed a targeted gene deletion of the *M. rhizoxinica awr* gene using a double-crossover strategy ( Fig. S2A and B) (17). Although *M. rhizoxinica* can be maintained in axenic cultures under laboratory conditions, genetic manipulations are challenging. The long doubling time and aggregation of cells (27) significantly impair the selection process. Despite these hurdles, we succeeded in deleting the *awr* gene in *M. rhizoxinica* to generate *M. rhizoxinica* Δ*awr*::Kan^R^ (*M. rhizoxinica* Δ*awr*; Fig. S2C) by continuous passaging of transformants on double-selection media. The AWR-deficient strain was examined for its ability to promote fungal reproduction using a previously described sporulation bioassay (17). First, *R. microsporus* is treated with antibiotics to eliminate its natural *M. rhizoxinica* endosymbionts (13) resulting in an apo-symbiotic (endosymbiont-free) fungal strain that is unable to sporulate (Fig. 1). Apo-symbiotic *R. microsporus* is then individually co-cultured with the *M. rhizoxinica* wild type and the *M. rhizoxinica* mutants of interest. An uninoculated medium control is also included. If spore formation occurs after 4–7 days, it indicates successful establishment of the symbiosis (13). Subsequently, sporulation efficiency can be calculated by comparing the sporulating phenotype conferred by the *M. rhizoxinica* mutants to that conferred by the *M. rhizoxinica* wild type.

When apo-symbiotic *R. microsporus* and wild-type *M. rhizoxinica* are co-cultured, mature sporangiophores can be seen after 4 days. Similar numbers of spores are observed upon co-cultivation with *M. rhizoxinica* Δ*awr*, i.e., the wild-type phenotype is restored (Fig. 2E and F). In addition, fungal mycelium reinfected with *M. rhizoxinica* Δ*awr* did not show any growth deficiencies and is characterized by a similar macroscopic phenotype as the wild type (i.e., fluffy mycelium with aerial hyphae) (Fig. 2F). Fluorescence microscopy was used to monitor whether bacteria (stained with SYTO9) are able to recolonize fungal hyphae (stained with calcofluor white). The level of intracellularly located *M. rhizoxinica* Δ*awr* is comparable to that of wild-type *M. rhizoxinica* (Fig. 2G), suggesting that AWR is not essential for *M. rhizoxinica* to enter *R. microsporus* hyphae and to establish a stable symbiosis. Indeed, we observed no septa formation in *R. microsporus* reinfected with *M. rhizoxinica* Δ*awr* (Fig. 2G), as expected for a stable endosymbiosis (19). These results suggest that AWR is not essential for *M. rhizoxinica* to enter *R. microsporus* hyphae and to establish a stable symbiosis. However, given that *awr* genes are universally conserved in the genomes of endofungal *Mycetohabitans* spp. despite their highly reduced genomes (22, 28), AWR peptides may still play as-yet-unknown physiological roles.

### Genes encoding TALs are widespread in endofungal *Mycetohabitans* symbionts

**Fig 3:**
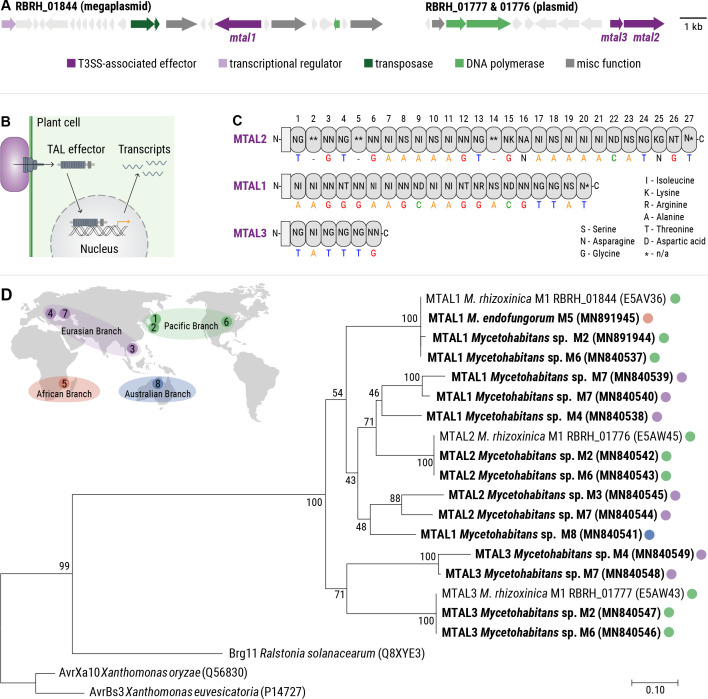
Identification of predicted MTAL effectors from endofungal *Mycetohabitans* species. (A) Schematic illustration of the gene clusters encoding MTAL1 (RBRH_01844), MTAL2 (RBRH_01776), and MTAL3 (RBRH_01777) indicated in dark purple. (B) Mode of action of TALs from *Xanthomonas* sp. TALs are secreted directly into plant cells via the T3SS, translocate to the nucleus, and induce expression of target genes. (C) Schematic representation of the overall domain structure of *M. rhizoxinica* MTALs and the amino acid tandem repeats (top) that specify the target nucleotide sequence (bottom). Cryptic repeats that have less than 45% amino acid identity with core repeats are marked with an asterisk (*, n/a: not annotated). Modified from Lange et al. (30). (D) Phylogenetic tree of TAL proteins from eight endofungal *Mycetohabitans* species, and plant pathogenic *R. solanacearum* and *Xanthomonas* sp. MTAL sequences identified in this study are highlighted in bold and GenBank accession numbers are given in brackets (Table S3). The distribution of MTALs across the four *Mycetohabitans* branches is indicated as follows: green, Pacific branch; purple, Eurasian branch; orange, African branch; and blue, Australian branch.

Since AWR deficiency had no discernible effect on fungal reproduction, we continued our search for putative Type 3 effectors in the genomes of the eight endofungal *Mycetohabitans* species (M1–M8). Genes encoding three putative T3SS effectors, with the locus tags RBRH_01844 (2,316bp), RBRH_01776 (2,994bp), and RBRH_01777 (936bp), were previously identified in the genome of *M. rhizoxinica* (Fig. 3A) (29–31). Since these genes are highly similar to those encoding TAL effectors, they were named MTAL (for *Mycetohabitans* transcription activator-like effectors; MTAL1: RBRH_01844; MTAL2: RBRH_01776; and MTAL3: RBRH_01777). TAL effectors are employed by bacterial pathogens such as phytopathogenic *Burkholderia*, *Ralstonia*, and *Xanthomonas* strains to modify the expression of host plant genes, which aids bacterial colonization and virulence (Fig. 3B) (29, 32, 33).

To investigate whether MTALs are a common feature in endofungal *Mycetohabitans* strains, we surveyed the publicly available genomes for *mtal* homologs. Using bioinformatic analysis, we discovered that endofungal *Mycetohabitans* strains commonly encode multiple *mtal* genes in their genomes (Table S3). Specifically, *mtal1* was detected in the genomes of *Mycetohabitans* M1, M4, M6, M7, and M8, *mtal2* in *Mycetohabitans* M1, M2, M3, M6, and M7, and *mtal3* in *Mycetohabitans* M1, M2, M4, M6, and M7. The highly repetitive nature of *mtal* genes hinders prediction within the genome of a given organism. We attempted to amplify hidden *mtal* genes via polymerase chain reaction (PCR) (Table S4) but only obtained *mtal1* sequences from *Mycetohabitans* strains M2 and M5. Details of all *mtal* genes, whether identified bioinformatically or by PCR amplification and Sanger sequencing, were deposited in GenBank under the accession numbers listed in Table S3.

All members of the TAL effector family, including *Xanthomonas* and *Ralstonia* TALs, are characterized by a central protein domain consisting of tandem-arranged repeats. These repeats mediate sequence-specific binding to host DNA, which alters gene expression to benefit the pathogen (Fig. 3C) (34). In addition, *Xanthomonas* and *Ralstonia* TAL proteins possess a Type 3 secretion signal, a nuclear localization sequence (NLS), and an activation domain, all of which are required for the transcriptional activation of host plant genes (32). As the short C termini of MTAL proteins do not contain NLSs, we searched for NLS motifs within the entire MTAL protein sequences using the NucPred prediction software (see Materials and Methods for details). Some MTAL1 and MTAL2 proteins are predicted to localize to the nucleus (52%), while the remainder localizes to the cytoplasm (48%). Localization of MTAL3 could not be predicted (Table S5). In addition, we identified the NLS-like sequence “RIRK” in the C termini of all MTAL1 and MTAL2 proteins (Table S5). This NLS-like sequence was previously shown to mediate translocation of MTAL2 from *Mycetohabitans* sp. B13 (M4) to the nucleus of *Saccharomyces cerevisiae* (35).

Phylogenetic analysis of predicted MTAL proteins was performed using the central protein domain consisting of tandem-arranged repeats (Fig. 3C) (34). The inferred phylogeny confirmed *mtal* sequences as encoding TAL orthologs, with *R. solanacearum* TALs being the closest relatives (Fig. 3D; see also Fig. S3). In addition, detection of *mtal1*, *mtal2*, and *mtal3* in the genomes of *Mycetohabitans* species belonging to four geographically distant branches (Fig. 3D) (20) reveals a wide distribution of *mtal* genes in fungal endosymbionts, thus adding to the number of previously identified MTALs in *Mycetohabitans* strains (35). Based on the prevalence of *mtal* genes in the genomes of endofungal *Mycetohabitans* and their transcriptional upregulation during physical contact of *M. rhizoxinica* and *R. microsporus* (18), we deemed MTALs potentially important effectors in the *Rhizopus-Mycetohabitans* symbiosis.

### Sporulation of *R. microsporus* containing MTAL-deficient *M. rhizoxinica* is greatly diminished

In order to investigate whether MTALs contribute to the *Rhizopus-Mycetohabitans* symbiosis, we performed targeted gene deletions using a double-crossover strategy (Fig. S2A and B) (17). The *mtal* genes were individually deleted in *M. rhizoxinica* to generate *M. rhizoxinica* Δ*mtal2*::Kan^R^ (*M. rhizoxinica* Δ*mtal2*) and *M. rhizoxinica* Δ*mtal3*::Kan^R^ (*M. rhizoxinica* Δ*mtal3*; Fig. S2D and E). The *mtal1* gene was previously deleted in *M. rhizoxinica*, yielding *M. rhizoxinica* Δ*mtal1*::Apra^R^ (*M. rhizoxinica* Δ*mtal1*) (19).

**Fig 4:**
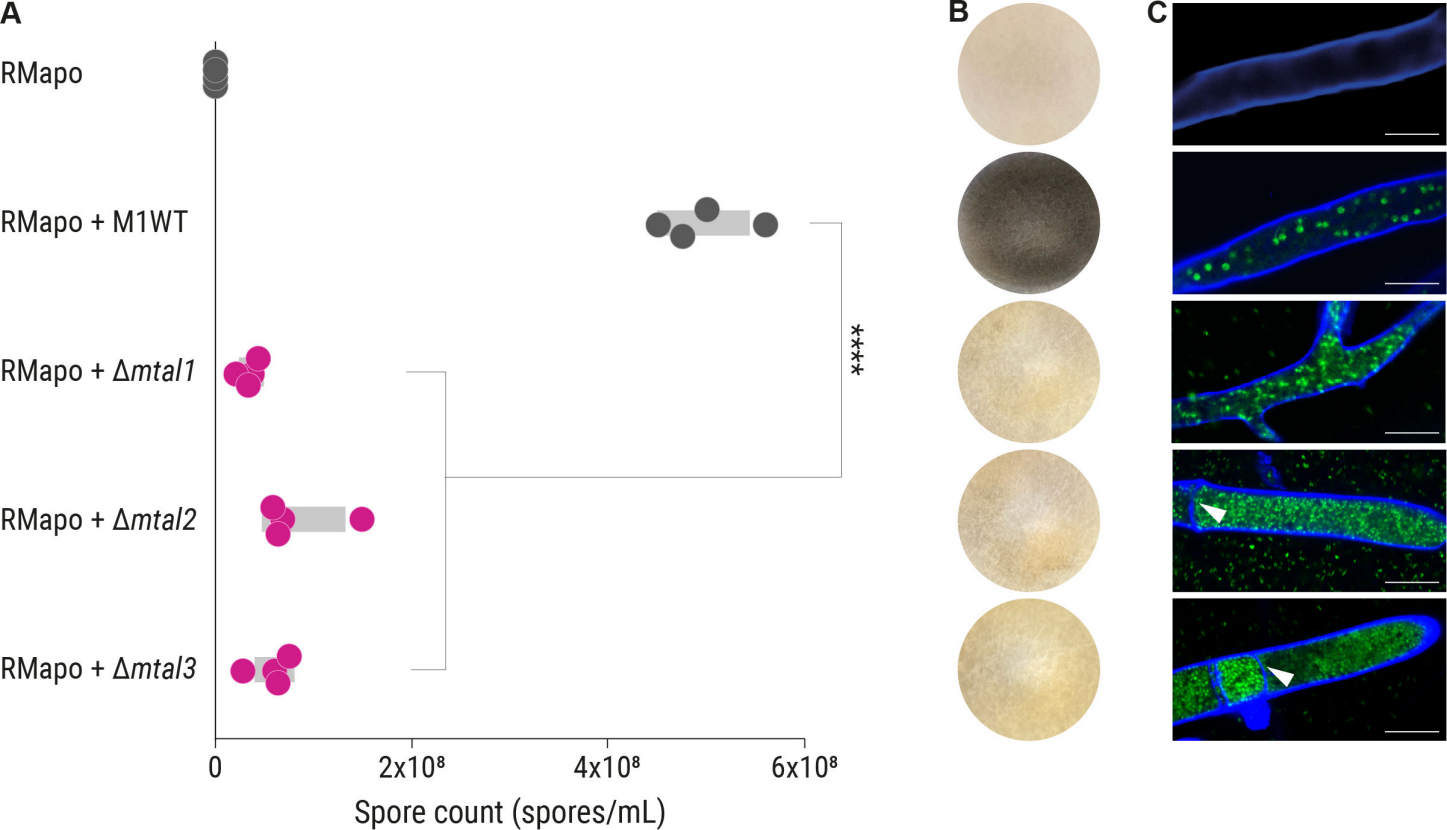
Sporulation ability of *R. microsporus* containing MTAL-deficient *M. rhizoxinica* is reduced. (A) Spore count of apo-symbiotic *R. microsporus* incubated with solvent control (RMapo) or reinfected with *M. rhizoxinica* wild type (RMapo + M1WT) or MTAL-deficient *M. rhizoxinica* (RMapo + Δ*mtal1*, RMapo + Δ*mtal2*, and RMapo + Δ*mtal3*). Dots represent four independent replicates (*n* = 4 biological replicates) and grey bars mark ± one standard error of the mean. One-way analysis of variance with Tukey’s multiple comparison test (****P < 0.0001; Table S6). (B) Representative photographs of fungal cultures. Apo-symbiotic *R. microsporus* reinfected with *M. rhizoxinica* wild type sporulates after 4 days of incubation (black mycelium). The apo-symbiotic fungus shows no sporulation. Strain order as given in panel A. (C) Localization of bacteria (green) inside the fungal hyphae (blue) was confirmed by fluorescence microscopy. Strain order as given in panel A. Scale bars: 10 µm. White arrowheads point to septa. Data points, sporulation image, and fluorescence microscopic image of RMapo and RMapo + M1WT are the same as depicted in Fig. 2E through G, respectively.

We probed whether MTAL-deficient *M. rhizoxinica* strains have an effect on host reproduction using the sporulation bioassay (17). Apo-symbiotic *R. microsporus* does not produce sporangiophores (Fig. 4A and B). When wild-type *M. rhizoxinica* and apo-symbiotic *R. microsporus* are co-cultured, sporulation can be seen after 4 days indicating the successful establishment of the symbiosis (Fig. 4A and B). In contrast, in the case of co-cultivation with *M. rhizoxinica* Δ*mtal1*, *M. rhizoxinica* Δ*mtal2*, and *M. rhizoxinica* Δ*mtal3*, only a limited number of mature sporangia are formed (Fig. 4A and B). Indeed, the sporulation efficiency is significantly reduced (*P* < 0.001) when compared to wild-type *M. rhizoxinica* and *M. rhizoxinica* Δ*awr* (Fig. 4A; see also Table S6).

We considered the possibility that a lack of sporulation could be due to the inability of the MTAL-deficient *M. rhizoxinica* to recolonize the fungal host, as was reported for strains lacking a functional T2SS and T3SS (16, 17). We therefore used fluorescence microscopy to monitor bacteria (stained with SYTO9) inside the fungal hyphae (stained with calcofluor white) following reinfection. We noted that *M. rhizoxinica* Δ*mtal1*, *M. rhizoxinica* Δ*mtal2*, and *M. rhizoxinica* Δ*mtal3* successfully recolonize apo-symbiotic *R. microsporus* (Fig. 4C). In addition, MTAL-deficient *M. rhizoxinica* reaches higher cell densities within the host cytosol compared to wild-type *M. rhizoxinica* and *M. rhizoxinica* Δ*awr* (Fig. 2G and 4C). In addition, we observed formation of septa in hyphae containing *M. rhizoxinica* Δ*mtal2* and *M. rhizoxinica* Δ*mtal3*, as was reported for *R. microsporus* hyphae containing *M. rhizoxinica* Δ*mtal1* (19).

In order to confirm that the inability of the MTAL-deficient *M. rhizoxinica* strains to restore fungal sporulation is solely due to disruption of the *mtal* genes, we performed an *in vivo trans*-complementation experiment. We constructed the expression vectors, pBBR_P*_s12_*_*mtal2* and pBBR_P*_s12_*_*mtal3*, in which the *mtal* gene is under the control of a constitutive promoter. We transformed *M. rhizoxinica* Δ*mtal2* and *M. rhizoxinica* Δ*mtal3* with pBBR_P*_s12_*_*mtal2* and pBBR_P*_s12_*_*mtal3*, respectively (19), yielding *M. rhizoxinica* Δ*mtal2* pBBR-*mtal2* and *M. rhizoxinica* Δ*mtal3* pBBR-*mtal3* (complemented *M. rhizoxinica mtal* mutants; Fig. S2F). *M. rhizoxinica* Δ*mtal2* and *M. rhizoxinica* Δ*mtal3* were also transformed with the empty vector pBBR_P*_s12_* to generate the control strains *M. rhizoxinica* Δ*mtal2* pBBR∅ and *M. rhizoxinica* Δ*mtal3* pBBR∅ (empty vector controls; Fig. S2F). *M. rhizoxinica* Δ*mtal1* pBBR-*mtal1* (complemented *M. rhizoxinica mtal1* mutant) and *M. rhizoxinica* Δ*mtal1* pBBR∅ (empty vector control) were generated previously (19).

**Fig 5:**
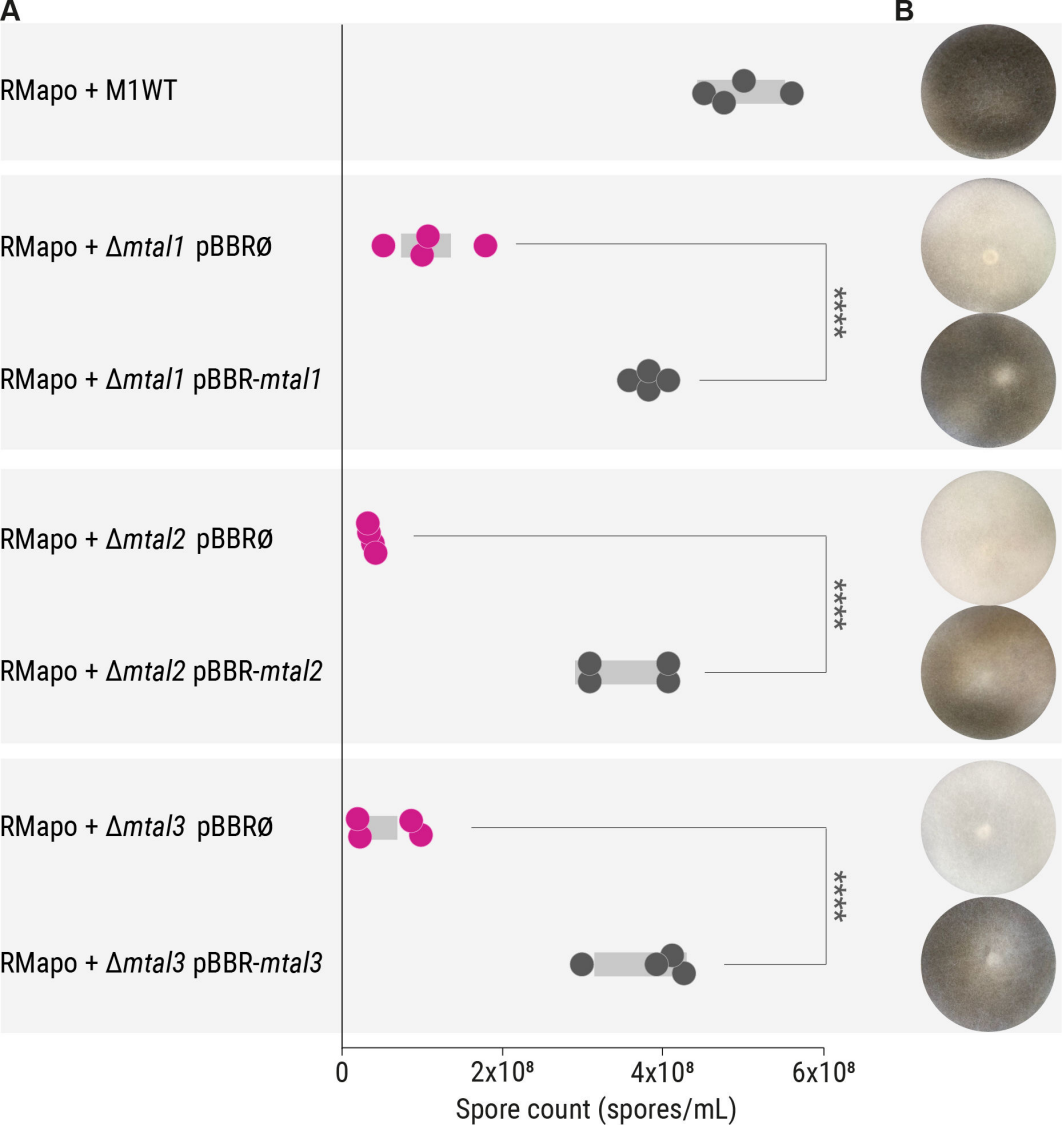
Sporulation ability of *R. microsporus* containing complemented *M. rhizoxinica mtal* mutants is restored. (A) Spore count of apo-symbiotic *R. microsporus* reinfected with *M. rhizoxinica* wild type (RMapo + M1WT), *M. rhizoxinica mtal* mutants containing the relevant empty vector (RMapo + *M. rhizoxinica* Δ*mtal1* pBBR∅, RMapo + *M. rhizoxinica* Δ*mtal2* pBBR∅, and RMapo + *M. rhizoxinica* Δ*mtal3* pBBR∅), or *M. rhizoxinica mtal* mutants constitutively expressing a plasmid-borne copy of the relevant *mtal* gene (RMapo *+ M. rhizoxinica* Δ*mtal1* pBBR-*mtal1*, RMapo + *M. rhizoxinica* Δ*mtal2* pBBR-*mtal2*, and RMapo + *M. rhizoxinica* Δ*mtal3* pBBR-*mtal3*). Dots represent four independent replicates (*n* = 4 biological replicates) and grey bars mark ± one standard error of the mean. One-way analysis of variance with Tukey’s multiple comparison test (****P < 0.0001; see Table S7). (B) Representative photographs of apo-symbiotic *R. microsporus* reinfected with *M. rhizoxinica* strains after 1 week of co-cultivation. Strain order as given in panel A. Data points and sporulation image of RMapo + M1WT are the same as depicted in Fig. 2E and F , respectively.

The ability of the complemented *M. rhizoxinica mtal* mutants to restore sporulation in the fungal host was assessed in the sporulation bioassay. Reinfection of apo-symbiotic *R. microsporus* with each of the three empty vector control strains (*M. rhizoxinica* Δ*mtal1* pBBR∅, *M. rhizoxinica* Δ*mtal2* pBBR∅, and *M. rhizoxinica* Δ*mtal3* pBBR∅) does not restore sporulation of the fungal host, whereas the complemented *M. rhizoxinica mtal* mutants (*M. rhizoxinica* Δ*mtal1* pBBR-*mtal1*, *M. rhizoxinica* Δ*mtal2* pBBR-*mtal2*, and *M. rhizoxinica* Δ*mtal3* pBBR-*mtal3*) readily trigger sporulation, in all cases restoring the wild-type phenotype (Fig. 5A and B). The sporulation efficiency is significantly increased in the complemented *M. rhizoxinica mtal* mutants (*P* < 0.001) when compared to the appropriate MTAL-deficient *M. rhizoxinica* strains (Fig. 5A; see also Table S7). The impaired ability of the MTAL-deficient strains to induce sporulation and the phenotypic complementation by *trans* expression of the relevant *mtaI* genes supports the proposal that T3SS-associated MTALs are essential effectors in the sporulation process of *R. microsporus*.

## DISCUSSION

**Fig 6:**
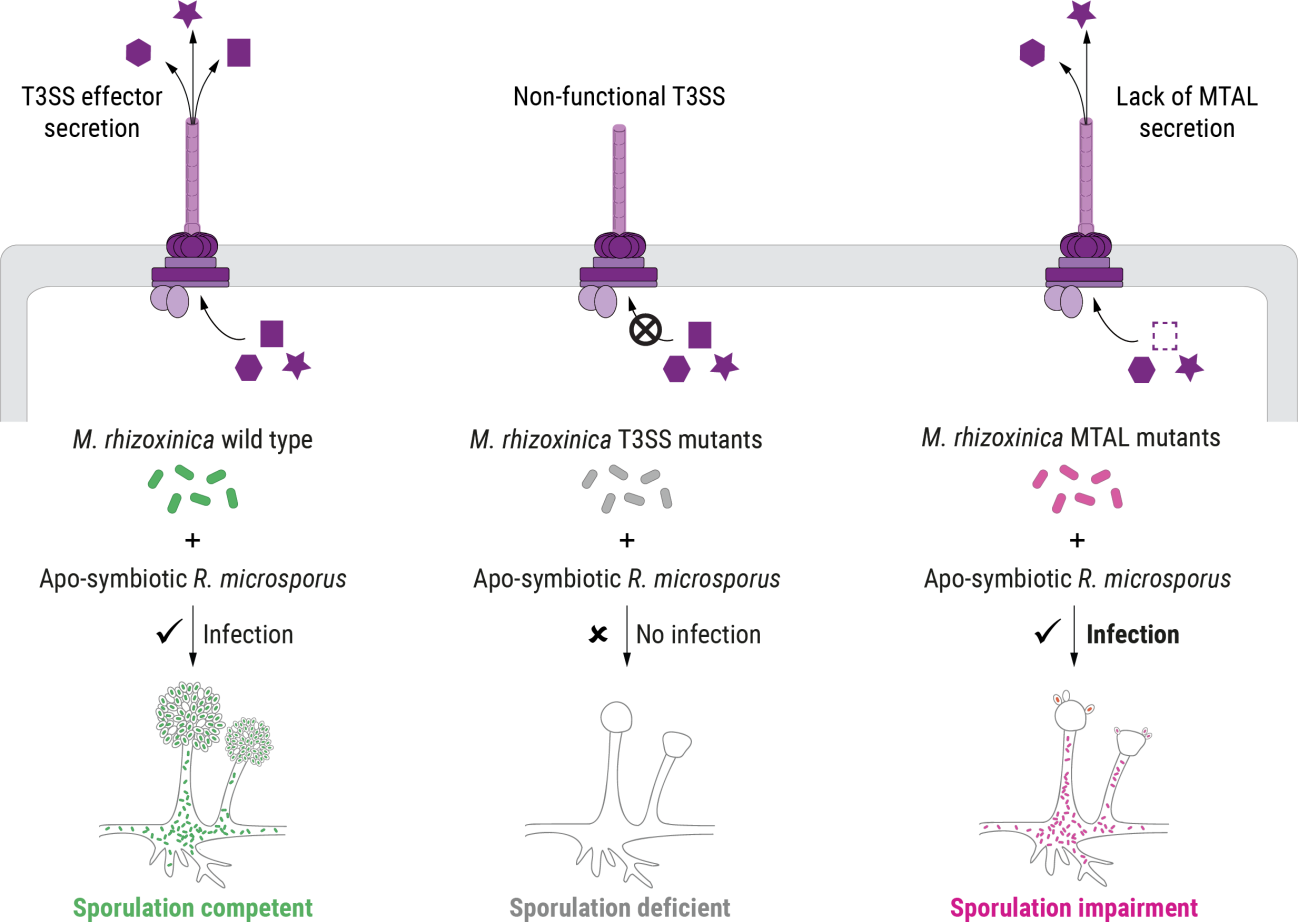
Schematic model of host control by endofungal bacteria. The recolonization of apo-symbiotic *R. microsporus* by the isolated wild-type endosymbiont, *M. rhizoxinica*, restores the host’s ability to form sporangiospores (left panel). *M. rhizoxinica* strains lacking a functional T3SS are unable to infect fungal hyphae and, as a consequence, fail to cause sporulation (middle panel). *M. rhizoxinica* that lack MTALs successfully reinfect the fungal host; however, in the absence of the T3SS-associated MTALs, the sporulating phenotype indicative of a stable symbiosis is profoundly impaired (right panel).

Bacteria that live in close association with eukaryotes can control their host by employing specialized effectors that are released through dedicated secretion systems (36). Among them, effector proteins delivered through the T3SS can be essential to host cell entry of Gram-negative bacterial pathogens, mutualists and, in rare cases, endosymbionts (17, 37, 38). TAL effectors are a prominent example of T3SS-associated proteins that allow plant-pathogenic bacteria to enter and control their hosts (8, 39, 40). Over the last two decades, TAL effectors have been engineered for application as genome editing tools due to their programmable DNA-binding properties (41). This has reinvigorated interest in TAL effectors leading to the discovery of TAL-related proteins in a wide range of bacterial species, including *M. rhizoxinica* (MTAL1, MTAL2, and MTAL3) (31). Here, we used a combination of genomic and functional studies to characterize T3SS-associated proteins in the fungal endosymbiont *M. rhizoxinica*. We found that fungal sporulation is independent of the T3SS-associated AWR protein, whereas MTAL proteins are instrumental in controlling host reproduction (Fig. 6).

By means of a sporulation bioassay, we show that each individual *M. rhizoxinica mtal* mutant (*M. rhizoxinica* Δ*mtal1*, *M. rhizoxinica* Δ*mtal2*, and *M. rhizoxinica* Δ*mtal3*) is unable to completely restore fungal sporulation, suggesting that MTALs are not functionally redundant in regulating sporulation. It is conceivable that distinct MTAL functions could arise from the varying number of the tandem-arranged repeats, which mediate sequence-specific DNA binding (34). In fact, MTAL proteins contain so-called core repeats (>45% identity to each other), which form the central section of the proteins with cryptic repeats (<45% identity to each other) at both the C-terminal and N-terminal ends (30). The number of these core repeats is highly variable between individual MTALs (*M. rhizoxinica* MTAL1: 20 repeats; *M. rhizoxinica* MTAL2: 27 repeats; and *M. rhizoxinica* MTAL3: 6 repeats; Fig. 2D) (30). Within each conserved repeat, only amino acids at positions 12 and 13 are highly variable, which are termed repeat variable di-residue (RVD). The RVD of each repeat determines the DNA base to which each repeat binds, referred to as the canonical TAL code (30, 39). The hypothesis that individual *M. rhizoxinica* MTAL proteins may have distinct functions in controlling host sporulation is supported by the two observations: (i) slight changes in the RVD sequence or in the number of repeats can change the DNA-binding specificity (30), and (ii) the RVD repeats of MTAL proteins are less conserved compared to *Xanthomonas* and *Ralstonia* TALs (29, 42). This functional diversity of individual MTALs is exemplified by MTAL2 from *Mycetohabitans* strain B13, consisting of 19 repeats, which is not involved in controlling fungal sporulation but instead contributes to the ability of *R. microsporus* to tolerate cell membrane stress (35). In addition, the reduced ability to tolerate membrane stress in *R. microsporus* containing MTAL2-deficient *Mycetohabitans* strain B13 could not be rescued by expressing MTAL2 from *Mycetohabitans* strain B14 (35), suggesting that MTAL proteins may target host genes with different functions.

In an effort to gain a glimpse at the possible means by which MTALs modulate host sporulation, we performed a bioinformatic search for possible target sequences in the *R. microsporus* genome. The analysis returned only partial matches for MTAL1 and MTAL2, and none for MTAL3. While the majority of matching sequences fall into the 5′ untranslated region of genes coding for DNA-associated proteins or within assembly gaps, the predicted MTAL1 target is located within the coding sequence of a membrane-bound transporter. MTAL2 was predicted to bind to DNA in the vicinity of genes coding for a histone binding protein (RBBP4), a fungal transcription activator, and a molecular chaperon protein (DnaJ). While these preliminary findings suggest that MTAL2 may control host sporulation through epigenetic regulation, experimental studies are needed to provide evidence of promoter binding.

We discovered a role for MTAL3 in controlling fungal reproduction. This is somewhat surprising as MTAL3 is predicted to contain only six tandem-arranged repeats and, consequently, is unable to bind DNA efficiently (30). However, it was previously reported that TAL effectors from various *Xanthomonas* strains can act through protein-protein interactions instead of binding to DNA (43, 44). For example, a class of structurally degenerate TAL effector-like proteins (TruncTALs) from rice pathogenic *X. oryzae* strains suppress certain plant disease resistance genes despite the inability to bind to DNA (43). In fact, some X. *oryzae* TAL effectors consisting of only 3.5 tandem-arranged repeats are able to mediate control of their host independent of a specific RVD sequence (45). Thus, it is conceivable that MTAL3 might function in a DNA-independent manner in the *Rhizopus-Mycetohabitans* symbiosis.

*M. rhizoxinica* that lack a functional T3SS are incapable of triggering visible spore formation, presumably since they are unable to consistently reinfect the apo-symbiotic host (17). Using fluorescence microscopy, we show that MTAL-deficient *M. rhizoxinica* strains are able to reinfect apo-symbiotic *R. microsporus* efficiently, yet their ability to induce fungal sporulation is profoundly impaired. Furthermore, the levels of apo-symbiotic *R. microsporus* reinfection by wild-type *M. rhizoxinica* are comparable. It follows that MTALs are T3SS-associated effectors that are not responsible for the absence of colonization by T3SS-deficient *M. rhizoxinica*. This is in stark contrast to *Xanthomonas* and *Ralstonia* TALs, which induce the expression of host plant genes that aid bacterial colonization and virulence (46). The nature of the T3 effectors responsible for establishment of the *Rhizopus-Mycetohabitans* symbiosis before and during invasion remains to be discovered. A recent transcriptomics study revealed upregulation of nearly 60 T3SS-associated effector genes in *M. rhizoxinica* upon initial physical contact with *R. microsporus* (18). Two candidate effectors were identified that may play a role during infection because they contain F-box-like and leucine-rich repeats (18), both features of known virulence effectors in pathogenic bacteria (47, 48).

Notably, this study provides the first functional characterization of T3SS-associated effector(s) in the maintenance of the *Rhizopus-Mycetohabitans* symbiosis. We demonstrate that MTAL proteins are crucial symbiosis factors that control fungal reproduction after colonization of the host. Reflecting their fundamental role in the maintenance of a stable *Rhizopus-Mycetohabitans* symbiosis, we show that *mtal* genes are prevalent in the genomes of eight endosymbiotic *Mycetohabitans* strains that were isolated from globally distributed *R. microsporus* strains inhabiting diverse habitats (20). Indeed, despite their heavily reduced genomes (22, 28), every endofungal *Mycetohabitans* strain analyzed so far contains at least one *mtal* gene (35). In addition to impaired fungal sporulation, we observed physiological changes in *R. microsporus* containing MTAL2- and MTAL3-deficient *M. rhizoxinica*. Fluorescence microscopy revealed septa formation by *R. microsporus*, an unusual phenomenon previously reported in *R. microsporus* reinfected with MTAL1-deficient *M. rhizoxinica* (19). Based on high-resolution live imaging, it was reported that absence of MTAL1 induces biogenesis of septa in *R. microsporus* leading to hyphal trapping of endobacteria and subsequent death of MTAL1-deficient *M. rhizoxinica* (19). It follows that the impaired sporulation of *R. microsporus* containing MTAL-deficient *M. rhizoxinica* may be an indirect consequence of the protective host response. The vital role of MTALs in the *Rhizopus-Mycetohabitans* endosymbiotic relationship is reinforced when one considers that the persistence of the symbiosis relies on spores containing healthy endobacteria (13).

In summary, we show that *M. rhizoxinica* MTALs do not promote colonization of *R. microsporus* but are essential factors in fungal sporulation (Fig. 6), representing an unprecedented case of bacterially produced T3SS effectors controlling host reproduction. The revelation that endobacteria affect the physiology of a fungal host by way of T3SS effectors offers a deeper insight into the dynamic interactions between bacteria and fungi. Our results illuminate a possible research avenue into the development of secretion system inhibitors to impede the *Rhizopus-Mycetohabitans* pathogenic alliance, which could potentially alleviate the economic damage caused by rice seedling blight.

## MATERIALS AND METHODS

### Strains and growth conditions

Eight *R. microsporus* strains harboring *Mycetohabitans* sp. endobacteria were used in this study (Table S1) (20). Endobacteria from *R. microsporus* ATCC62417 were eliminated by continuous antibiotic treatment (49), and the endosymbiont-free fungal strain was named apo-symbiotic *R. microsporus* (ATCC62417/S). Absence of endobacteria was confirmed by fluorescence microscopy and an absence of rhizoxin in extracts of the fungal mycelium (14). Both *R. microsporus* strains (ATCC62417 and ATCC62417/S) were cultivated on potato dextrose agar (PDA; Becton, Dickinson & Company, Sparks, MD, USA) at 30°C. Bacterial endosymbionts (M1-M8) were isolated from the mycelium of eight fungal strains as previously reported (50). Pure cultures of *M. rhizoxinica* were grown at 30°C in MGY M9 medium (10g/L glycerol, 1.25 g/L yeast extract, M9 salts: 40 mM K_2_HPO_4_, 14 mM KH_2_PO_4_, 2.2 mM C_6_H_7_NaO_7_, 7.5 mM (NH_4_)_2_SO_4_, and 0.8 mM Mg_2_SO_4_) or Standard Nutrient Agar I (Merck, Darmstadt, Germany) supplemented with 1% glycerol.

### *In silico* predictions and characterization of Type 3 effectors

Potential Type 3-secreted effector proteins were predicted using the T3SS PREDICTION server (51) and the EFFECTIVE Type 3 prediction tool (52). Nuclear localization sequences (NLSs) were predicted using the cNLS domain prediction tool (53) and NucPred (54). The nucleotide sequences of potential *Mycetohabitans* Type 3 effector genes have been deposited in GenBank under the accession numbers provided in Table S3.

### Amplification and Sanger sequencing of *Mycetohabitans* sp. *mtal* genes

Genomic DNA was isolated from eight axenic *Mycetohabitans* sp. cultures (Table S1) and quantified using a NanoDrop (Thermo Fisher Scientific, Waltham, MA, USA). PCR primers were designed to amplify partial coding sequences of *mtal1* (GenBank accession number: RBRH_01844; Table S4). PCRs were performed in 25.0 µL volumes containing 12.5 µL of high-fidelity Taq DNA polymerase (Phusion Master Mix, New England Biolabs, Ipswich, MA, USA), forward and reverse primers (both 0.4 µM), and 100 ng of template gDNA. The following thermocycling conditions were used for amplification: 98°C/30 s, 1 cycle; 98°C/10 s, 65°C/30 s, 72°C/3 min, 30 cycles; 72°C/7 min, 1 cycle; 16°C/hold.

The PCR products were visualized on a 1.5% agarose gel stained with ethidium bromide before gel extraction (Zymoclean Gel DNA Recovery Kit, Zymo Research, Irvine, CA, USA). The purified amplicons were ligated into pCR-Blunt II-TOPO (Invitrogen, Carlsbad, CA, USA), followed by transformation into chemically competent *Escherichia coli* TOP10 cells (Invitrogen, One Shot). The plasmids were purified (Monarch Plasmid Miniprep Kit, New England Biolabs), and plasmid inserts were bi-directionally sequenced by an external contractor (Eurofins Genomics, Ebersberg, Germany). Sequences were deposited in GenBank under the accession numbers provided in Table S3.

### Phylogenetic analysis

For phylogenetic analysis, predicted T3 effector protein sequences were aligned using ClustalW (55). Alignments were generated using a gap open penalty of 10 and a gap extension penalty of 0.1 as implemented in the MEGA7 package (Molecular Evolutionary Genetics Analysis software, version 5.0) (56). All positions containing gaps and missing data were eliminated. The evolutionary history was inferred using the Neighbor-Joining method with maximum composite likelihood distances and 10,000 bootstrap repetitions (57, 58). The alignments of sequences used in this study are shown in Fig. S1 and S3.

### Generation of *M. rhizoxinica mtal* mutant strains

To investigate the role of both AWR and MTAL proteins in the symbiosis, three genes (*awr*: RBRH_03012; *mtal2*: RBRH_01776; and *mtal3*: RBRH_01777) were deleted using a double crossover strategy as previously described (17). The gene *mtal1* (RBRH_01844) was previously deleted in *M. rhizoxinica*, yielding *M. rhizoxinica* Δ*mtal1*::Apra^R^ (*M. rhizoxinica* Δ*mtal1*) (19).

Using a proofreading polymerase, the upstream and downstream regions of the genes of interest were amplified. Primers were designed to contain 20 bp overlap with the gene of interest as well as a 20 bp overlap with an antibiotic resistance cassette (kanamycin). The kanamycin cassette was amplified from pK19, using primers carrying the same 20 bp overlaps (Fig. S2B).

The gene disruption vector pGL42a was used to generate *M. rhizoxinica* Δ*awr*::Kan^R^, Δ*mtal2*::Kan^R^, and Δ*mtal3*::Kan^R^ mutants. pGL42a was double-digested with the restriction enzymes SpeI and KpnI (New England Biolabs). The linear vector was gel-purified (Monarch DNA Gel Extraction Kit, New England Biolabs) and quantified on a NanoDrop (Thermo Fisher Scientific).

For each target gene, equimolar amounts of the three relevant PCR products were mixed with linear pGL42a in 2× Master Mix (NEBuilder HiFi DNA Assembly Cloning Kit, New England Biolabs) and incubated at 60°C for 1h following the manufacturer’s recommendations. The new plasmids pZU52, pZU21, and pZU19 (targeting *awr*, *mtal2*, and *mtal3* for disruption, respectively) were introduced into *E. coli* by chemical transformation. Transformants were selected on Standard Nutrient Agar I supplemented with 50 µg/mL kanamycin.

Competent *M. rhizoxinica* was transformed with vectors pZU52, pZU21, or pZU19 via electroporation (17). Transformants were grown on Standard Nutrient Agar I containing 50 µg/mL kanamycin. Colonies were subsequently passaged onto agar plates containing double selection medium (17) until the correct gene disruption vectors were observed using colony PCR. Colony PCRs were carried out in 12.0µL volumes containing 5µL of high-fidelity OneTaq Quick-Load 2× Master Mix (New England Biolabs), appropriate forward and reverse primers (both 0.4 µM; Table S4), and 5µL colony suspension. The following thermocycling conditions were used for amplification: 96°C/3min, 1 cycle; 96°C/10s, 58°C/15s, 68°C/1min, 30 cycles; 68°C/5min, 1 cycle; 16°C/hold. The resulting PCR products were visualized on an agarose gel. Primers were designed to span the two recombination sites, yielding amplicons A and B in mutant strains and amplicons C and D in *M. rhizoxinica* wild-type strains (Fig. S2A and B).

### Generation of genetically complemented *M. rhizoxinica* Δ*mtal1* strains

In order to genetically complement the MTAL-deficient strains *M. rhizoxinica* ∆*mtal2* and *M. rhizoxinica* ∆*mtal3*, genomic DNA from *M. rhizoxinica* was isolated using the MasterPure DNA Purification Kit (Biozym Scientific, Hessisch Oldendorf, Germany) following the manufacturer’s recommendations. The *mtal2* and *mtal3* genes were amplified by PCR with the primer pairs listed in Table S4 using Phusion High-Fidelity PCR Master Mix with HF Buffer (New England Biolabs). The PCR products were gel-purified with the Monarch DNA Gel Extraction Kit (New England Biolabs). The purified amplicons were cloned into the *Nde*I/*Afl*II restricted pBBR_P*_s12_*_*gfp* downstream of the constitutive promoter P*_s12_* with the 2× Master Mix (NEBuilder HiFi DNA Assembly Cloning Kit, New England Biolabs), yielding pBBR-*mtal2* and pBBR-*mtal3*. The reaction mixture was subsequently used to transform chemically competent *E. coli* TOP10 cells (Invitrogen, One Shot).

To generate an empty vector control, pBBR_P*_s12_*_*gfp* was digested with the restriction enzyme *BstB*I. The resulting linear vector lacking *gfp* was self-circularized using T4 DNA Ligase (New England Biolabs) to yield pBBR∅ and used to transform chemically competent *E. coli* TOP10 cells (Invitrogen, One Shot). All plasmids were purified from *E. coli* TOP10 overnight cultures using the Monarch Plasmid Miniprep Kit (New England Biolabs) and verified by restriction digest and Sanger sequencing using the primers *cm^r^*_seq_fw and BBR_seq_rv (Table S4).

The new plasmids (pBBR-*mtal2*, pBBR-*mtal3*, or pBBR∅) were introduced into competent *M. rhizoxinica* ∆*mtal2* or *M. rhizoxinica* ∆*mtal3* cells as appropriate via electroporation. Transformants were grown on Standard Nutrient Agar I containing 50µg/mL chloramphenicol and 50 µg/mL kanamycin. Colonies containing the respective plasmids were verified using colony PCR (Fig. S2D and E; see also Table S4). The complemented *M. rhizoxinica* Δ*mtal1* mutant (*M. rhizoxinica* Δ*mtal1* pBBR-*mtal1*) and *M. rhizoxinica* Δ*mtal1* containing the relevant empty vector (*M. rhizoxinica* Δ*mtal1* pBBR∅) were generated previously (19).

### Sporulation bioassay

In a liquid sporulation bioassay, apo-symbiotic *R. microsporus* aerial hypha (∼0.1 cm^3^) was grown in 24-well plates containing 750 µL Vorkultur medium (5 g/L glycerol, 10 g/L yeast extract, 10 g/L corn starch, 10 g/L corn step solids, 10 g/L CaCO_3_; pH 6.5). After 15 h of incubation, 100 µL of overnight cultures of *M. rhizoxinica* wild type (M1WT), *M. rhizoxinica mtal* mutants (*M. rhizoxinica* Δ*mtal1*, *M. rhizoxinica* Δ*mtal2*, and *M. rhizoxinica* Δ*mtal3*), *M. rhizoxinica mtal* mutants containing the relevant empty vector (*M. rhizoxinica* Δ*mtal1* pBBR∅**,** *M. rhizoxinica* Δ*mtal2* pBBR∅, and *M. rhizoxinica* Δ*mtal3* pBBR∅), or *M. rhizoxinica mtal* mutants expressing a plasmid-borne copy of the relevant *mtal* gene (*M. rhizoxinica* Δ*mtal1* pBBR-*mtal1*, *M. rhizoxinica* Δ*mtal2* pBBR-*mtal2*, and *M. rhizoxinica* Δ*mtal3* pBBR-*mtal3*) were added to individual wells. Co-culture plates were incubated at 30°C for 5–14 days. Fungal mycelium was transferred from co-culture plates to PDA petri dishes, which were incubated at 30°C for 5 days. Spores were harvested from PDA plate using 10 mL NaCl (0.15 M) and counted using a Thoma Chamber.

Experiments were performed four times independently (*n* = 4 biological replicates) with six technical replicates on each plate. Data are presented as means with grey bars marking ±1 standard error of the mean. Raw data from sporulation experiments were processed with MS Excel. GraphPad Prism 5.03 (GraphPad Software, La Jolla, CA, USA; https://www.graphpad.com/) was used for statistical analysis and graphing. Data from spore counts were compared between *M. rhizoxinica* strains using one-way analysis of variance and Tukey HSD test function in GraphPad. *P* values < 0.05 were considered statistically significant. The Brown-Forsythe test was used to test for equal variance and a *P* < 0.05 was considered significant.

### Fluorescence microscopy of co-cultures

One-week-old fungal-bacterial co-cultures were used to visualize the localization of the *M. rhizoxinica* strains. The bacterial cells were stained with 5 µM Syto 9 (Invitrogen), and fungal cells were counter-stained with 2 µg/mL calcofluor white (Fluka, Germany) for 5–10 min. Fluorescence microscopy was carried out using a Zeiss LSM 710 confocal laser-scanning microscope (Zeiss, Oberkochen, Germany), and images were captured using the Zeiss-Zen software.

## Data Availability

All data generated or analyzed during this study are included in the article and in the supporting files.

## SUPPLEMENTAL MATERIAL

Supporting Material mbio.01824-23-s0001.pdf Supplemental figures and tables.

ASM does not own the copyrights to Supplemental Material that may be linked to, or accessed through, an article. The authors have granted ASM a non-exclusive, world-wide license to publish the Supplemental Material files. Please contact the corresponding author directly for reuse.
