# Supplementary material for: Transcription activator-like effectors from endosymbiotic bacteria control the reproduction of their fungal host
Source: mBio. 2023 Nov 16;14(6):e01824-23. doi: 10.1128/mbio.01824-23 (PMC10746252; doi:10.1128/mbio.01824-23)
Supplement: Supporting Material — Supplemental figures and tables. [file mbio.01824-23-s0001.pdf]

Supplementary file 1 for

Transcription activator-like effectors from endosymbiotic bacteria control the reproduction of their fungal host

Ingrid Richter[a], Zerrin Uzum[a], Philipp Wein[a], Evelyn M. Molloy[a], Nadine Moebius[a], Timothy P. Stinear[b], Sacha J. Pidot[b], Christian Hertweck[a,c,*]

* Address correspondence to Christian Hertweck. christian.hertweck@leibniz-hki.de

This PDF file includes:

- Figures S1 to S3
- Tables S1 to S7
- SI References

```
                                          10        20        30        40        50        60        70        80        90       100
                                  ....|....|....|....|....|....|....|....|....|....|....|....|....|....|....|....|....|....|....|....|
M. rhizoxinica M1 (CBW77121)      MCTASPAVEHAGPKSNSEKALGSAARKPAATSRHTAPLDELRSKLGVHLRGKQPPASIGSTMPGRAVGSTMPARAIGVTPADRDTPGYSIDTVGVLGVDE
Mycetohabitans sp. M2 (MN840555)  MCTASPAVEHAGPKSNSKKALGSAARKPAATSRHTAPLDELRSKLGVHLRGKQPPASVGSTMPGRAVGSTMPARAIGVTPADRDTPGYSIDTVGVLGVDE
Mycetohabitans sp. M3 (MN840550)  VYTASPAVEHASPKSNRKKSLGSTAPKPAATSRRTAALHELRSKIGAPLRGKQPRPSAGAAIP---------ARAIGVTPADYDTPGYSIDTVGVLGVDE
Mycetohabitans sp. M4 (MN840552)  VYTASPAVEHAGPKSNRKKSLGSTAPKPAATSRRTAALHELRSKIGAPLRGKQPRPSVGAAIP---------ARAIGVTPADYDTPGYSIDTIGVLGVDE
M. endofungorum M5 (MN840553)     VYTASPAGEHAGPKSNHKKSLGFTAPKPAATSRRTAALHELRSKIGAPLRGKQPRPSVGAAIP---------ARAIGVTPADYDTPGYSIDTVGVLGVDE
Mycetohabitans sp. M6 (MN840556)  MCTASPAVEHAGPKSNSEKALGSAARKPAATSRHTAPLDELRSKLGVHLRGKQPPASIGSTMPGRAVGSTMPARAIGVTPADRDTPGYSIDTVGVLGVDE
Mycetohabitans sp. M7 (MN840551)  VYTASPAVEHAGPKSNRKKSLGSTAPKPAATSRRTAALHELRSKIGAPLRGKQPRSSVGAAIP---------ARAIGVTPADYDTPGYSIDTIGVLGVDE
Mycetohabitans sp. M8 (MN840554)  MCTASPAVEHAGPKSNSEKALGTAARKPASTSRHTAPLDELRSKLGIRLRGKQPPASVRSAMP---------ARAIGVTPTDHDTPGYNIDTVGVLGVDE
B. pseudomallei (ZP04890375)      ETRHLAVSLFTGEEGREALSGWIADFDPGGAARLQRMHQLMYAAAVVLKQNGRAAARLLRNYCG-----------MSTERPDLQLKRQSYSMQRVLVSAR
B. pseudomallei (ZP02502372)      ETRHLAMSLFTGEEGREALSGWIADFDPGGAARLQRMHQLMYAAAVVLKQNGRAAARLLRNYCG-----------MSTERPDLQLKRQSYSMQRVLVSAR
B. pseudomallei (ZP02494128)      EARHLAMSLFTGEEGREALSGWIADFDPGGAARLQRMHQLMYAAAVVLKQNGRAAARLLRNYCG-----------MSTERPDLQLKRQSYSMQRVLVSAR
X. euvesicatoria (WP011347306)    LAEANAEIDRLRSAYAQARDALQSDQAGNSANRPASRTLGSHLAAERNREVEALRTRLGRNENMLTLQRTHAERCRRHARRLQQRLDQVEAKLQQARHSS
X. campestris (AZR30894)          LARTNAQIEQLQSAYAQARDALQSEQASSSASAPAPRTLASHLAAEQRQEMQVLRTQLVRDEQMLSLQRVHAERCQRNARRLQERLAQVEAKLQQAQHSS
X. vasicola (AVQ09097)            LARTNAQIEQLQSAYAQARDALQSEQASSSASAPAPRTLASHLAAEQRQEMQVLRTQLVRDEQMLSLQRVHAERCQRNARRLQERLAQVEAKLQQAQHSS
X. citri (ARR12757)               LADANAEIDQLRSAYAQARDALQSDQASSSANRPAPRTLGSHLAAERNREIEALRTRLGRDENMLTLQRTHAERCQRNARRLQERLAQVDAKLQQARHSS
X. campestris (AKS22319)          LADTNAEIDQLRSAYAQARDALRSDPASSSANRPASRTLASELVAEQDREIEALRTRLGYDEKMLTLQGTHAERCRRNTRRLQARLAQVDAKLEQARHSS
X. oryzae (AKO19890)              LARTNAQIDQLQSAYAQARDALQSDQASSSASAPASRTLASHLAAEQSQEIQALRTQLARDEQMLSLQSVHAERCQRNARRLQERLVQVEATLKQAQHSS
X. oryzae (ACD59124)              LARTNAQIDQLQSAYAQARDALQSDQASSSTSAPASRTLASHLAAEQSQEIQALRTQLARDEQMLSLQSVHAERCQRNARRLQERLVQVEATLKQAQHSS
Burkholderia sp. (WP013592484)    -------GAKLAHASIERTAAFIAEGSAARGSLPMPKFMNPRAIRDTLRRLRNREANAAAREWGQTLDDHFMQPVIAVNRLFPIPADASREAADARQAAI
Burkholderia sp. (ADN59567)      EIAIGAHGAKLADASIARTTASLAAGDATRGALPVPKFMNPRAIRDTLRRLRNREANATAREWADTLDEHFMQPVIAVNRLFPVPANASREVADARQAAI
R. solanacearum (YP003748288)     MSATKP-RPEVLQSRPVPAGTRPEDLPGVPDLKAPQTAARQRKIDATAKAAFNPPTSIAESMRGHLGLGRTKVAPQSVSIAEAMSSEAGLQVHLARLIGQ
R. solanacearum (BAH04968)        ASKHSGRPVNTVADYSPPARTVSAPPDLPTTADRDRASRSLARTAGLVQRAQRGLQKLQGTRHARAPKPDASSTRLPVTVPDSAPRYRQLLAQGFAATQD
R. solanacearum (BAD42384)        ASKHSGRPVNTVADYSPPARTVSAPPDLPTTADRDRASRSLARTAGLVQRAQRGLQKLQGTRHARAPKPEASSTRLPVTVPDSAPRYRQLLAQGFAATQD
R. solanacearum (YP003747289)     AAINVTSVPGFTKTTIDPLP--IPKRPPPPSLGKTPRPAGQHKLDAIDRQVDNLTAKLYPPLPTGTRADPEAKRAIGSERAFASAVEALFVPSGASVEAK
R. solanacearum (WP013209172)     AAISVTAVPGFTKTTIDPLP--VPKRPPPPKLGKVPKPAGQHKLDGIDRQVHNLTEKLYPPLPAGLHSEPDAKRSIAHEWAFAGAVEALFVPSGASAEAA
R. solanacearum (WP011002069)     IIFPFYSKTGTTPTRAQPKPDVVPALKPAPRRMRPPAAPGRKHQRNVDAVVPPAMATSPRAEIRFPLTTADAQRYLRAQWRQAETGGRFDQAAVDRLTTT
R. solanacearum (WP003275417)     MATTGPERAKARPSYPIPAR---ADMPPAPPTGPAHTPARQRKIDAAVRATFNPGPSIAELLRHPLGR---KPAAAPVPITEAMGTAEGMQARLGQLFGS
R. solanacearum (WP003274509)     AAINVTSVPGFTKTTIDPLP--IPKRPPPPSLGKTPRPAGRHKLDAIDRQVDNLTSKLYPPLPTGTRGDPEAKRAIGSQRAFASAVEALFVPWGASVEAK
R. solanacearum (EAP71656)        MSATRP-RPEVLQSRPVPAGTRPEDLPGVPDLKAPQTAARQRKIDATAKAAFNPPTSIAESMRGHLGLGRTKVTPQSVSIAEAMGSEAGLQVHLARLIGQ
R. solanacearum (EAP71039)        MATTGPERAKARPSYPIPAR---ADMPPAPPTGPAHTPARQRKIDAAVRATFNPGPSIAELLRHPLGR---KPAAAPVPITEAMGTAEGMQARLGQLFGS
R. solanacearum (CBJ39728)        AAINVTAVPGFTKTTIDPLP--IPKRPPPPNLSKTPKPAGQHKLDRIDRQVDNLTRKLYPPLPSGQHGDPEAKRAIASGRAFASAVEALFVPSGVGANAV
R. solanacearum (CBJ35355)        MSATKP-RPEVLQSRPVPAGTRPEDLPSVPATGPAQPAARQRKIDATVKATFNPPSSIAESMRSHLGLGQAKVT-QAVSISEAMSSQAGLQVHLARLLGQ
R. solanacearum (CAD17997)        ---------NALKAYPPPALSEASALQPATAREQARAARSQAATAALVQRTQQGLQRMRGTDNSHAAASAEPGARQPVTVPESARQYRQLLAQQFAATRD
R. solanacearum (BAH47286)        IIFPFYSKTGTTPTRAQPKPDVVPALKPAPRRMRPPAAPGRKHQRNVDAVVPPAMATSPRAEIRFPLTTADAQRYLRAQWRQAETGGRFDQAAVDRLTTT
R. solanacearum (BAH47283)        AAINVTAVPGFTKTTIDPLP--IPKRQPPPNLPKAPKPAGQHKLDRIDRQVHNLTQKLYPPLPSGLHADPEAKRAIASSRAFVSAVEALFVPSGAGVYAV
R. solanacearum (BAH04967)        ---------NALKAYTPPALSEASALQPATAREQARAARSQAATAALVQRTQQGLQRMRGTDNSHAAASAEPGARQPVTVPESARQYRQLLAQQFASTRD
R. solanacearum (BAD42389)        MSATRP-RPEVLQSRPVPAGTRPEDLPSVPATGPAQPAARQRKIDATVKAAFNPSSSIAESMRSHLGLGQDKVARQSVSIAEAMSSEAGMRVHLARLLSQ
```

```
                                      110       120       130       140       150       160       170       180       190       200
                                  ....|....|....|....|....|....|....|....|....|....|....|....|....|....|....|....|....|....|....|....|
M. rhizoxinica M1 (CBW77121)      PRHWTRAQLLNHLVEANYPGPTLATAELAYQLHTSLDDGTGEVNAARVQLVLFAMHEAGLRDNTRAMMD-------------------------------
Mycetohabitans sp. M2 (MN840555)  PRHWTRAQLLNHLVEANYPGPTLATAELAYQLHTSLDDGTGEVNAARVQLVLFAMHEAGLRDNTRAMMD-------------------------------
Mycetohabitans sp. M3 (MN840550)  PRLWTRAQLLNHLVEANYPGPNLATAELAHQLHTGLDDGTGKVSAARAQLVLFAMHEAGLRD--------------------------------------
Mycetohabitans sp. M4 (MN840552)  PRLWTRAQLLNHLVEANYPGPNLATAELAHQLHTGLDDGTGKVSAARAQLVLFAMHEAGLRD--------------------------------------
M. endofungorum M5 (MN840553)     PRLWTRAQLLNHLVEANYPGPNLATAELAHQLHTGLDDGTGKVSAARAQLVLFAMHEAGLRD--------------------------------------
Mycetohabitans sp. M6 (MN840556)  PRHWTRAQLLNHLVEANYPGPTLATAELAYQLHTSLDDGTGEVNAARVQLVLFAMHEAGLRDNTRAMMD-------------------------------
Mycetohabitans sp. M7 (MN840551)  PRLWTRAQLLNHLVEANYPGPNLATAELAHQLHTGLNDGTGKVSAARAQLVLFAMHEAGLRD--------------------------------------
Mycetohabitans sp. M8 (MN840554)  PRYWTRAQLLNHLVEANYPGPNLVTAELAHQLHTGLDDGTGKVSAARAQLVLFAMHDAGVRD--------------------------------------
B. pseudomallei (ZP04890375)      GTSLDILGIPPSITGREHHAEQKSVYRNALAIAQYLHEKLDAAYKPDSLEDMLAAAQIVSAETLGGSGDRTEREYHFSR---------------------
B. pseudomallei (ZP02502372)      GTSLDILGIPPSITGREHHAEQKSVYRNALAIAQYLHEKLDAAYKPDSLEDMLAAAQIVSAEALGGSGDRTEREYHFSR---------------------
B. pseudomallei (ZP02494128)      GTSLDILGIPPSITGREHHAEQKSVYRNALAIAQYLHEKLDAAYKPDSLEDMLAAAQIVSAEALGGSGDRTEREYHFSR---------------------
X. euvesicatoria (WP011347306)    RINTEEVWQHEQTVNALEAANGDMQRVAEHAAAQPPADEARPVAAEIVADTMDALLGALPGFR-------EETIDPVSR---------------------
X. campestris (AZR30894)          RVNTKELWQHEQTVSALEAANGDMQRAVETAAAQPPANEARRAPDDSVADAMAALLDALPGFR-------EDTIGPMSR---------------------
X. vasicola (AVQ09097)            RVNTKELWQHEQTVSALEAANGDMQRAVETAAAQPPANEARRAPDDSVADAMAALLDALPGFR-------EDTIGPMSR---------------------
X. citri (ARR12757)               RINTEELWQYEQTVSALEAANGDMQRVVEHAAAHPPADEALPVAADIVADTMEALLEALPGFR-------EQTIDPVNR---------------------
X. campestris (AKS22319)          RINTEELWQQEQTVSVLEAADGDMQRGVEHAAAQLPADEARPVASDIVADTMQTLLDVLPGFR-------EETLDPVSR---------------------
X. oryzae (AKO19890)              RVNTTELWQQDHTVSALEAANGDMQRAVETAAAQSPADEARRAPDDSVAEAMAALLDALPGFR-------EDTIGPMSR---------------------
X. oryzae (ACD59124)              RVNTKELWQQDHTVSALEAADGDMQRAVETAAAQSPADEARRAPDDSVAEAMAALLDALPGFR-------EDTIGPMSR---------------------
Burkholderia sp. (WP013592484)    NRLLDGLTLRRPRPARADASLPPAVERLPQACVVPLAQALAEASDGDPILAARILDRLQQPF-------DIAG---------------------------
Burkholderia sp. (ADN59567)       DSLLDGLTLRRPRPAQADPTLPLTVDRLPQECVVPVADALAEASGGDPALAARILHRLQQPL-------DIGGLSGVSAIGDTSSTRDTAGTAENSGISG
R. solanacearum (YP003748288)     LVHAPAVHTLHQALLGGLPQGAESASTHPLAQHVLVAEALANACGGDAVRATAALEALKAGT-------FLPTSHAEGE---------------------
R. solanacearum (BAH04968)        AGAVGRLADRLRALAPPLAEEAG--ERTRYARPMLLAKVLEHTCGQDAAAALRVLDRLTGGM-------RLGAPTFAAPDG-------------------
R. solanacearum (BAD42384)        AGAVGRLADRLRALAPPLAEEAG--ERTRYARPMLLAKVLEHTCGQDAAAALRVLDRLTGGM-------RLGAPTFAAPDG-------------------
R. solanacearum (YP003747289)     AETAPHLHNLLRDLTRIAEAQPDAVDSTRYAGMLQCARALAAATGGNAADACAALAHLRTHF-------SLTDGSDNAP---------------------
R. solanacearum (WP013209172)     SR----LPDLLGHLKQVADAQPGVADATRYAGMLQCARALATATGGNAADACVALEHLRTHF-------SLTDGSGGAP---------------------
R. solanacearum (WP011002069)     LT-----ARLLATSAATGNGVHEDSGEKACARAIMVCDALAQATGGDPARAHDALLALLDTT-------SHADAR-------------------------
R. solanacearum (WP003275417)     RAGAPGMQALHTALLGTFAARLVSASARPFARHVLLAEALANASEGDAVRAMAALRVLQSGR-------FLPMLFPENQ---------------------
R. solanacearum (WP003274509)     AETARHLHNLLRDLTQIAETQPDAADSTRYAGMLQCARALVAATGGNAADACAALGHLRTHF-------SLTDGSDSES---------------------
R. solanacearum (EAP71656)        PAHAPAVRTLHRALLDRLPRVAESASAQPFAQHVLVTEALANACGGDAVRATAALRALKAGA-------FLPTLHAEGE---------------------
R. solanacearum (EAP71039)        RAGAPGMQALHTALLGTFAARLVSASARPFARHVLLAEALANASEGDAVRAMAALRVLQSGR-------FLPMSFPENQ---------------------
R. solanacearum (CBJ39728)        PG----LRNLIHGLARMADAQPDAVDTTRYAGMLQCARALATATGGRADEACRAFEHLREHF-------SLTDGSDGAA---------------------
R. solanacearum (CBJ35355)        PVHAPTVRAFHDALLDSLPQTTESASERPFAQHVLLAEALANACGGDTVRATAALHALKAGE-------FLPTSHAADE---------------------
R. solanacearum (CAD17997)        TGAVARLSARLEALAPP-ADAVG--EHLRYARPTLVAEALAHTCGHDAAAALRVLASLENEA-------RLDTAAFGTPDAPARA---------------
R. solanacearum (BAH47286)        LT-----ARLLATSAATGNGVHEDSGEKACARAIMVSDALAQATGGDPARAHDALLALLDTT-------SHADAR-------------------------
R. solanacearum (BAH47283)        PG----LQTLIDGLAGMAEAQPDAVDTTRYAGMLQCARALATATGGRADEACRALEHLRDRF-------SLTDGSNGAP---------------------
R. solanacearum (BAH04967)        TGAVTRLSARLEALAPP-ADAVG--EHLRYARPTLVAEALAHTCGHDAAAALRVLASLENEA-------RLDTAAFGTPDAPARS---------------
R. solanacearum (BAD42389)        PLHAPAVRALHGALLDSLPQATESASGRGFAQHVLLAEALVNACGGDTIRATAALRALKAGE-------FLPTAHGTGD---------------------
```

```
                                                     210       220       230       240       250       260       270       280       290       300
                                            ....|....|....|....|....|....|....|....|....|....|....|....|....|....|....|....|....|....|....|....|
M. rhizoxinica M1 (CBW77121)                -----------------------------STMATMVLQQLRLLNFDQPFHDAPTPTGDARLPDQQLQAWRASWETARVLARTEEGFETLLKLR-------
Mycetohabitans sp. M2 (MN840555)            -----------------------------STMATMVLQQLRVLNFDQPFHDAPTPAGDAHLPDQQLQAWRASWETARVLARTEEGFETLLKLR-------
Mycetohabitans sp. M3 (MN840550)            -----------------------------STTATMMLRQLRTLNFDQPFHDASAAAGGAHLPDQR-QAWRASWETARVLARTEYGFETLLKLR-------
Mycetohabitans sp. M4 (MN840552)            -----------------------------STTATMVLRQLRALNFDQPFHDASAAVGGAHLPDQR-QAWRASWETARVLARTEYGFETLLKLR-------
M. endofungorum M5 (MN840553)               -----------------------------STTATMVLRQLRTLNFDQPFHDASAAAGGAHLPDQQ-QAWRASWETARVLARTEYGFETLLKLR-------
Mycetohabitans sp. M6 (MN840556)            -----------------------------STMATMVLQQLRLLNFDQPFHDAPTPTGDARLPDQQLQAWRASWETARVLARTEEGFETLLKLR-------
Mycetohabitans sp. M7 (MN840551)            -----------------------------STTATMMLRQLRTLNFDQPFHDASAVAGGAHLPDQR-QAWRASWETARVLARTEYGFETLLKLR-------
Mycetohabitans sp. M8 (MN840554)            -----------------------------STTATKVLRQLRALNFDQPFHDVSAAAAGAHLPDQQ-QAWRASWETARVLARTEYGFETLLKLR-------
B. pseudomallei (ZP04890375)                --------------------------VVDNLGSIYQEERLSIDALACAANLISIDRDNGAKALGAVAMLSSSEQADVMDALEQAGELVRLLPLGIRRDIA
B. pseudomallei (ZP02502372)                --------------------------VVDNLGSIYQEERLSIDALACAANLISIDRDNGAKALGAVAMLSSSEQADVMDALEQAGELVRLLPLGIRRDIA
B. pseudomallei (ZP02494128)                --------------------------VVDNLGSIYQEERLSIDALACAANLISIDRDNGAKALGAVAMLSSSEQADVMDALEQAGELVRLLPLGIRRDIA
X. euvesicatoria (WP011347306)              -------------------------QVLRAWADGLHGRSAAFGGDALQPVDALRIALQALAIASEGAAAAAADVLRRLGMVRLRELIPPPQVSIAAEAAQ
X. campestris (AZR30894)                    -------------------------QILRTWAGGLQGRSAAFGTDALQPVDALNIALQALSIASEGDATEAANVLQRLGEVQLRELIPPPQVSNAAEAVP
X. vasicola (AVQ09097)                      -------------------------QILRTWAGGLQGRSAAFGTDALQPVDALNIALQALSIASEGDATAAANVLQRLGEVQLRELIPPPQVSNAAEAVP
X. citri (ARR12757)                         -------------------------QVLRAWADGLHGRSAAFGSDALQPVDALRIALQALAIASDGDATAAASTLQRLGVVQLRELIPPPQVSIAAEAVP
X. campestris (AKS22319)                    -------------------------QVLRVWADGLHARSAAFGTEALQPVDALRIALQALSIASGGDATAAANALQRLSVVRMRELIPPPQVSIAAEAVP
X. oryzae (AKO19890)                        -------------------------QVLRTWAEGLHRRSAAFGTDALQPVDALNIALQALSIASQSDATTAANVLQRLGEVQLRELIPPPQVSNAAEAVP
X. oryzae (ACD59124)                        -------------------------QVLRTWADGLHRRSAAFGTDALQPVDALNIALQALSIASQSDATTAANVLQRLGEVQLRELIPPPQVSNAAEAVP
Burkholderia sp. (WP013592484)              ----------------------------DAAHAAPDELERRAWRCAQDLAHARCAALEGLLRLQFGDGARHAERGNGAAARLVVYLQAGRKLR------S
Burkholderia sp. (ADN59567)                 TSDASGTSGTSNTSNTSNTTTIRAAPAQHPSQAAPADLEQRAWRCAQNLAHARCAALEGLLRLQYGDSARHAERGNGMAARLVVYLQAAHKLR------S
R. solanacearum (YP003748288)               ----------------TASPRPGNGGANGSRIGRMAPDGPGVRRPGNVGANTLHSAPQPAAEDAMLDAMHAAQELAGIGDVGMQALLALMPRLKPAKHEP
R. solanacearum (BAH04968)                  ----------------------------SPDAHEP-ALIRQASAASDAFFDALSDFHDAAPAAETGAAWRTAQLLARYHAGFDALCSLMQIPDVPEQRQA
R. solanacearum (BAD42384)                  ----------------------------SPDAHES-ALIRQASAASDAFFDALSDFHDAAPAAETGAAWRTAQLLARYHAGFDALCSLMHIPDVPEQRQA
R. solanacearum (YP003747289)               -----------------------PSPEQKQAWSAAKLLAHTASGFDALLALRPALAEVDAARPDDG-MNHRMKREGLRTFLQAADHLAARMPA-----DT
R. solanacearum (WP013209172)               -----------------------ATPAQKHAWSTAKLLAHTASGFDALLALRPALAEVDARHPDDG-MNGRMKREGLRTFLQASDLLAARMPAGVTPPQT
R. solanacearum (WP011002069)               --------------------------VRADVLALQTALGMTAIGLDTLLAIAPHVLPQR------GSAAPEIQREALRHALRAADHLLRRKPADAPGPTS
R. solanacearum (WP003275417)               ----------------PASP-LGAPGFSDTGAGALHPAR-------SSFPDDGMGLSPAQTARAKLDALHVGQALASIGDVGMQTLAASIPGLEPARHEP
R. solanacearum (WP003274509)               -----------------------PSPEQKHAWSAAKLLAHTASGFDSLLALRPALAEVDAVRPDDG-MNDRMKREGLRTFLQAADHLAARMPA-----DT
R. solanacearum (EAP71656)                  ----------------TASPRSSNAGADASRIARMALDGPGVRRTGSAGADALHSAPQPAAEDAMLDAMHAAQELAGIGDVGMQALLALMPRLKPARHEP
R. solanacearum (EAP71039)                  ----------------PASP-LRAPRFSDTGAGALHPAR-------SSFPDDGMGLSPAQTARAKLDALHVGQALASIGDVGIQTLAASIPGLEPALHEP
R. solanacearum (CBJ39728)                  -----------------------PTPAQTQAWSTAKLLAHTASGFDALLALRPALAKVDATLPDDDHMNGRMKREGLRTFLQAADLMAARLPAGVAPPQT
R. solanacearum (CBJ35355)                  ----------------TASPRTSHASVRIASAAQVAPNGPDTRGIDNADADTSPSTRRVLAKDAMLDAMHAAQELAGIGDVGMQALLALMPPLRPAKREP
R. solanacearum (CAD17997)                  ----------SVSSATFHDAISEQEGPDEPDGPPPQSPSARSSASSEAFFDAMSVLEEPGEPEDAGAAWRTAQLLSRYHAGFEALCDLMRLPQTPAHRHA
R. solanacearum (BAH47286)                  --------------------------VQADVLALQTALGMTAIGLDTLLAIAPHVLPQRGL--AEGSAAPEIQREALRHALRAADHLLRRKPADAPGPTS
R. solanacearum (BAH47283)                  -----------------------PIPAQMHAWSTAKLLAHTASGFDALLALRPALAEVDATLPDDDNMNGRMKREGLRTFLQAADLMAARLPAGVAPPQT
R. solanacearum (BAH04967)                  ----------SVSSATFHDAISEQEAPNEPDGPPPQSPSARSSASSEAFFDAMSVLEEPGEPEDAGAAWRTAQLLSRYHAGFEALCDLMRLPQTPAHRHA
R. solanacearum (BAD42389)                  ----------------AASPRPSDAGAEDAATAQAEPAGQHARRIGNASANAIHPTRQTPVEHAMLDAMRAAQGLAGIGDVGMQALLAVMPPLQPAKREP
```

	310 320 330 340 350 360 370 380 390 400
	....|....|....|....|....|....|....|....|....|....|....|....|....|....|....|....|....|....|....|....|
M. rhizoxinica M1 (CBW77121)	AEKCAPLSRSVDRDMLHVLLDSADRIDNHVRLTQPHDS------------HATPVHAAPDFDDPSPSGTDKRQQWLQGHLAMKGQDAKSTLLASQVFAIA
***Mycetohabitans* sp. M2 (MN840555)**	**AEKCAPLSRSVDRDMLHVLLDSADRIDNHVRLTQPHDS------------HATPVHAAPDFDDPSPSGTDKRQQWLQGHLAMNGQDAKSTLLASQVFAIA**
***Mycetohabitans* sp. M3 (MN840550)**	**AEKCAPLSRSADRDMLHVLLDTAEWIENHVRLAQQRDG------------HATSVHAALDFDDPSPSGTAKRQQWLQSHLAMNGQDAKATLLASQVFAIA**
***Mycetohabitans* sp. M4 (MN840552)**	**AEKCAQLSRSANRDMLHVLLDTAEWIENHVRLAQQRDG------------HATSVHAALDFDDPSPSGTAKRQQWLQSHLAMNGQDAKATLLASQVFAIA**
***M. endofungorum* M5 (MN840553)**	**AEKSAPLSRSADRDMLHVLLDTADRIETHVRLVQQRDG------------HATSVHAALDFDDPSPSGTAKHQQWLQSHLAMNGQDAKATLLASQVFAIA**
***Mycetohabitans* sp. M6 (MN840556)**	**AEKCAPLSRSVDRDMLHVLLDSADRIDNHVRLTQPHDS------------HATPVHAAPDFDDPSPSGTDKRQQWLQGHLAMKGQDAKSTLLASQVFAIA**
***Mycetohabitans* sp. M7 (MN840551)**	**AEKCAPLSRSADRDMLHVLLDTAERIENHVRLAQQRDG------------HATSVHAALDFDDPSPSGTAKRQQWLQSHLAMNGQDAKATLLASQVFAIA**
***Mycetohabitans* sp. M8 (MN840554)**	**AEKFAPLSRSADRDMLHVLLDTAERIENYVRFVQQHDG------------HATPMHAALDFDDSSPSGTAKRQQWLQGHLAMNGQDAKATLLASQVFAIA**
B. pseudomallei (ZP04890375)	TVADLDKVLNANGEGPGARASVSSVDPMGQASAQKAIGKLRAALAVAP-DIGNIPIGAAARAAIPMQQKIAYFKWRNG-LADERKARLVFERLYKLNKYA
B. pseudomallei (ZP02502372)	TVADLDKVLNANGEGPGARASVSSVDPMGQASAQKAIGKLRAALAVAP-DIGNIPIGAAARAAIPMQQKIAYFKWRNG-LADERKARLVFERLYKLNKYA
B. pseudomallei (ZP02494128)	TVADLDKVLNANGEGPGARASVSSVDPMGQASAQKAIGKLRAALAVAP-DIGNIPIGAAARAAIPMQQKIAYFKWRNG-LADERKARLVFERLYKLNKYA
X. euvesicatoria (WP011347306)	PMEIVTACVGLLASVPRGMQVLSHMLREEATPPSREWLEAAQVHLRASHRLALTPDAEQAERSWLGDAQMGARHAVHGDSPLQGLQSATDAQRSAFHAFR
X. campestris (AZR30894)	PMEAVTTCVGLLASVPRGMQVLLHTLCEQPTSPAREWLEAAQVYLRASHRLALTPDAEQAERAWLADAQMGARHAVHGATPLQGLQSATDAQRSAFHAFR
X. vasicola (AVQ09097)	PMEAVTTCVGLLASVPRGMQVLLHTLCEQPTSPAREWLEAAQVYLRASHRLALTPDAEQAERAWLADAQMGARHAVHGATPLQGLQSATDAQRSAFHAFR
X. citri (ARR12757)	TMETVTACVGLLASVPRGMQVLSHMLREEPTPPSREWLEAAQIHLRASHRLALTPDAEQAERTWLGDAQMGARHAVHGDSPLQGLQSATDAQRGAFHAFR
X. campestris (AKS22319)	PMETVTACVGLLASVPRGMQVLSHMLREEPTPASREWLEAAQVHLRASHRLALTQDAEQAERTWLGDAQMGARHAVHGASPLQGLQSATDAQRGAFHAFR
X. oryzae (AKO19890)	PMEAVTTCVGLLVSVPRGMQVLLHMLREEPTAPAREWLEAAQVHLRASQRLALTPDAQQAERAWLANAQMGARHAVHGATPLQGLQSATDAQRSAFHAIR
X. oryzae (ACD59124)	PMEAVTTCVGLLVSVPRGMQVLLHMLREEPTSPAREWLEAAQVHLRASHRLALTPDAEQAERAWLADAQMGARHAVHGATPLQGLQSATDAQRSAFHAIR
Burkholderia sp. (WP013592484)	IDADLGATPADLNHESTYRLFADDPYGRHAMLALDAARRLPP--------DAADAHDTG---ACEIEAITAYRLWQLG-FDESGPGSDLARASQLLFDGG
Burkholderia sp. (ADN59567)	MDADLGATPADLNDDAAYRMFIEDPYARHALLALDAARRLPP--------DAASVHDTG---DCEIEAITAYRLWQMG-FDESGPGSDLERAAQLLFDGG
R. solanacearum (YP003748288)	LECVLQAAEQVTTETKGAQVRLPDIIGHAEKVADTRNDTLACKVLR---AQVKALRAPDTYDALSRADKSAVFAWRQG-FRTDDRHSLLSQTQQRLAK-F
R. solanacearum (BAH04968)	AHAFLQAADALQHGSVKPAGSPQALLDKHPIGSGTPEESLAIK-------TLHGAAAVLRGEAPTPEQAGALFAWRQG-FREEGPGTALDKTKARIGR--
R. solanacearum (BAD42384)	AHAFLQAADALQHGSVKPAGSPQALLDKHPIGSGTPEESLAIK-------TLHGAAAVLRGEAPTPEQAGALFAWRQG-FREEGPGTALDKTKARIGR--
R. solanacearum (YP003747289)	PGALLQRARSHLASASDRHSLAGDGLAVHALLCAADLHADPG--------KAAHAIGDR-------TQVAAYVAWRSG-YREGGKGSALERSLGRMNK-F
R. solanacearum (WP013209172)	PHAQWQDAHNRLSHAADRTSLGGDGLVLNALLCAAKVHAAPH--------DAANAIDDR-------TQVAAYVAWRSG-YREGGKGTALERSLGRMNK-F
R. solanacearum (WP011002069)	LEALAALADPVLPAVERGRPGSSAGASVAPPGKAPAWLADPGNALAIKALHAANALRADPAAQCPPHLAQAYLAWRNG-FDREGPGTDLAKAQQRLFK-L
R. solanacearum (WP003275417)	LEFVLQSAERITQETRGRKVHLGEILAHADAVRGTSANTLACKVLR---AEVRGLGAEHPVSGLDRADKAAVFAWRQG-FRSDEKRSPLRRTQERFAK-F
R. solanacearum (WP003274509)	PGALLERARSHLASASDRHSLAGDGLAVHALLCAADLHADPS--------KAAHAIGDR-------TQVAAYVAWRSG-YREGGKGSALERSLGRMNK-F
R. solanacearum (EAP71656)	LECVLQAAEQITTETKGAQVRFPDIIAHAEKVAGTRDDTLACKVLR---AEVKALRTPGIYDALSRADKSAVFAWRQG-FRTDDRHSLLSQTQQRLAK-F
R. solanacearum (EAP71039)	LEFVLQSAERITQETRGRKVHLGEILAHADAVRGTSADTLACKVLR---AEVRGLGAEHPVSGLDRADKAAVFAWRQG-FRSDEKRSPLRRTQERFAK-F
R. solanacearum (CBJ39728)	PHAQLQDARARLVAATDRASLAGDGLALNALLCAAQVHAQPH--------EAAQAVKDP-------AQVAAYVAWRSG-YREGGKGSALERSVGRMHK-F
R. solanacearum (CBJ35355)	LEFVLQAAEQVAAETKGAQVRLPDIIEHAEKMAGTRGDTLACKVLR---AEVKALRAADTHDALSRADKSAVFAWRQG-FRSDDKHSLLSQTQQRLAK-F
R. solanacearum (CAD17997)	AHAFLQAADALQRGAAGQAASPQALLQRCPPGSGTPDDSLAIK-------TLHAAAAQLRGEALSPEQTGALFAWRQG-FRAEGPGSDLAKVKARTAK--
R. solanacearum (BAH47286)	LEALAALADPVLPAVERGRPGSSAGASVAPPGKAPAWLADPGNALAIKALHAANALRADPAAQCPPHLAQAYLAWRNG-FDREGPGTDLAKAQQRLFK-L
R. solanacearum (BAH47283)	PHAQLQDARARLDAAADRASLAGDGLALNALVCAAQVHAEPH--------EAAHAVDDR-------AQVAAYVAWRSG-YREGGKGSALERSLGRMHK-F
R. solanacearum (BAH04967)	AHAFLQAADALQRGAA----SPRALLQRCPPGSGAPDDSLAIK-------TLHAAAAQLRGEALSPEQTGALFAWRQG-FRAEGPGSDLAKVKARTAK--
R. solanacearum (BAD42389)	LEFVLQAAEQVSVETKGAQVLLPDIIGHAEKMAGTRNDTLACKVLR---AEVKALRAADTYDALSRADKSAVFAWRQG-FLTDDKHSLLSQTQQRLAK-F

```
                                                  410       420       430       440       450       460       470       480       490       500
                                         ....|....|....|....|....|....|....|....|....|....|....|....|....|....|....|....|....|....|....|....|
M. rhizoxinica M1 (CBW77121)             KKFFDQT-----------------------EPPHSASGPTPEGRFASFIETLTPNEKEALFDWQQR---FHESGRGTDKAKASGRLNNVRKVVTSIIGKL
Mycetohabitans sp. M2 (MN840555)         KKFFDQT-----------------------EPPHSASGPTPEGRFASFIETLTPNEKEALFDWQQR---FHESGRGTDKAKASGRLNNVRKVVTSIIGKL
Mycetohabitans sp. M3 (MN840550)         KKYFDQT-----------------------EHPHSTSGSTPEDRFASFIRTLAPSEKEALFDWQQR---FHESGRGTDKAKASGRLNNVRKVVTSIIGKV
Mycetohabitans sp. M4 (MN840552)         KKYFDQT-----------------------EHPHSTSGSTPEDRFASFIRTLAPSEKEALFDWQQR---FHESGRGTDKAKASGRLNNVRKVVTSIIGKV
M. endofungorum M5 (MN840553)            KKYFDQT-----------------------EPPHSTSGSTPEDRFASFIRTLAPSEKEALFDWQQR---FHESGRGTDKAKASGRLNNVRKVVTSIIGKV
Mycetohabitans sp. M6 (MN840556)         KKFFDQT-----------------------EPPHSASGPTPEGRFASFIETLTPNEKEALFDWQQR---FHESGRGTDKAKASGRLNNVRKVVTSIIGKL
Mycetohabitans sp. M7 (MN840551)         KKYFDQT-----------------------EHPHSTSGSTPEDRFASFIRTLAPSEKEALFDWQQR---FHESGRGTDKAKASGRLNNVRKVVTSIIGKV
Mycetohabitans sp. M8 (MN840554)         KKYFDQT-----------------------EHPHSASGSTPEDRFASFIKTLTPSEKEALFDWRQR---FHESGRGTDKAKASGRLNHFRKVITSIIGKV
B. pseudomallei (ZP04890375)             DRAIPRG-----------------------KNLTGRLRRIGDDIAGAFGHRKNPVGMLEEGLLLGG---------FLREDVFNEKIAPAFHSLIEKYRES
B. pseudomallei (ZP02502372)             DRAIPRG-----------------------KNLTGRLRRIGDDIAGAFGHRKNPVGMLEEGLLLGG---------FLREDVFNEKIAPAFHSLIEKYRES
B. pseudomallei (ZP02494128)             DRAIPRG-----------------------KNLTGRLRRIGDDIAGAFGHRKNPVGMLEEGLLLGG---------FLREDVFNEKIAPAFHSLIEKYRES
X. euvesicatoria (WP011347306)           NGYETTRAG--------------------SPYARVNACLQMFAQWAVAGSGRRGRRALNPFNALELG--MKVGASEALPTAVRRANDAFREAAGHLTSWL
X. campestris (AZR30894)                 NGYETTRAG--------------------SPYARVNASLQMFAQWAVAGGETRGKRALNPFNALKLG--MKVGASEALPTAVRRANDAFREAAGHLTSWL
X. vasicola (AVQ09097)                   NGYETTRAG--------------------SPYARVNASLQMFAQWAVAGGETRGKRALNPFNALKLG--MKVGASEALPTAVRRANDAFREAAGHLTSWL
X. citri (ARR12757)                      NGYETTGAG--------------------SPYARVNACLQMFAQWAVAGSAPRGKRALNPFNALALG--MKVGASEALPTAERRANDAFREAAGHLTSWL
X. campestris (AKS22319)                 NGYETTRTG--------------------SPYARVNACLQMFAQWAVAGSRRRGNSDLNPFNALALG--MKVGASEALPTAVRRANDAFREAAGHLTSWL
X. oryzae (AKO19890)                     NGYETTRAG--------------------SPYARVNASLQMFAQWAVAGGQTRGKRALNPFNALKLG--MKVGASEALPTAGRRANDAFREAAGHLSNWL
X. oryzae (ACD59124)                     NGYETTRAG--------------------SPYARVNASLQMFAQWAVAGGQTRGKRALNPFNALKLG--MKVGASEALPTAGRRANDAFREAAGHLSNWL
Burkholderia sp. (WP013592484)           TTWVARGAERQARKDRALGPIDGAPPAHRFAHARARIGLAVHDMPRAFGKNRSPIGVSSLLGADTPFFDTTRGRYDTALIAARDALLDYSHAQLPAAATP
Burkholderia sp. (ADN59567)              TTWVARGAERQARKDRALGPIDGAPPAQRFAQARARVGLAVHDVPRAFGRNRTPIDTSSLLGADTPFFDTTRGQYDSALIAAREALLDYSNEQLPCAATP
R. solanacearum (YP003748288)            RKYVSRAETRDKLDRMRHDP---------SQSTAATVRLVANNVQRAFSHKKSPLLGMGRYGALG----TGTRHVPTDLLELDKHLRTAIDELKGHVAAR
R. solanacearum (BAH04968)               --FVRR------------------------VIPRVEKHGWRTMLWQMIGKKASPLSSAR-LGLQA----AHRKNLAGEYKDYVDALRSAATTLAARYAQA
R. solanacearum (BAD42384)               --FVRR------------------------VIPRVEKHGWRTMLWQMIGKKASPLSSAR-LGLQA----AHRKNLAGEYKDYVDALRSAVTTLAARYAQA
R. solanacearum (YP003747289)            TAWARR------------------------AEHRANHRLAAFDPRRLLGMRKTPVIAAA-YGTGG----ANLGLLNGESKALYDTVQEGIEAMLAHSVVL
R. solanacearum (WP013209172)            TTWARR------------------------AEHRANHRLAAFDPRRLLGMRKSPLTAAA-YGTGG----ANLGLLNREAQALHDTVKDGLEAMLAHSTVL
R. solanacearum (WP011002069)            FAYAER------------------------AARTG---MAARAAAGFFGTQKSPLSALQNFGTAG----VMLGHPDDEFARFTAALAP---VKTQLADRL
R. solanacearum (WP003275417)            RKYVARAEKRDALNKMHFDPRNPI--RSAGKLATKTAGLVTNNVQRVFSHQKSPLLGLGKYGALA----AGAQLAPTDQVRLDEHLITAMGELKAYLESS
R. solanacearum (WP003274509)            TAWARR------------------------AEHRANHRLAAFDPRRLLGMRKTPVIAAA-YGTGG----ANLGLLNGESKALYDTVQEGIEAMLAHSVVL
R. solanacearum (EAP71656)               RKYVSRAETRDKLDRMRHDP---------SQSTAATARLVANNVQRAFSHKKSPLLAMGRYGTLG----TGTRHVPTDLLELDKHLRTAIDALKAHVEAR
R. solanacearum (EAP71039)               RKYVARAEKRDALNKMHFDPRNPI--RSAGKLATKTAGLVTNNVQRVFSHQKSPLLGLGKYGALA----AGAQLAPTDQVRLDEHLITAMGELNAYLKSS
R. solanacearum (CBJ39728)               TTWARR------------------------AEHRATHPLAAFDPRRLLGMRKSPLTAAA-YGTGG----ANLGLLNQEAQALHDTVKRGIEAMQAHSTEL
R. solanacearum (CBJ35355)               RKYVSRAETRDKLDKARHDPGN------PMQSSVATARLVANNAQRVFSHKKSPLLAMGKYGSLA----AGTKHVPMDQVELDKHLRTAIDALRVRLEAQ
R. solanacearum (CAD17997)               --FVSR------------------------TIRRVENHGWRSTLWQVVGKKKSPLSAAR-LGMQA----AHRVSLDKEYTAYAASLRTALGMLGARYREA
R. solanacearum (BAH47286)               FTYAER------------------------AARTG---MAARAAAGFFGTQKSPLSALQNFGTAG----VMLGHPDDEFARFTAALAP---VKTQLADRL
R. solanacearum (BAH47283)               TTWARR------------------------AEHRATHPLAAFDPRRLLGMRKSPLTAAA-YGTGG----ANLGLLNQEAQALHDTVKHGIEAMQAHSTAL
R. solanacearum (BAH04967)               --FVNR------------------------TIRRVENHGWRSTLWQVVGKKKSPLSAAR-LGMQA----AHRVSLAKEYTAYAASLRTALGMLGARYREA
R. solanacearum (BAD42389)               RKYVSRAETRNALNEARQDP---------ARSTAATARLVANNVQRAFSRKKSPLLAMGKYGSLA----AGTRHVPTDQVELDKHMRTAIDALKDRLQTR
```

```
                                      510       520       530       540       550       560       570       580       590       600
                                  ....|....|....|....|....|....|....|....|....|....|....|....|....|....|....|....|....|....|....|....|
M. rhizoxinica M1 (CBW77121)      VPGEVAAFFNRRPGLHETDKAKASHRLNKFVSEVATRDREHYGSHLIQRIFGTMKAP-----MVAATRIKSGAGKATE----------------------
Mycetohabitans sp. M2 (MN840555)  VPGEVAAFFNRRPGLHETDKAKASHRLNKFVSEVATRDREHYGSHLIQRVFGTMKAP-----MVAATRIKSGAGKATE----------------------
Mycetohabitans sp. M3 (MN840550)  VPAKVAALFNQRPGLHETDKAKASHRLNKFVSEVALRDREHCGSHLIQRIRGAMKAP-----MIAT-RIPSGASRAAE----------------------
Mycetohabitans sp. M4 (MN840552)  VPAKVAALFNQRPGLHETDKAKASHRLNKFVSEVALRDREHCGSHLIQRIRGAMKAP-----MIAT-RIPSGASRAAE----------------------
M. endofungorum M5 (MN840553)     VPGKVAALFNQRPGLHETDKAKASHRLNKFVSEVAPRDREHCGSHLVQRIRGAMKAP-----MIAT-RIPSGASRAAE----------------------
Mycetohabitans sp. M6 (MN840556)  VPGEVAAFFNRRPGLHETDKAKASHRLNKFVSEVATRDREHYGSHLIQRIFGTMKAP-----MVAATRIKSGAGKATE----------------------
Mycetohabitans sp. M7 (MN840551)  VPAKVAALFNQRPGLHETDKAKASHRLNKFVSEVALRDREHCGSHLIQRIRGAMKAP-----MIAT-RIPSGASRAAE----------------------
Mycetohabitans sp. M8 (MN840554)  VPGEVAALFNQRPDLRETDKAKASHRLNKFVSEVALRDRKHCGSHLIQRIWGAMKAP-----MIAT-RIPSGASRAAE----------------------
B. pseudomallei (ZP04890375)      GAGAGAGAGAGAGAGVAKARSEVVSRVFEKLKIDGFSSKIEIKSDDLKKWVALAVAG------GRNDAVKRSLEFESIR----------------RDLKN
B. pseudomallei (ZP02502372)      ----GAGAGAGAGAGVAKARSEVVSRVFEKLKIDGFSSKIEIKSDDLKKWVALAVAG------GRNDAVKRSLEFESIR----------------RDLKN
B. pseudomallei (ZP02494128)      ----GAGAGAGAGAGVAKARSEVVSRVFEKLKIDGFSSKIEIKSDDLKKWVALAVAG------GRNDAVKRSLEFESIR----------------RDLKN
X. euvesicatoria (WP011347306)    VARRQIQLAGGRMPSQDELAMQALLEYVQWLPHEQNATDLTFTAKVLGTIERRAQELQRSVAASVNETDDLQPAACHPAIAS-AWGALRAGRMRLPETLR
X. campestris (AZR30894)          VARRHTQSACGQLPSQDELAMQALSEYVQWLPHEQNATDLIFTAKVLAEIEQRALELQDSVTASLSTTDDRQPAACHPAIAS-AWVALRTGRVRLPETLR
X. vasicola (AVQ09097)            VARRHTQSACGQLPSQDELAMQALSEYVQWLPHEQNATDLIFTAKVLAEIEQRALELQDSVTASLSTTDDRQPAACHPAIAS-AWVALRTGRVRLPETLR
X. citri (ARR12757)               VARRQIQRAGGRMPSQDELAMQALLEYAQWLPHEQNATDLTFTAKVLGTIERRAQELQRSVAASVNEADDLQPAACHPAIAS-AWAALRAGRMRLPETLR
X. campestris (AKS22319)          VARRQIQLAGGRMPSQDELAMQALSEYAHWLPHEQNATDLTFTAKVLGTIERRAQELQRSVAASASESDDLQPAACHPAIAS-AWVALRAGRMRLPETLR
X. oryzae (AKO19890)              VARRHIQMTCGQLPSQDELAMQALAECVQWLPHEQSATELIFTAKVLAEIEQRARELQDSVTASLSTTDARQPAACHPAIAS-AWVALRTSRMRLPETMR
X. oryzae (ACD59124)              VARRHIQMTCGQLPSQDELAMQALAECVQWLPHEQSATELIFTAKVLAEIEQRARELQDSVTASLSTTDARQPAACHPAIAS-AWVALRTSRMRLPETLR
Burkholderia sp. (WP013592484)    GRMLTHALNAERLQAWSAARPAVPSDAPLRELQKRRPESFEIGRRDARRMWDAARARVAALRGQGDERMERTVGRTLRMFDD----RERRRRFIDDMTEG
Burkholderia sp. (ADN59567)       GRMLTHALNAQRLQAWTDARPDVPAHAKPRELQKRRPESFALGRREARRMWDAARARVAGLRVEGDQRTQRNVERALRMFDD----PQRRRRFVDDVTSS
R. solanacearum (YP003748288)     SGKAQFSIGRHGEVPIVPLRAAILEHWSAASADQ-RPQGYTLDGNAVVDIAEGIRR------ATGKSVVGADGRLPAQ---------------LEQLIGT
R. solanacearum (BAH04968)        EAAPLDPPGALPWRSPQEMWSSAVLKHWASALAQASPQDCVLTNDILAAIGRQLRDTVTRAFDALAAGIAQATEHDDGLREH---LRASLATLTRLSQGA
R. solanacearum (BAD42384)        EAAPLDPPGALPWRSPQEMWSSAVLKHWASALAQASPQDCVLTNDILAAIGRQLRDTVTRAFDALAAGIAQATEHDDGLREH---LRASLATLTRLSQGA
R. solanacearum (YP003747289)     RRLDGGHALPTERRGLLLLREAVLEHWRAQIGTTWRSSKLKLTDADKQGIARAVR------SAAHGTDIDAEAVLGYR--------EFRKLDKLDLKTLA
R. solanacearum (WP013209172)     RQPGGGHSLSTEQRGLLLLREAVLKHWGAHIDPTWRASKLKLTDADKRDIAQAVR------SAAHGTGIDAKAVLSYR--------EFRKLDKLDLMTLA
R. solanacearum (WP011002069)     KAASTSRALKTR----CAVRLAAIEQWERRMASKGLRSTFRFSSRDLETVAARARPLLSNRVALHADGSPADLALDPHRNRDAIRAEVKPLRGMTPAQLR
R. solanacearum (WP003275417)     DAPARAVQR--GAIPLPVLRAAVLAHWADASETR-RPQGHVLDAAAVADIAGRLQLY---PAATATQSADVAKRLPTQ---------------LTSLIGT
R. solanacearum (WP003274509)     RRLDGGHALSTERRGLLLLREAVLEHWRAQIGTTWRSSKLKLTDADKRDIARAVR------SAAHGTDVDAEAVLGYR--------EFRKLGKLDLKTLA
R. solanacearum (EAP71656)        SGAAQFNIGRHGEVPTVTLRAAILEHWSAASADR-RPQGYTLDGNAVVDIAEGIRR------ATGKSVVGADGRLPAQ---------------LEQLIGT
R. solanacearum (EAP71039)        DAPARAVQR--GAIPLPVLRAAVLAHWADASETR-RPQGHVLDAAAVADIAGRLQLY---PAATATQSADVAKRLPAQ---------------LTSLIGT
R. solanacearum (CBJ39728)        RGLNDRHALSTEQRGLLLLREAVLQHWGASIGTTWRSSKLKLSDHDKRAIADRVR------KAAPGARVDAKDVLTYR--------EFRKLDKLDLKTLA
R. solanacearum (CBJ35355)        SGEARFSVGRHGEVPTVILRTAILEHWSAASADK-RPQGYTLDGNAAVHIAECIHR------ATGQSVVRADGKLPKQ---------------LERLIGT
R. solanacearum (CAD17997)        LALSP-AHADLPWKQAEQMWQGAILQHWASLPADAPLSAYVLTHDTLRTIGEQLRHTVLRVREELTISLAHSAEAGDTPDPG---MLDKLRTLVELSRQA
R. solanacearum (BAH47286)        KAASTSRALKTR----CAVRLAAIEQWERRMASKGLRSTFRFSSRDLEAVAARARPLLSNRVALHADGSPADLALDPHRNRDAIRAEVKPLRGMTPAQLR
R. solanacearum (BAH47283)        RALNGRHALSTEQRGLLVLREAVLQHWAQSIGTTWRSSKLKLSDHDKRAIADWVR------KAAPGARVDAEAVLAYR--------EFRKLDKLDLKTLV
R. solanacearum (BAH04967)        LALSP-AHADLPWKQAEQMWQGAILQHWASLPADAPLSAYVLTHDTLRTIGEQLRHTVLRVREELTISLAHAAEAGDTPDPG---MLDKLKTLVELSRQA
R. solanacearum (BAD42389)        SGEARFSLGRYGEVPTVALRGAILEHWSATSESK-RPQGYTLDGNAMVDIAERLHR------AIGESVVRADGRLPQQ---------------LERLIGT
```

```
                                             610       620       630       640       650       660       670       680       690       700
                                    ....|....|....|....|....|....|....|....|....|....|....|....|....|....|....|....|....|....|....|
M. rhizoxinica M1 (CBW77121)        --ESSAADAQASAPD-------------------YASAASTAGAAEPTRAASTAIQSI-TTAVADASDRGWDALKRIVPSLRPGMAVEFSDGGALTVTTE
Mycetohabitans sp. M2 (MN840555)    --ESSAADAQASAPD-------------------YASAASTAGAAEPTRAASTATQSI-TTAVADASDSGWDALKRIVPSLRPGMAVEFSDGGALTVTTE
Mycetohabitans sp. M3 (MN840550)    --KSSAADMQASASD-------------------HASAASTAEASELTRAASTATQSV-TTAVADATDSGWDALKHIVPSLRPGMAVEFSDGGELAVTTE
Mycetohabitans sp. M4 (MN840552)    --KSSAADMQASAPD-------------------DASAASIAEASELTRAASTATQSV-TTAVADATDSGWDALKHIVPSLRPGMAVEFSDGGELAVTTE
M. endofungorum M5 (MN840553)       --KSSAADLQASAPD-------------------HASAASTTEASELTRAASTATQSV-TTAVADATDSGWDALKHIVPSLRPGMAVEFSDGGELAVTTE
Mycetohabitans sp. M6 (MN840556)    --ESSAADAQASAPD-------------------YASAASTAGAAEPTRAASTAIQSI-TTAVADASDRGWDALKRIVPSLRPGMAVEFSDGGALTVTTE
Mycetohabitans sp. M7 (MN840551)    --KSSAADMQASASD-------------------HASAASTAEASELTRAASTATQSV-TTAVADATDSGWDALKHIVPSLRPGMAVEFSDGGELAVTTE
Mycetohabitans sp. M8 (MN840554)    --KSSAADRQASAPD-------------------HASAASTAEASELTRAASTATQSV-TTAVTDATDSGWDALKRIVSSLRPGMAVEFSDAGKLTFTTE
B. pseudomallei (ZP04890375)        GFSVDVLERWAAEAG----------------------LDDGNFRRQLNDLRSVSNNGYGEIPELGDRASVFSAFSEIVARYRAGKSLKFSSGGVGGLALG
B. pseudomallei (ZP02502372)        GFSVDVLERWAAEAG----------------------LDDGNFRRQLNDLRSVSNNGYGVIPELGDRASVFSAFSEIVARYRAGKSLKFSSGGVGGLALG
B. pseudomallei (ZP02494128)        GFSVDVLERWAAEAG----------------------LDDGNFRRQLNDLRSVSNNGYGEIPELGDRASVFSAFSEIVARYRAGKSLKFSSGGVGGLALG
X. euvesicatoria (WP011347306)      LIQRGLLDHAGQPSDLPVG--------AATLDEELASDARTHFHQAIGHASRLLHEGD--TSRVRSPRALFECLRNLLERLEWRDKLRLTEQRVIGLNTT
X. campestris (AZR30894)            LIQRGLLDHPVQPIDTPDG--------AAHLGKELASVARNNFHQAVGNASRFLHEGD--TSRVRSPRALFACLRNVMERLEWRDKLRITEQRVIGLNTT
X. vasicola (AVQ09097)              LIQRGLLDHPVQPIDTPDD--------AAPLGKELASVARNNFHQAVGNASRFLHEGD--TSRVRSPRALFACLRNVMERLEWRDKLRITEQRVIGLNTT
X. citri (ARR12757)                 LIQRGLLDHAGQPSDLPVG--------AAPLDEELASVARDHFHRAVGHASRFLHEGD--TSRVKSPRALFECLRNLMERLEWRDKLRLTEQRVIGLNTT
X. campestris (AKS22319)            LIQRGLLDHAGQPTDMPIG--------AAPLDEELASVARNHFHQAVGHASRFLHEGD--TSRVRSPRALFECLRNLMERLEWRDKLRITEQRVIGLNTT
X. oryzae (AKO19890)                LIQRGLLDHAIQPNDTPAG--------AAPLDKELASLARNHFHQAVGNASRFLHEGD--TSRVRSPRALFACLRNMMERLEWRDKLRITEQRVIGLNTT
X. oryzae (ACD59124)                LIQRGLLDHAIQPNDTPAG--------AAPLDKELASVARNHFHQAVGNASRFLHEGD--TSRVRSPRALFACLRNVMERLEWRDKLRITEQRVIGLNTT
Burkholderia sp. (WP013592484)      GFTLAALESWFNASG---------------MAAADAARAEPTARHLQAAHDLLANPGI-APLDRITTRNLRKQHLALMAQNVPGTGFTSYDGNALGIDAS
Burkholderia sp. (ADN59567)         GFTLAALERCFNASG---------------MAADHPVRAQPAQRNLQAAHDLLDNPGI-APLQRITTHNLREQHLALMAQNIPGTGFTSYDGNALGIDAS
R. solanacearum (YP003748288)       RLNHATLKEWARDAA---------------MPQRTETGEETAFSSAMRRARNILRPDK-DKPADMSADSMRDYLKNFMADHNIGNSMTFADGGALGVNTS
R. solanacearum (BAH04968)          GLPP-ARDSPIHPPPGLIGLAPSGALLRSWSP----AAPAGPLEKALKSAERIEQQDD-LQLREKTIEAARELIESLLTTIDAGGKLRLASGSALGVNTG
R. solanacearum (BAD42384)          GLPP-ARDSPIHPPPGLIGLAPSGALLRSWSP----AAPAGPLEKALKSAERIEQQDD-LQLREKTIEAARELIESLLTTIDAGGKLRLASGSALGVNTG
R. solanacearum (YP003747289)       KWIDEAASADSNPAA---------------SPMLRRAKAKIVEAGDIARGRPIKLKGT-TLADFRDAMTDAIGTMPLGNYVRYFDGGTYGLNANMTVNQH
R. solanacearum (WP013209172)       RWLDEAASFDPDPSA---------------SPTLHLARTKIAEAGGIERGRPIKPQGA-TMADFRNAMTGAIDAMPLGNYVRYFDGGTYGASINMTVNQH
R. solanacearum (WP011002069)       TWADEAWRLSGQAVP---------------EPIR--ANIDLVES---RLAGDIRPKPG-DTNAQLDAIGALVKQMPDIYDIRVSSGGTVGLGGVPSQSLA
R. solanacearum (WP003275417)       RLTHATLEHWGHDAA---------------MPQRSVDGKETVFAGAMRRARNILDPGK-DVPADMTADSMRAFLKNFVIEHNGGNSMTFTDGGALGINTG
R. solanacearum (WP003274509)       KWIDEAASADSNPSA---------------SPMLRRAKAKIVEAGDIARGRPIKLKGT-TLADFRDAMTDAIGTMPLGNYVRYFDGGTYGLNANMTVNQH
R. solanacearum (EAP71656)          RLDHATLGTWAHDAA---------------MPQRTETGEETAFSGAMRRARNILRPDQ-DKPADMTADSMRDHLKNFMADHNIGNSMTFADGGALGVNTS
R. solanacearum (EAP71039)          RLTHATLEHWGHDAA---------------MPQRSVDGKETVFAGAMRRARNILDPGK-DVPADMTADSMRAFLKNFVIEHNGGNSMTFTDGGALGINTG
R. solanacearum (CBJ39728)          RWLDEARAFDSRASE---------------SPMLREAAEDLVKAGDIERGRPIKPQGT-TLADFREALTGAIDQMPLGNYVRYFDGATYGANTNMTVNQH
R. solanacearum (CBJ35355)          QLSHATLKAWARDAA---------------MPQRTETGEETDFSSAMRRARNILKPGR-DKPLDMTAGNMREFLKNFMAEHNVGNTMTFTDGGALGVNTG
R. solanacearum (CAD17997)          NLNLGASNLPVTPPPQFIDAYPSGAVLRKWSEQVDAGAASGPLRKALDTAERIEQQDD-LKLQEAAIDAARGVIESLITTIEGGGKLRLASGTSLGVNTG
R. solanacearum (BAH47286)          TWADEAWRLSGQAVP---------------EPVR--ANIDLVES---RLAGDIRPKPG-DTNAQLDAIGALVKQMPDIYDIRVSSGGTVGLGGVPSQSLA
R. solanacearum (BAH47283)          RWLDEARAFDPRASE---------------SPTLRAAADNIVKAGDIERGRPIKPRGT-TLADFREALTGAIGQMPLGNYVRYFDGATYGVNTNMTVNQH
R. solanacearum (BAH04967)          NLNLGASNLHVAPPPQFIDAYPSGAVLRKWSEQVDAGAASGPLRKALDTAERIEQQDD-LKLQEAAIDAARGVIESLITTIEGGGKLRLASGTSLGVNTG
R. solanacearum (BAD42389)          RLSHATLATWARDAA---------------MPQRTETGEETAFSSAMRRARNILEPGK-DKPVDMTADSMRAFLKNFTGEHNVGNAMTFTDGGALGVNTG
```

```
                                          710       720       730       740       750       760       770       780       790       800
                                 ....|....|....|....|....|....|....|....|....|....|....|....|....|....|....|....|....|....|....|....|
M. rhizoxinica M1 (CBW77121)     RVSASVTAAVSQATQASPVPLPVVVPSINAKRSRKRKATVQMG-CTEQHAWLFIGTAHATGRGAGAGVLVGASFPGSLVGGTVGVQF--------YDSEH
Mycetohabitans sp. M2 (MN840555) RVSASVTAAVSQAAQASPVPLPVVVPSINAKRSRKRKATVQMG-CTEQHAWLFIGTAHATGRGAGAGVLVGASFPGSLVGGTVGVQF--------YDSEH
Mycetohabitans sp. M3 (MN840550) RVSASVTAAVSQAAQASPVPLPVVVPSINAKRSRKRKATVQMG-CTEQHAWLFIGTAHATGDGAGVGVLVGASLPGSLVGGTVGVQF--------YDSEH
Mycetohabitans sp. M4 (MN840552) RVSASVTAAVSQAAQASPVPLPVVVPSINAKRSRKRKATVQMG-CTEQHAWLFIGTAHATGDGAGVGVLVGASLPGSLAGGTVGVQF--------YDSEH
M. endofungorum M5 (MN840553)    RVSASVTAAVSEAAQASPVPLLVVVPSINAKRSRKRKATVQMG-CTEQHAWLFIGTAHATGDGAGVGVLVGASLPGSLVGGAVGVQF--------YDSEH
Mycetohabitans sp. M6 (MN840556) RVSASVTAAVSQATQASPVPLPVVVPSINAKRSRKRKATVQMG-CTEQHAWLFIGTAHATGRGAGAGVLVGASFPGSLVGGTVGVQF--------YDSEH
Mycetohabitans sp. M7 (MN840551) RVSASVTAAVSQAAQASPVPLPVVVPSINAKRSRKRKATVQMG-CTEQHAWLFIGTAHATGDGAGVGVLVGASLPGSLVGGTVGVQF--------YDSEH
Mycetohabitans sp. M8 (MN840554) RVSASITAAVSQAAQASPVPLPVVVPSINTKRSRERKATVQMG-CTEQHAWLFIGTAHATGDGAGVGVLAGASLPGSLVGGTTSVQF--------YDSEH
B. pseudomallei (ZP04890375)     DSQSIGPLVSVSPT---------------LGAGGGRVATVEIGGASGRGGLIAVTRANSQKLKVGASVFAGPQFIHVVRAGGSANLELVS-------AEQ
B. pseudomallei (ZP02502372)     DSQSIGPLVSVSPT---------------LGAGGGRVATVEIGGASGRGGLIAVTRANSQKLKVGASVFAGPQFIHVVRAGGSANLELVS-------AEQ
B. pseudomallei (ZP02494128)     DSQSIGPLVSVSPT---------------LGAGGGRVATVEIGGASGRGGLIAVTRANSQKLKVGASVFAGPQFIHVVRAGGSANLELVS-------AEQ
X. euvesicatoria (WP011347306)   PLSAALAIIPSGLG---------LKLSAGGQTSSDRSVEIYMG---RTGLSLQIGRQKARQFNASTGISAGVLLPGTEHAPVGITGAAEWR----MKKES
X. campestris (AZR30894)         PLSAALAIIPSGLG---------LRLSAGGQISSDRSVEIYMG---RTGLSLQIGQQKARQFQASTGINAGVLLPGTERAPVGITGAADWR----IKKES
X. vasicola (AVQ09097)           PLSAALAIIPSGLG---------LRLSAGGQISSDRSVEIYMG---RTGLSLQIGQQKARQFQASTGINAGVLLPGTERAPVGITGAADWR----IKKES
X. citri (ARR12757)              PLSAALAIIPSGLG---------LKLSAGGQTSSDRSVEIYMG---RTGLSLQIGRQKARQFNASTGISAGVLLPGTEHAPVGITGAAEWR----MKKES
X. campestris (AKS22319)         PLSAALAIIPSGLG---------LKLSAGGQTSSDRSVEIYMG---RTGLSLQIGRQKARQFNASTGISAGVLLPGTERAPVGITGAAEWR----MKKES
X. oryzae (AKO19890)             PLSAALAIIPSGLG---------LKLSAGGQVSSDRSVEIYMG---RTGLSLQIGQQKARQFQASTGISAGVLLPGTEHAPVGITGAAEWR----IKKES
X. oryzae (ACD59124)             PLSAALAIIPSGLG---------LKLSAGGQVSSDRSVEIYMG---RTGLSLQIGQQKARQFQASTGISAGVLLPGTEHAPVGITGAAEWR----IKKES
Burkholderia sp. (WP013592484)   AWANLPAKLLVSAG-------------PIVTVLGGRDSLYSIG-TAYEGGQMIFGTRSRVNGALGAQGFVGAALDVGGVAGMAGGWASSSV-----GGYL
Burkholderia sp. (ADN59567)      AWGNLPARLLVSAG-------------PIVTVLGGRDSMYSIG-TAYEGGQMMFGTRSRVNGTLGAQGFVGATLDVAGVAGMAGAWAASSV-----GGYL
R. solanacearum (YP003748288)    ALTLNLARIVKRFKGGF-----ALTPVFDAQASVTRSAAFSIG-TTAHGGDIFIGRQRQIGGQLGGGLTAGYTTPTVANEDNFSASVGVSANVTLFGLEH
R. solanacearum (BAH04968)       TLSTGLQNAGHALAIP-------LSVQADFHASRKRQAVVEFG-RGAHGRDLFIGTDKTVHAAAGCGGAIGYDIQVLANRIRASLGMSVVPIDVERGKRV
R. solanacearum (BAD42384)       TLSTGLQNAGHALAIP-------LSVQADFHASRKRQAVVEFG-RGAHGRDLFIGTDKTVHAAAGCGGAIGYDIQVLANRIRASLGMSVVPIDVERGKRV
R. solanacearum (YP003747289)    EFTHNSPVPAMGLG-------------PGAKALHGRHAFVEIG-SSSYGGEVFIGTDKRSSTGAGLGAYAGLKFGTEDWHLSAGFSAGVAH-----SQDR
R. solanacearum (WP013209172)    AFSHNSLVPALGFG-------------PGAKTLRGRHAFVEIG-SSSYGGEVFIGTDKRSSDGTGMGAYAGLKFGTEDWHLSAGFSAGVTH-----SHDR
R. solanacearum (WP011002069)    ALSSHLGVPHVSVL-------------PDGGYLQGRHAVIDIG-SNQHFGHLFIGTESRKSLYGGLGGYAGWSFG-QDGMANVGVSGGLRR-----SRDW
R. solanacearum (WP003275417)    ALSVNVAMAAAAAT-QV-----LVQPIVDVQASVARSAVFTIG-TSSHGGEIFIGRQRQLAGQVGGGVMIGYVPSTEPGEAAFAARLGVAVNTTLFGLEH
R. solanacearum (WP003274509)    EFTHNSPVPAMGLG-------------PGAKALHGRHAFVEIG-SSSYGGEVFIGTDKRSSTGAGLGAYAGLKFGTEDWHLSAGFSAGVAH-----SQDR
R. solanacearum (EAP71656)       ALTLNLARIVKRLKGGF-----ALTPVFDAQASVARSAAFSIG-TTAHGGDIFIGRQRQIGGQLGGGLTAGYTTPTVADENSVSASVGVSANVTLFGLEH
R. solanacearum (EAP71039)       ALSVNVAMAAAAAT-QV-----LVQPIVDVQASVARSAVFTIG-TSSHGGEIFIGRQRQLAGQVGGGVMIGYVPSTEPGETAFAARLGVAVNTTLFGLEH
R. solanacearum (CBJ39728)       AFSHNSLVPSMGLG-------------PGARELTGRHAFVEIG-SSSYGGEVFIGTDKRSSTGAGVGLYAGFKIGIKNWHLSAGFSAGVAH-----AHDR
R. solanacearum (CBJ35355)       ALTLNLARIVKRLKGGV-----TLTPVLDVQGSVARSAAFNIG-TTAHGGEIFIGRQRQIGGQIGGGITAGYSPPTDPGEDSFSASFGVSANVTLFGLEH
R. solanacearum (CAD17997)       TISTGLQNARQALAVP-------VAIQADFHASHKRQAVVEIA-RGSHGRDLFVGTDKTVLAAAGFGASVGYDFKVLQNRIRAMVGASVVPIDVEKGERV
R. solanacearum (BAH47286)       ALSSHLGVPHVSVL-------------PDGGYLQGRHAVIDIG-SNQHFGHLFIGTESRKSLYGGLGGYAGWSFG-QDGMANVGVSDGLRR-----SRDW
R. solanacearum (BAH47283)       EFSHNSLVPSMGLG-------------PGARELTGRHAFVEIG-SSSYGGEVFIGTDKRSSTGVGAGLYAGFKVGIKNWHLSAGFSAGVAH-----AHDR
R. solanacearum (BAH04967)       TISTGLQNARQALAVP-------VAIQADFHASHKRQAVVEIA-RGSHGRDLFVGTDKTVLAAAGFGASVGYDFKVLQNRIRAMVGASVVPIDVEKGERV
R. solanacearum (BAD42389)       ALTLNLASLVKRFKGGF-----ALTPVFDAQGSVARSAAFNIG-TTAHGGEIFIGRQRQVVGQLGGGLTAGYTTPTDADENSFSAGVGISGNVTLFGLEH
```

```
                                          810       820       830       840       850       860       870       880       890       900
                                  ....|....|....|....|....|....|....|....|....|....|....|....|....|....|....|....|....|....|....|....|
M. rhizoxinica M1 (CBW77121)      ARPRGILVQVARQPKADGSGY-------DDEAMEARMGHIIDTMMSLSPAGATAESRGTREQCWNALAQSLVGAR--DVSVGWIDGTVDEKRHGSSTGPV
Mycetohabitans sp. M2 (MN840555)  ARPRGILVQVARQPKADGSGY-------DDEAMEARMGHIIDTMMSLSPAGATAESRGTREQCWNALAKSLVGAR--DVSVGWIDGTVDEKRHGSSTGPV
Mycetohabitans sp. M3 (MN840550)  ARPRGILVQVARQLKEDGSGY-------DDEAMQASVNHILDTMKSLSPAGAAAESRGTSEQCWNTLAQSLVGAR--DVSVGWIEGTVDEKRHGASTGPL
Mycetohabitans sp. M4 (MN840552)  ARPRGILVQVARQLKEDGSGY-------DDEAMQASMNHILDTMKSLSPAGAAAESRGTSEQCWNTLAQSLVGAR--DVSVGWIEGTVDEKRHGASMGPL
M. endofungorum M5 (MN840553)     ARPRGILVQVARQLKEDGSGY-------DDEVMQARVNHILDTMKSLLPAGAAAKSRGTSEQCWNTLAQSLVGAR--DVSVGWIEGTVDEKRHGASMGPL
Mycetohabitans sp. M6 (MN840556)  ARPRGILVQVARQPKADGSGY-------DDEAMEARMGHIIDTMMSLSPAGATAESRGTREQCWNALAQSLVGAR--DVSVGWIDGTVDEKRHGSSTGPV
Mycetohabitans sp. M7 (MN840551)  ARPRGILVQVARQLKEDGSGY-------DDEAMQASVNHILDTMKSLSPAGAAAESRGTSEQCWNTLAQSLVGAR--DVSVGWIEGTVDEKRHGASTGPL
Mycetohabitans sp. M8 (MN840554)  ARPRGILVQVARQLKDDGSGY-------DDEAMQASVNHILDTMKSLSSAGATAESRGTSEQCWNTLAQSLVGAR--DVSVGWIEGTVDEKRHAASTGPL
B. pseudomallei (ZP04890375)      TREKGAIVRFVPRAGSLGGGE----------AWRGWAQDCLSIIGNSADS----------ADLLERLAEEYVNAG--EIGIGMTEKEENVISSSAAVGAG
B. pseudomallei (ZP02502372)      SREKGAIVRFVPRAGSLGGGE----------AWRGWAQDCLSIIGNSADS----------ADLLERLAEEYVNAG--EIGIGMTEKEENVISSSAAVGAG
B. pseudomallei (ZP02494128)      TREKGAIVRFVPRAGSLGGGE----------AWRGWAQDCLSIIGNSADS----------ADLLERLAEEYVNAG--EIGIGMTEKEENVISSSAAVGAG
X. euvesicatoria (WP011347306)    GIEHGVQIRVPRRGKGQELEQ------------RAQFLAMFEHLLQLAEQSGDDAGQLSERDFLGELLAHHPSIT--VGLIGQAERNTSATESSLSVAAG
X. campestris (AZR30894)          GVEHGVQIRVPRRGKGQELEQ------------RAQFLAMFEHLLQLAEQSGGDTAQLTERDFLAELLAHHPSIT--VGLIGHAERNSSTTESSVSVAAG
X. vasicola (AVQ09097)            GVEHGVQIRVPRRGKGQELEQ------------RAQFLAMFEHLLQLAEQSGGDTAQLTERDFLAELLAHHPSIT--VGLIGHAERNSSTTESSVSVAAG
X. citri (ARR12757)               GIEHGVQIRVPRRGKGQELEQ------------RAQFLAMFEHLLQLAEQSGGDAGQLSERDFLGELLAHHPSIT--VGLIGHAERNATATESSLSVAAG
X. campestris (AKS22319)          SIEHGVQIRVPRRGKGQELEQ------------RAQFLAMFEHLLQLAEQSGGDAGQLTERDFLGELLAHHPSIT--VGLIGHAERNASATESSLSVAAG
X. oryzae (AKO19890)              GIEHGVQIRVPRRGKGQELEQ------------RAQFLAMFEHLLQLAEQSGGDTSQLTERDFLAELLAHHPSIT--VGLIGHAERNSSTTESSVSVAAG
X. oryzae (ACD59124)              GIEHGVQIRVPRRGKGQELEQ------------RAQFLAMFEHLLQLAEQSGGDTAQLTERDFLAELLAHHPSIT--VGLIGHAERNSSTTESSVSVAAG
Burkholderia sp. (WP013592484)    AREQSVVLRARRLAPTPATAA------LEAELWHKDSRQMVNAYWDAAEQ-AQTPEEFMQILAAGIADNERISL--SSQNNNATSITATPLTVGGVARAN
Burkholderia sp. (ADN59567)       AREESVVLRARRLAPTPATAE------LEAELWHKDSQQMVNAYWDAAEQ-AQTPDEFMQILAAGIADNERISL--SSQNNNATTVTATPLTVGGVARAN
R. solanacearum (YP003748288)     TNPVGVRLRFSTQRKEDGSSM------NSD-AMRKQMSDMIDYMFDACG---PAKRHLSPAQLWEDFAVHHFDQ--KNLSVGWADYSSLNSKMGGSVTLT
R. solanacearum (BAH04968)        GVMFRAVRQLDRAQQTVGGYHDWEAVKYDDTATRQALISLSNWLFEQAT--EHRQRPLEREAIWNALALHCGTG--NTISVGWVDQRKRRLRHKLRLSGG
R. solanacearum (BAD42384)        GVMFRAVRQLDRAQQTVGGYHDWEAVKYDDTATRQALISLSNWLFEQAT--EHRQRPLEREAIWNALALHCGTG--NTISVGWVDQRKRRLRHKLRLSGG
R. solanacearum (YP003747289)     SAPAGVIIRTG---LSYGADG------KATSAWRDNVAEVTRFLFQTAAL-GQSTRPVPPERMWEQFAERFFRT--PDISVNWRDQRRSSETVTKHGSAA
R. solanacearum (WP013209172)     SAPAGVIIRTG---LSYDAEG------KPTSAWRDNVADVTRFLFQTAAV-GQSARPVPPDRMWEQFADRFFRT--PDISVNWRDQQRSNHTVTKHGSAA
R. solanacearum (WP011002069)     GGPRGVTIRTR---RSDDEQP------GRPDAWRTTMLEVLHATRSAGPN-SDAPR--NAREMWGSLARRFWND--PACSINWTDSRSTTSATSGSASAT
R. solanacearum (WP003275417)     NHPVGVRLRFARQRNASGNDM------VDKGVMRRQMETMVDYLFDACG---PAKRHLSPAALWEDFANHHFDQ--ENLSVAWQDYSALSSRMGVSVTGS
R. solanacearum (WP003274509)     SAPAGVIIRTG---LSYGADG------KATSAWRDNVAEVTRFLFQTAAL-GQATRPVPPERMWEQFAERFFRT--PDISVNWRDQRRSSETVTKHGSGA
R. solanacearum (EAP71656)        TNPVGVRLRFSTQRKEDGSGM------NSD-AMRKQMSDMIDYMFDACG---PAKRHLSPAQLWEDFAVHHFDQ--KNLSVGWADYSTLNSKMGGSVTLT
R. solanacearum (EAP71039)        NHPVGVRLRFARQRNASGNDM------VDKGVMRRQMETMVDYLFDACG---PAKRHLSPAALWEDFANHHFDQ--ENLSVAWQDYSALSSRMGVSVTGS
R. solanacearum (CBJ39728)        SAPVGVIVRTG---LTYGTDG------KATNAWRDNVADVTRFLFQSAAE-GQAARPVPPDRMWEQFSARFFRT--PDISVNWRDQRRSSHTVTKHGSGA
R. solanacearum (CBJ35355)        TNPTGVRLRFTTQRKEDGTAM------NSD-AMRKQMSDMVDYMFDACG---PAKRHLSPSELWEDFAVHHFDQ--KHLSVGWEDYSSLNSKMGGAITAT
R. solanacearum (CAD17997)        GVMYRAVRQVT---QAAGPHADWDAVKYDDAATREAMIALSNWLFQQAT--EHRERPPGADAVWNALAQHCSGAGADAISVGWVDQRKHRLRHKLRVGGG
R. solanacearum (BAH47286)        GGPRGVTIRTR---RSDDEQP------GRPDAWRTTMLEVLHAARSAGPN-SDAPR--NAREMWGSLARRFWND--PACSINWTDSRSTTSATSGSASAT
R. solanacearum (BAH47283)        SAPVGVIVRTG---LTYGADG------KATSAWRDNVADVTRFLFQTAAE-GQAARPVPPDRMWEQFSARFFRT--PDISVNWRDQRRSSHTVTKHGSGA
R. solanacearum (BAH04967)        GVMYRAVRQVT---QAAGPHADWDAVKYDDAATREAMIALSNWLFQQAT--EHRERPPGADAVWNALAQHCSGAGADAISVGWVDQRKHRLRHKLRVGGG
R. solanacearum (BAD42389)        TNPTGVRLRFTTQRKDDGSAM------NSD-AMRKQMSDMVDYMFEACG---PDKRHLSPSALWEDFAVHHFDQ—KNLSVAWEDYASVNSKMGASVTLT
```

```
                                        910       920       930       940       950       960       970       980       990       1000
                                    ....|....|....|....|....|....|....|....|....|....|....|....|....|....|....|....|....|....|....|....|
M. rhizoxinica M1 (CBW77121)        ASAGVPIGLPHPFGMSTSVSVPVSGKHVHN-ISTSRSDSGRMSTEKRHAERSIEVKVEVRVGASVSATLSSNPKSGISTG-------------------P
Mycetohabitans sp. M2 (MN840555)    VSAGVPIGLPHPFGMSTSVSVPVSGKHVNN-ISTSRSDSGRMPTEKRHAERSIEVKVEVKVGASVSATLSSNPKSGVNTG-------------------P
Mycetohabitans sp. M3 (MN840550)    VSTGVPIGLPAPLSTSVSASVPVSGKHVNS-ISTSRNDSGRMSTEKRHTERSIEVKIEAKVGASISATTSSSPKSGVSTG-------------------P
Mycetohabitans sp. M4 (MN840552)    VSTGVPIGLPAPLSTSVSASVPVSGKHVNS-ISTSRSDSGRMSTEKRHTERSIEVKIEAKVGASISATTSSSPKSGVSTG-------------------P
M. endofungorum M5 (MN840553)       VSAGVPLGLPAPFSTSVSASVPVSGKHVNS-ISTSRSDSGRMSTEKRHTERSIEVKIETKVGASISATTSNSPKSGVSTG-------------------P
Mycetohabitans sp. M6 (MN840556)    ASAGVPIGLPHPFGMSTSVSVPVSGKHVHN-ISTSRSDSGRMSTEKRHAERSIEVKVEVRVGASVSATLSSNPKSGISTG-------------------P
Mycetohabitans sp. M7 (MN840551)    VSTGVPIGLPAPLSTSVSASVPVSGKHVNS-ISTSRNDSGRMSTEKRHTERSIEVKIEAKVGASISATTSSSPKSGVSTG-------------------P
Mycetohabitans sp. M8 (MN840554)    VSVGIPNVLPAPLGASVSASVPVSGKHVNS-ISTSRSDSGHMSTEKRHAERSIEVKVEAKVGASVSAATSSSPKSSVSTG-------------------P
B. pseudomallei (ZP04890375)        VKASSSGG---DFRGGAGVSASIGVKREWRRHKAVRELDNKMGNHKFDQSSSTSVTWNAGLNASESYQLNPTPDFSTSAS------------------AS
B. pseudomallei (ZP02502372)        VKASSSGG---DFRGGAGVSASIGVKREWRRHKAVRELDNKMGNHKFDQSSSTSVTWNAGLNASESYQLNPTPDFGTSAS------------------AS
B. pseudomallei (ZP02494128)        VKASSSGG---DFRGGAGVSASIGVKREWRRHKAVRELDNKMGNHKFDQSSSTSVTWNAGLNASESYQLNPTPDFSTSAS------------------AS
X. euvesicatoria (WP011347306)      VRVGNMDGQPRRATLGASLGVKARRETSRS----QTPIEGYMTMVLKDSSAQSRVEIAGRASASVVARQWTDPGTGNAPP-----------TPVARLSMA
X. campestris (AZR30894)            VRVGNMDGRPRRATLGASLGIKARRETSRT----QTPIEGYMTMVLKDSSAQSRVEIAGRASASLVAQQWTQPSTGNAPP-----------NPIARLSMA
X. vasicola (AVQ09097)              VRVGNMDGRPRRATLGASLGLKARRETSRT----QTPIEGYMTMVLKDSSAQSRVEIAGRASASLVAQQWTQPSTGNAPP-----------NPIARLSMA
X. citri (ARR12757)                 VRVGNMDGRPRRATLGASLGLKARRETSRS----QTPIEGYMTMVLKDSSAQSRVEIAGRASASLVAQQWTHPSTGNAPP-----------TPVARLSMA
X. campestris (AKS22319)            VRVGNMDGKPRRATLGASLGLKARRETSRS----QTPIEGYMTMVLKDSSAQSRVELAGRASASVVAQQWTHPSIGNAPP-----------TPVARLSMA
X. oryzae (AKO19890)                VRVGNMDGRPRRATLGASLGLKARRETSRS----QTPIEGYMTMVLKDSTAQSRVDIAGRASASLVAQQWTQPSAGNAPP-----------SPVARLSMA
X. oryzae (ACD59124)                VRVGNMDGRPRRATLGASLGLKARRETSRS----QTPIEGYMTMVLKDSTAQSRVDIAGRASASLVAQQWTQPSAGNAPP-----------SPVARLSMA
Burkholderia sp. (WP013592484)      VPGASSS-----ERVGGYAGVSSG-VTLYS-AADLKESGALASTIGATAVQGSLTASVG--AQFAPRAFALGDGTGEIGS---------------VSPGA
Burkholderia sp. (ADN59567)         VPGANSP-----ERVGAYAGVSSG-VTLYS-AADLKESGALASTIGATAVQGTLSASAG--TQFAPRALMLGDGAGEVGS---------------VSPGA
R. solanacearum (YP003748288)       ARAGVTTEDGQAIRAGGSIGYGVT-WNPFT-MGSRKEKSGTAPILREDRGSAHIQTLTATASGQLPAVPLPVEPDGAANG---------------LGVPG
R. solanacearum (BAH04968)          VQARISSK-GAPIRVGPSARLTAE-ATSRD-ATATRETAGIMRVEQSVQGHGGHLTLRAGLTYSAGKMFYTSAAPDAATG----SDATGGRHRITQGFNG
R. solanacearum (BAD42384)          VQARISSK-GAPICVGPSARLTAE-ATSRD-ATATRETAGIMRVEQSVQGHGGHLTLRAGLTYSAGKMFYTSAAPDAATG----SDATGGRHRITQGFNG
R. solanacearum (YP003747289)       MRVAAGP-----VRLGPAFSVGHD-HVLAS-KNDRVDTNGWLRGVERARARASNVHVGASLAALAPAVGHFSNRSGFPDT---------------ITLPS
R. solanacearum (WP013209172)       VRVAAGP-----VRVGPAFAVGHE-RLLAS-KNDRIDDNGWLRGVERARAQSSSVHVSASLAAVAPAIGHFSNHSGFPDS---------------ITLPS
R. solanacearum (WP011002069)       ARVGTAD-----TKWGPALGATLR-HVARA-AHRQWDKTGNHAIDVSTHNSGRATAVAATLVEALPGIP-VPNGSGHLAA---------------LSFPT
R. solanacearum (WP003275417)       ARLGVKTDGGETVRTGGLFGYAFN-WSPFT-RGKRQEASGRFPVMRNERGVMYSHTVTVATGTQLPSVPLPDSHEGVSDS---------------LGLPN
R. solanacearum (WP003274509)       VRVAAGP-----VRLGPAFSVGHD-QVLAS-KNDRVDTNGWLRGVERARARASNVHVGASLAALAPAVGHFSNRSGFPDS---------------ITLPS
R. solanacearum (EAP71656)          ARAGVTTEDGQAIRAGGSIGYGVT-WNPFT-MGSRKEKSGTAPILREDRGSAHIHTLTATASGQLPAVPLPAQPDGATNS---------------LGVPA
R. solanacearum (EAP71039)          ARLGVKTDGGETVRTGGLFGYAFN-WSPFT-RGKRQEASGRFPVMRNERGVMYSHTVTVATGTQLPSVPLPDSHEGVSDS---------------LGLPN
R. solanacearum (CBJ39728)          VRVAAGQ-----VRVGPAFSVGHD-RVLAS-KNDRVDTNGWLRGVERARARASNVHVSGSLAAVAQGVGHFSNRSGFPES---------------VTLPS
R. solanacearum (CBJ35355)          ARAGVKTADGQTVRGGGSIGYGFT-WNPFT-MGERKEKSGTAPILREDRGSAHIHTVTVAASGQLPGVPLPDSLDGAANS---------------LGVPS
R. solanacearum (CAD17997)          VNVRVTAP-AAPVRVGVGVRLTGE-ITSRD-TTAMRETTGIMRAEQYSQGHGGHMTVRAGVNYAVGKRFYTSPAQDAAAAGPTASVPSGGRRSITQGFNG
R. solanacearum (BAH47286)          ARVGTAD-----TKWGPALGATLR-HVARA-AHRQWDKTGNHAIDVSTHNSGRATAVAATLVEALPGIP-VPNGSGHLAA---------------LSFPT
R. solanacearum (BAH47283)          VRVAAGA-----VRVGPAFSVGHD-QVLAS-KNDRVDTNGWLRGVERARARASNVHVSGSLAAVAQGVGHFSNRSGFPES---------------VTLPS
R. solanacearum (BAH04967)          VNVRVTAP-AAPVRVGVGVRLTGE-ITSRD-TTAMRETTGIMRAEQYSQGHGGHMTVRAGVNYAVGKRFYTSPAQDAAAAGPTASVPSGERRSITQGFNG
R. solanacearum (BAD42389)          ARAGVTTQDGQTVRAGGSVGYGFT-WNPFT-IGQRQEKSGIAPILREDRGSAHLHAVTVAASVQLPAGPLPDTPDGAANS---------------LGVPS
```

```
                                             1010      1020      1030      1040      1050      1060      1070      1080      1090      1100
                                         ....|....|....|....|....|....|....|....|....|....|....|....|....|....|....|....|....|....|....|....|
M. rhizoxinica M1 (CBW77121)             SSKRLWDKQLYQAKVSLSRTLQSYQGKIEPRACFLDVDFLDATAYEYATRQLLDHNGDRDDAGIKAVRTYLEQKRAQSAKPLADQAASAVKNVLELGNGT
Mycetohabitans sp. M2 (MN840555)         SSKRLWDKQLYQAKVSLSRTLQSYQGKIEPRACFLDVDFPDATAYEYATRQLLDHNGDRDDAGIKAVRTYLEQKRAQSAKPLADQAASAVKNVLELGNGT
Mycetohabitans sp. M3 (MN840550)         TSKRLWDKQIYQVKVSLSRTLQTHHGKIDPRACFLDVDFPDATAYEYATRQLLNHDGDHDDTGIKAVGTYLEQKRAQSTEPLANQVASAVKNVLELGNGT
Mycetohabitans sp. M4 (MN840552)         TSKRLWDKQIYQVKVSLSRKLQTHHGKIDPRACFLDVDFPDATAYEYATRQLLDHDGDHDDTGIKAVGTYLEQKRAQSTEPLANQVASAVKNVLELGNGT
M. endofungorum M5 (MN840553)            TSKRLWDKQIYQVKVSLSRTLQTHHGKIDPRACFLDVDFPDATAYEYATRQLLDHDGAHDDAGIKAVGTYLEQKRAQSAEPLANQVASAVKNVLELGNGT
Mycetohabitans sp. M6 (MN840556)         SSKRLWDKQLYQAKVSLSRTLQSYQGKIEPRACFLDVDFLDATAYEYATRQLLDHNGDRDDAGIKAVRTYLEQKRAQSAKPLADQAASAVKNVLELGNGT
Mycetohabitans sp. M7 (MN840551)         TSKRLWDKQIYQVKVSLSRTLQTHHGKIDPRACFLDVDFPDATAYEYATRQLLNHDGDHDDTGIKAVGTYLEQKRAQSTEPLANQVASAVKNVLELGNGT
Mycetohabitans sp. M8 (MN840554)         TSKRLWDKQIYQVKVSLSRTVQSYQGKIDPRACFLDVNFPDATTYEYATRQLLDHDGDHDDTGIKAVGTYLKQKRTQSAKLLTDQVASAVKNMLELGNGT
B. pseudomallei (ZP04890375)             AQAFSVGGTLFSAKEGASVAIRLRDGMVDSELAILERTRNRGRRGVELIARDYPLWVKAMGKTDAEGEAALNR-----------FFASELGRGVNRPLRP
B. pseudomallei (ZP02502372)             AQAFSIGGTLFSVKEGTSVAIRLRDGMVDAELAILDRTRNGGRRAVDLIARDYPIWVKAMGKTDAEGEAALNR-----------FFASELGRGVNRPLRP
B. pseudomallei (ZP02494128)             AQAFSIGGTLFSVKEGTSVAIRLRDGMVDAELAILDRTRNGGRRAVDLIARDYPIWVKAMGKTDAEGEAALNR-----------FFASELGRGVNRPLRP
X. euvesicatoria (WP011347306)           TLDLGYSRELRSAGSTTFSTLWMFNNEIDPVRTDRGFEFFNFASFEREVKRNWQLWTHYGISKLQGKVDDQR------LYMVAERQLQDFIDRVRLHMQD
X. campestris (AZR30894)                 ALDLGYSREVHSAGSNTFSTLWMFNNEIDPVRTDRGFEFFDVASFEREVKRNWQLWTHYGISKLQGKVDDQR------LYMVAERQLQDFVDRVRIHMQD
X. vasicola (AVQ09097)                   ALDLGYSREVHSAGSNTFSTLWMFNNEIDPVRTDRGFEFFDVASFEREVKRNWQLWTHYGISKLQGKVDDQR------LYMVAERQLQDFVDRVRIHMQD
X. citri (ARR12757)                      TLDLGYSRELRSAGSNTFSTLWMFNNEIDPVRTDRGFEFFNFASFEREVKRNWQLWTHYGISKLQGKVDEQR------LYMVAERQLQDFIDRVRLHMQD
X. campestris (AKS22319)                 TLDLGYSRELHSAGSNTFSTLWMFNNEIDPVRTDRGFEFFDFASFEREVKRNWQLWTHYGISKLQGKVDDQR------VYMVAERQLQDFVDRVRLHMQD
X. oryzae (AKO19890)                     ALDLGYSRELHLAGSNTFSTLWMFNNEIDPVRTDRGFEFFNFASFEREVKRNWQLWTNYGISKLQGKVDDHR------LYMVAERQLQDFIDRVRIHMQD
X. oryzae (ACD59124)                     SLDLGYSRELHLAGSNTFSTLWMFNNEIDPVRTDRGFEFFNFASFEREVKRNWQLWTHYGISKLQGKVDDQR------LYMVAERQLQDFIDRVRIHMQD
Burkholderia sp. (WP013592484)           KTLGGAAVELTPFGRSSFVFAMRDNRGFVAEYTMQDRMFRNADDWARSVRAN-------PAWAHGIGA----------------ERLQQVIEQTQQ--ES
Burkholderia sp. (ADN59567)              KTLASAAVEATPFGRSSFVFAMRDNRGFVAEYTMQDRMFRNADDWARSVRAN-------PAWLQGIGA----------------QRLQQVIEQTQL--ES
R. solanacearum (YP003748288)            VPVASATYMLGNGGFNANIRTLVERGRLSEAFTYRDLSERNIDDFLKFANDPARRKEWEAMCGAEQGAYAEHG--AGGPDLGKKRLDDFLGKIQEM--AR
R. solanacearum (BAH04968)               GTPLSWSKQFAELGHTVEVRTIHLNGRLADRVSYSDKRYKSGTEFLALIRTRQRVWIDMLSRKPGMTREAAE------------QEFKVFCDMVER--HS
R. solanacearum (BAD42384)               GTPLSWSKQFAELGHTVEVRTIHLNGRLADRVSYSDKRYKSGTEFLALIRTRQRVWIDMLSRKPGMTREAAE------------QEFKVFCDMVER--HS
R. solanacearum (YP003747289)            TPIVGLSANILPSGTSVTLRRVDEHGRLNPRFIRRLVEFIDPSAFLSHMQAR----MPRIARTDASRE----------------KVGAFMSAMKDM--GT
R. solanacearum (WP013209172)            APIVGVAANIMPSGTSVTLRRVDEHGRLNPRFIRRLVEFIDPSAFLSHMQAR----LPRIAHTDASRA----------------KVGAFMSAMDDM--GT
R. solanacearum (WP011002069)            QPYVGIGTTLFTTQTNAALRIGRDSGRIIPKHTFRDTEFGTFKAFKQFVDTHRSEWLTALGGTDDARR----------------RLDEMVATVKAR--AT
R. solanacearum (WP003275417)            VPIHSATYMLGEGGFNAIVRTLMEHGRHSETFTYRDLSERSIHDFLKIANEPGRRKAWEALCAAEQGGDAARG---------KERLDDFLDKVREM--AR
R. solanacearum (WP003274509)            TPIVGLSANILPSGTSVTLRRVDEHGRLNPRFIRRLVEFIDPSAFLSHMQAR----MPRIARTDASRE----------------KVGAFMSAMKDM--GT
R. solanacearum (EAP71656)               VPVASATYMVGNGGFNANIRTLVERGRLSEAFTYRDLSERSIDDFLKFANDPARRKEWEAVCGAEQGAYAEHG--EGGPDLGKKRLDDFLDKIREM--AR
R. solanacearum (EAP71039)               VPIHSATYMLGEGGFNAIVRTLMEHGRHSETFTYRDLSERSIHDFLKIANEPGRRKAWEALCAAEQGGDAARG---------KERLDDFLDKVREM--AR
R. solanacearum (CBJ39728)               TPIVGLSANILPSGTSVTLRRVDEHGRLNPRFIRRLVEFINPSAFLSHMQAR----LPQIAHSDASRA----------------KVGAFMSAMKDM--GT
R. solanacearum (CBJ35355)               VPVASATYMLGNGGFNATVRTLLERGRLSEAFTYRDLSERNIGDFLKFANDPARRKEWEALCSAEQGAYADTGHGEAGPDLGKKRLDDFLSKIQEM--AR
R. solanacearum (CAD17997)               GTPLTYTKQFAEHSHTAKLKTVHLDGRLADRVCYFDKEYKSAKEYLALIRSAQDTWIDMLSRKPGGTVEKAR------------KEFEDYCATVEQ--HT
R. solanacearum (BAH47286)               QPYVGIGTTLFATQTNAALRIGRDSGRIIPKHTFRDTEFGTFKAFKQFVDTHRSEWLTALGGTDDARR----------------RLDEMVATVKAR--AT
R. solanacearum (BAH47283)               VPIVGASANILPSGTSVTLRRVDEHGRLNPRFIRRLVEFIDPSAFLSHMQAR----LPQIAHSDASRA----------------KVGAFMSAMRDM--GT
R. solanacearum (BAH04967)               GTPLTYTKQFAEHSHTAKLRTVHLDGRLADRVCYFDKEYKSAKEYLALIRSAQDTWIDMLSRKPGCTVEKAR------------KEFEDYCATIEQ--HT
R. solanacearum (BAD42389)               VPIASATYMLGNGGFNATIRTLMERGRLSEAFTYRDLSERNINDFIKFANDPARRKEWEALCSAEQGAYAAHGKADAGP--GKKRLDDFLDKIQEM—AR
```

	1110 1120 1130
	....|....|....|....|....|....|...
M. rhizoxinica M1 (CBW77121)	RQTDSHIHRYALTDPAAEQINLLQAHFEQHYLR
***Mycetohabitans* sp. M2 (MN840555)**	**RQTDSHIHRYALTDPAAEQINLLQAHFEQHYLR**
***Mycetohabitans* sp. M3 (MN840550)**	**RQMHSHIHRYALTDPAAEQINLLQAHFEQHYLR**
***Mycetohabitans* sp. M4 (MN840552)**	**RQMHSHIHRYALTDPAAEQINLLQAHFEQHYLR**
***M. endofungorum* M5 (MN840553)**	**RQTDSHIHRYALTDPAAEQINLLQAHFEQHYLR**
***Mycetohabitans* sp. M6 (MN840556)**	**RQTDSHIHRYALTDPAAEQINLLQAHFEQHYLR**
***Mycetohabitans* sp. M7 (MN840551)**	**RQMHSHIHRYALTDPAAEQINLLQAHFEQHYLR**
***Mycetohabitans* sp. M8 (MN840554)**	**RQTDSHIHRYALTDPAAEQINLLQAHFEQHYLR**
B. pseudomallei (ZP04890375)	EVPETYVASYRMTKEAASALNHYRAQIDLEQML
B. pseudomallei (ZP02502372)	EVPETYVASYRMTKEAASALNHYRAQIDLEQML
B. pseudomallei (ZP02494128)	EVPETYVASYRMTKEAASALNHYRAQIDLEQML
X. euvesicatoria (WP011347306)	NKFASLIVDWVLQAEAAPRLDALRAQAQLLRVA
X. campestris (AZR30894)	NKFASLIVDWVLQAEAAPRLDALRAQSQLLRVA
X. vasicola (AVQ09097)	NKFASLIVDWVLQAEAAPRLDALRAQSQLLRVA
X. citri (ARR12757)	NKFASLIVDWVLQAEAAPHLDALRAQAQLLRVA
X. campestris (AKS22319)	NKFASMIVDWVLQAEAAPRLDALRAQAQLLRVA
X. oryzae (AKO19890)	NKFASLIVDWVLQAEAAPRLDALRAQAQLLRVA
X. oryzae (ACD59124)	NKFASLIVDWVLQAEAAPRLDALRAQAQLLRVA
Burkholderia sp. (WP013592484)	SGEFSFGERWAIREERVPRLNQYLTLSNQLRAR
Burkholderia sp. (ADN59567)	SGEFSFGERWVIADEYVPRLNQYLTLSNQLRAR
R. solanacearum (YP003748288)	P-NQAHYMRWRLGEQERLAMDDYMAAARMAERG
R. solanacearum (BAH04968)	GPNVSYLYRERLKPAIAREIEGQLDLADMHRAL
R. solanacearum (BAD42384)	GPNVSYLYRERLKPAIAREIEGQLDLADMHRAL
R. solanacearum (YP003747289)	QGNLAYGESWKIRPEVTEVLNAYTDEIQLILKC
R. solanacearum (WP013209172)	QGNLAYGESWKIRPEVTEVLNAYTDEIQLILKC
R. solanacearum (WP011002069)	AGNLIMGERMHMTDEAARRL-------------
R. solanacearum (WP003275417)	P-NQAYYMRWRLGTEERLAMDDYLGVAKIAERG
R. solanacearum (WP003274509)	QGNLAYGESWKIRPEVTEVLNAYTDEILLILKC
R. solanacearum (EAP71656)	P-NQAHYMRWRLGEQERLAIDDYMAAARMAERG
R. solanacearum (EAP71039)	P-NQAYYMRWRLGTEERLAMDDYLGVAKIAERG
R. solanacearum (CBJ39728)	QGNLAYGESWKIRPEVTEVLNAYTDEIQLMLKC
R. solanacearum (CBJ35355)	P-NQAHYMRWRLGEQERLAMDDYMAAARMAERG
R. solanacearum (CAD17997)	GPHIRYLLRERLRPAAAREVEGHLDLANLHRAT
R. solanacearum (BAH47286)	AGNLIMGERMHMTDEAARRL-------------
R. solanacearum (BAH47283)	QGNLAYGESWKIRPEVTEVLNAYTDEIQLMLKC
R. solanacearum (BAH04967)	GPHIRYLLRERLRPAAAREVEGHLDLANLHRAT
R. solanacearum (BAD42389)	P-NQAHYMRWRLGEQERLAMDDYMAADRG

Fig. S1. Alignment of predicted AWR proteins from endofungal *Mycetohabitans* species, plant pathogenic *R. solanaceraum*, and plant-associated *Burkholderia* spp. modified from Sole *et al*. Sequences identified in this study are highlighted in bold (Table S3). GenBank accession numbers are indicated in brackets.

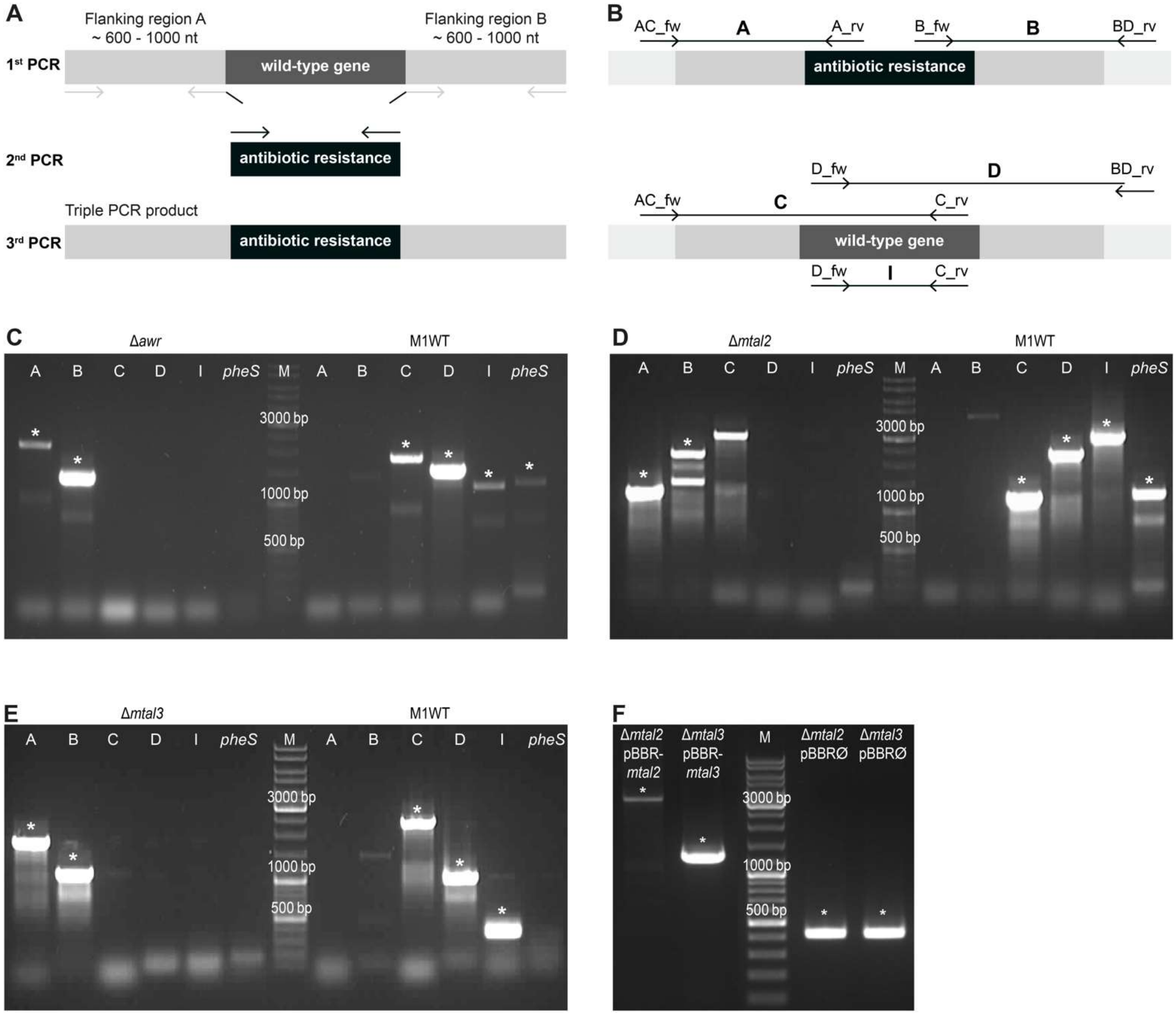

Fig. S2. Confirmation of *M. rhizoxinica* AWR and MTAL mutant strains and their complementation by PCR. (A) Schematic representation of the construction of mutant vector. **(B)** Schematic representation of the confirmations of successful gene inactivation. The PCR products of the mutant strain correspond to amplicons A and B, whereas products C and D are amplified from the wild type. Absence of contaminating *M. rhizoxinica* wild type (M1WT) in the mutant strains was confirmed by lack of amplification of an internal fragment (amplicon I) from the wild-type gene of interest. The removal of the mutant vector was confirmed by amplification of the counter-selection marker gene *pheS* (amplicons *pheS*). **(C)** The *awr* gene was deleted in *M. rhizoxinica* M1WT to generate *M. rhizoxinica* Δ*awr*::Kan^{R} (*M. rhizoxinica* Δ*awr*). PCR products were obtained from genomic wild-type and mutant DNA using control primers listed in Table S4B. Bands corresponding to the expected sizes are indicated by asterisks (*). **(D)** The *mtal2* gene was deleted in *M. rhizoxinica* HKI-454 to generate *M. rhizoxinica* Δ*mtal2*::Kan^{R} (*M. rhizoxinica* Δ*mtal2*). PCR products were obtained from genomic wild-type and mutant DNA using control primers listed in Table S4B. Bands corresponding to the expected sizes are indicated by asterisks (*). **(E)** The *mtal3* gene was deleted *M. rhizoxinica* HKI-454 to generate *M. rhizoxinica* Δ*mtal3*::Kan^{R} (*M. rhizoxinica* Δ*mtal3*). PCR products were obtained from genomic wild-type and mutant DNA using control primers listed in Table S4B. Bands corresponding to the expected sizes are indicated by asterisks (*). **(F)** Colony PCRs were performed to confirm the presence of the relevant complementation plasmids in the strains *M. rhizoxinica* Δ*mtal2* pBBR-*mtal2* and *M. rhizoxinica* Δ*mtal3* pBBR-*mtal3*. *M. rhizoxinica mtal* mutant strains were also transformed with an empty control plasmid to generate the negative control strains *M. rhizoxinica* Δ*mtal2* pBBRØ and *M. rhizoxinica* Δ*mtal3* pBBRØ. PCR products were amplified using control primers listed in Table S4C. Bands corresponding to the expected sizes are indicated by asterisks (*).

```
                                         10        20        30        40        50        60        70        80        90       100
                                 ....|....|....|....|....|....|....|....|....|....|....|....|....|....|....|....|....|....|....|....|
M. rhizoxinica M1 (E5AV36)       FSQSDIVKIAGNIGGAQALQAVLDLESMLGKRGFSRDDIAKMAGNIGGAQTLQAVLDLESAFRER--GFSQADIVKIAGNNGGAQALYSVLD--VEPTLG
Mycetohabitans sp. M2 (MN891944) FSQSDIVKIAGNIGGAQALQAVLDLESMLGKRGFSRDDIAKMAGNIGGAQTLQAVLDLESAFRER--GFSQADIVKIAGNNGGAQALYSVLD--VEPTLG
Mycetohabitans sp. M4 (MN840538) --------------------------------LSPLERSKIEKH--YGGAATLAFISNQHDELAQ--VLSRTDILKIASYDCAAQALQAVLD--CGPMLG
M. endofungorum M5 (MN891945)    FSQSDIVKIAGNIGGAQALQAVLDLESMLGKRGFSRDDIAKMAGNIGGAQTLQAVLDLESAFRER--GFSQADIVKIAGNNGGAQALYSVLD--VEPTLG
Mycetohabitans sp. M6 (MN840537) FSQSDIVKIAGNIGGAQALQAVLDLESMLGKRGFSRDDIAKMAGNIGGAQTLQAVLDLESAFRER--GFSQADIVKIAGNNGGAQALYSVLD--VEPTLG
Mycetohabitans sp. M7 (MN840539) ----------------------------------------------------------------------------------------------------
Mycetohabitans sp. M7 (MN840540) ----------------------------------------------------------------------------------------------------
Mycetohabitans sp. M8 (MN840541) ----------------------------------------------------------------------------------------------------
M. rhizoxinica M1 (E5AW45)       FSRADIVRIAGNGGGAQALYSVLDVEPTLGKRGFSQVDVVKIAG--GGAQALHTVLEIGPTLGER--GFSRGDIVTIAGNNGGAQALQAVLE--LEPTLR
Mycetohabitans sp. M2 (MN840542) FSRADIVRIAGNGGGAQALYSVLDVEPTLGKRGFSQVDVVKIAG--GGAQALHTVLEIGPTLGER--GFSRGDIVTIAGNNGGAQALQAVLE--LEPTLR
Mycetohabitans sp. M3 (MN840545) ----------------------------------------------------------------------------------------------------
Mycetohabitans sp. M6 (MN840543) FSRADIVRIAGNGGGAQALYSVLDVEPTLGKRGFSQVDVVKIAG--GGAQALHTVLEIGPTLGER--GFSRGDIVTIAGNNGGAQALQAVLE--LEPTLR
Mycetohabitans sp. M7 (MN840544) ----------------------------------------------------------------------------------------------------
M. rhizoxinica M1 (E5AW43)       ----------------------------------------------------------------------------------------------------
Mycetohabitans sp. M2 (MN840547) ----------------------------------------------------------------------------------------------------
Mycetohabitans sp. M4 (MN840549) ----------------------------------------------------------------------------------------------------
Mycetohabitans sp. M6 (MN840546) ----------------------------------------------------------------------------------------------------
Mycetohabitans sp. M7 (MN840548) ----------------------------------------------------------------------------------------------------
AvrXa10 X. oryzae (Q56830)       ---------------------------------LTPDQVVAIASNIGGNQALETVQRLLPVLCQA-HGLTPDQVVAIASHGGGKQALETVQR-LLPVLCQ
AvrBs3 X. euvesicatoria (P14727) ---------------------------------LTPEQVVAIASHDGGKQALETVQRLLPVLCQA-HGLTPQQVVAIASNGGGKQALETVQR-LLPVLCQ
Brg11 R. solanacearum (Q8XYE3)    ---------------------------------LTPQQVVAIASNTGGKRALEAVCVQLPVLRAAPYRLSTEQVVAIASNKGGKQALEAVKAHLLDLLGA

                                        110       120       130       140       150       160       170       180       190       200
                                 ....|....|....|....|....|....|....|....|....|....|....|....|....|....|....|....|....|....|....|
M. rhizoxinica M1 (E5AV36)       KRGFSRADIVKIAGNTGGAQALHTVLDLEPALGKRGFS-------------------------------RIDIVKIAANNGGAQALHAVLD--LGPTLRE
Mycetohabitans sp. M2 (MN891944) KRGFSRADIVKIAGNTGGAQALHTVLDLEPALGKRGFS-------------------------------RIDIVKIAANNGGAQALHAVLD--LGPTLRE
Mycetohabitans sp. M4 (MN840538) KRGFSRGDIVKIAGNDGGAQALYSVLDVEPPLRERGFS-------------------------------RADIVKIAGNIGGAQALQAVLE--LEPTLRE
M. endofungorum M5 (MN891945)    KRGFSRADIVKIAGNTGGAQALHTVLDLEPALGKRGFS-------------------------------RIDIVKIAANNGGAQALHAVLD--LGPTLRE
Mycetohabitans sp. M6 (MN840537) KRGFSRADIVKIAGNTGGAQALHTVLDLEPALGKRGFS-------------------------------RIDIVKIAANNGGAQALHAVLD--LGPTLRE
Mycetohabitans sp. M7 (MN840539) ----------------------------------------------------------------------------------------VLE--LESTLRE
Mycetohabitans sp. M7 (MN840540) ----------------------------------------------------------------------------------------VLE--LESTLRE
Mycetohabitans sp. M8 (MN840541) ----------------------------------------------------------------------------------------------------
M. rhizoxinica M1 (E5AW45)       ERGFNQADIVKIAGNGGGAQALQAVLDVEPALGKRGFSRVDIAKIAGGGAQALQAVLGLEPTLRKRGFHPTDIIKIAGNNGGAQALQAVLD--LELMLRE
Mycetohabitans sp. M2 (MN840542) ERGFNQADIVKIAGNGGGAQALQAVLDVEPALGKRGFSRVDIAKIAGGGAQALQAVLGLEPTLRKRGFHPTDIIKIAGNNGGAQALQAVLD--LELMLRE
Mycetohabitans sp. M3 (MN840545) ------------------MSAMFMPQEGKQSANGLNLS-------------------------------PLERIKIEKHYGGSATLAFISN--QHDELAQ
Mycetohabitans sp. M6 (MN840543) ERGFNQADIVKIAGNGGGAQALQAVLDVEPALGKRGFSRVDIAKIAGGGAQALQAVLGLEPTLRKRGFHPTDIIKIAGNNGGAQALQAVLD--LELMLRE
Mycetohabitans sp. M7 (MN840544) ------------------MPVTSVYQKDKPFGARLNLS-------------------------------PLERIKIEKHYGGSATLEFISN--QHDKLAQ
M. rhizoxinica M1 (E5AW43)       ----------------------------------------------------------------------------------------------------
Mycetohabitans sp. M2 (MN840547) ----------------------------------------------------------------------------------------------------
Mycetohabitans sp. M4 (MN840549) ----------------------------------------------------------------------------------------------------
Mycetohabitans sp. M6 (MN840546) ----------------------------------------------------------------------------------------------------
Mycetohabitans sp. M7 (MN840548) ----------------------------------------------------------------------------------------------------
AvrXa10 X. oryzae (Q56830)       AHGLTPDQVVAIASNIGGKQALATVQR-LLPVLCQDHG-----------------------------LTPDQVVAIASHGGGKQALETVQR-LLPVLCQD
AvrBs3 X. euvesicatoria (P14727) AHGLTPQQVVAIASNSGGKQALETVQR-LLPVLCQAHG-----------------------------LTPEQVVAIASNGGGKQALETVQR-LLPVLCQA
Brg11 R. solanacearum (Q8XYE3)    PYVLDTEQVVAIASHNGGKQALEAVKADLLDLRGAPYA-----------------------------LSTEQVVAIASHNGGKQALEAVKADLLELRGAP
```

```
                                        210       220       230       240       250       260       270       280       290       300
                                 ....|....|....|....|....|....|....|....|....|....|....|....|....|....|....|....|....|....|....|....|
M. rhizoxinica M1 (E5AV36)       CGFSQATIAKIAGNIGGAQALQMVLD--LGPALGKRGFSQATIAKIAGN---------------------------------IGGAQALQTVLD--LEPA
Mycetohabitans sp. M2 (MN891944) CGFSQATIAKIAGNIGGAQALQMVLD--LGPALGKRGFSQATIAKIAGN---------------------------------IGGAQALQTVLD--LEPA
Mycetohabitans sp. M4 (MN840538) RGFSQVDIVKIASNDGGAQALYSVLD--VEPTFRERGFSRADIVKIAGNNGGAQALHTVLDVESALSEHGFCRGDTVKIAGNIGGAQALQAVLE--LEPT
M. endofungorum M5 (MN891945)    CGFSQATIAKIAGNIGGAQALQMVLD--LGPALGKRGFSQATIAKIAGN---------------------------------IGGAQALQTVLD--LEPA
Mycetohabitans sp. M6 (MN840537) CGFSQATIAKIAGNIGGAQALQMVLD--LGPALGKRGFSQATIAKIAGN---------------------------------IGGAQALQTVLD--LEPA
Mycetohabitans sp. M7 (MN840539) RSFNRADIVRIAGNGGGAQSLYSVRD--AGPTLGRRGFSRVDIVKIAG-----------------------------------GGAQALHTVLE--LEPT
Mycetohabitans sp. M7 (MN840540) RSFNRADIVRIAGNGGGAQSLYSVRD--AGPTLGKRGFSRVDIVKIAG-----------------------------------GGAQALHTVLE--LEPT
Mycetohabitans sp. M8 (MN840541) ---------------------------------------------MAGNI---------------------------------GGAQALQAVLE--LEPA
M. rhizoxinica M1 (E5AW45)       RGFSQADIVKMASNIGGAQALQAVLN--LEPALCERGFSQPDIVKMAGNSGGAQALQAVLDLELAFRERGFSQADIVKMASNIGGAQALQAVLE--LEPA
Mycetohabitans sp. M2 (MN840542) RGFSQADIVKMASNIGGAQALQAVLN--LEPALCERGFSQPDIVKMAGNSGGAQALQAVLDLELAFRERGFSQADIVKMASNIGGAQALQAVLE--LEPA
Mycetohabitans sp. M3 (MN840545) -VLSRADILKIASYDCAAQALQAVLD--CGPMLGKRGFSRADIVKIAGNG---------------------------------GGAQALQAVLE--LEPT
Mycetohabitans sp. M6 (MN840543) RGFSQADIVKMASNIGGAQALQAVLN--LEPALCERGFSQPDIVKMAGNSGGAQALQAVLDLELAFRERGFSQADIVKMASNIGGAQALQAVLE--LEPA
Mycetohabitans sp. M7 (MN840544) -VLSRADILKIASYDCAAQALQAVLD--CGPMLGKRGFSRADIVKIAGNG---------------------------------GGAQALQAVLE--LEPT
M. rhizoxinica M1 (E5AW43)       -------------------------------------FARADIIKITGNG---------------------------------GGAQALKAVVV--HGPT
Mycetohabitans sp. M2 (MN840547) -------------------------------------FARADIIKITGNG---------------------------------GGAQALKAVVV--HGPT
Mycetohabitans sp. M4 (MN840549) ----------------------------------------------------------------------------------------------------
Mycetohabitans sp. M6 (MN840546) -------------------------------------FARADIIKITGNG---------------------------------GGAQALKAVVV--HGPT
Mycetohabitans sp. M7 (MN840548) ----------------------------------------------------------------------------------------------------
AvrXa10 X. oryzae (Q56830)        HGLTPDQVVAIASNIGGKQALETVQR-LLPVLCQDHGLTPDQVVAIASNI---------------------------------GGKQALETVQR-LLPVL
AvrBs3 X. euvesicatoria (P14727) HGLTPEQVVAIASNIGGKQALETVQA-LLPVLCQAHGLTPEQVVAIASNI---------------------------------GGKQALETVQA-LLPVL
Brg11 R. solanacearum (Q8XYE3)    YALSTEQVVAIASHNGGKQALEAVKAHLLDLRGVPYALSTEQVVAIASHN---------------------------------GGKQALEAVKAQLLDLR

                                        310       320       330       340       350       360       370       380       390       400
                                 ....|....|....|....|....|....|....|....|....|....|....|....|....|....|....|....|....|....|....|....|
M. rhizoxinica M1 (E5AV36)       LCERGFSQATIAKMAGNNGGAQALQ------------------------------------------------------------------TVLDLEPAL
Mycetohabitans sp. M2 (MN891944) LCERGFSQATIAKMAGNNGGAQALQ------------------------------------------------------------------TVLDLEPAL
Mycetohabitans sp. M4 (MN840538) LRERGFSRADILRIAGNGGGAQALQ------------------------------------------------------------------AVLELEPTL
M. endofungorum M5 (MN891945)    LCERGFSQATIAKMAGNNGGAQALQ------------------------------------------------------------------TVLDLEPAL
Mycetohabitans sp. M6 (MN840537) LCERGFSQATIAKMAGNNGGAQALQ------------------------------------------------------------------TVLDLEPAL
Mycetohabitans sp. M7 (MN840539) LRKRGFNPTDIVRMAGNDGGAQALQ------------------------------------------------------------------AVFELEPAF
Mycetohabitans sp. M7 (MN840540) LRKRGFNPTDIVKIAGNDGGAQALQ------------------------------------------------------------------AVLELEPAL
Mycetohabitans sp. M8 (MN840541) FRERGFSQPDIVKMAGNIGGAQALQ------------------------------------------------------------------AVLELEPML
M. rhizoxinica M1 (E5AW45)       LHERGFSQANIVKMAGNSGGAQALQAVLDLELVFRERGFSQPEIVEMAGNIGGAQALHTVLDLELAFRERGVRQADIVKIVGNNGGAQALQAVFELEPTL
Mycetohabitans sp. M2 (MN840542) LHERGFSQANIVKMAGNSGGAQALQAVLDLELVFRERGFSQPEIVEMAGNIGGAQALHTVLDLELAFRERGVRQADIVKIVGNNGGAQALQAVFELEPTL
Mycetohabitans sp. M3 (MN840545) FRERGFSRGDTVKIASNNGGAQALQALQ---------------------------------------------------------------AVLELEPTL
Mycetohabitans sp. M6 (MN840543) LHERGFSQANIVKMAGNSGGAQALQAVLDLELVFRERGFSQPEIVEMAGNIGGAQALHTVLDLELAFRERGVRQADIVKIVGNNGGAQALQAVFELEPTL
Mycetohabitans sp. M7 (MN840544) FRERGFSRGDTVKIAGNGGGAQALP------------------------------------------------------------------AVLELEPTL
M. rhizoxinica M1 (E5AW43)       LNECGFSQADIVRIADNIGGAQALK------------------------------------------------------------------AVLEHGPTL
Mycetohabitans sp. M2 (MN840547) LNECGFSQADIVRIADNIGGAQALK------------------------------------------------------------------AVLEHGPTL
Mycetohabitans sp. M4 (MN840549) --------------------------------------------------------------------------------------------VLDYEQVF
Mycetohabitans sp. M6 (MN840546) LNECGFSQADIVRIADNIGGAQALK------------------------------------------------------------------AVLEHGPTL
Mycetohabitans sp. M7 (MN840548) --------------------------------------------------------------------------------------------VLDYEQVF
AvrXa10 X. oryzae (Q56830)        CQDHGLTPDQVVAIASNNGGKQALETV-----------------------------------------------------------------QRLLPVLC
AvrBs3 X. euvesicatoria (P14727) CQAHGLTPEQVVAIASNIGGKQALETV-----------------------------------------------------------------QALLPVLC
Brg11 R. solanacearum (Q8XYE3)    GAPYALSTAQVVAIASNGGGKQALEGIG----------------------------------------------------------------EQLLKLRT
```

```
                                       410       420       430       440       450       460       470       480       490       500
                                ....|....|....|....|....|....|....|....|....|....|....|....|....|....|....|....|....|....|....|....|
M. rhizoxinica M1 (E5AV36)      RKRDFRQADIIKIAGNDG-------------------------------GAQALQAV--IEHGPTLRQHGFNLADIVKMAGNIGGAQALQAVLDLKPVLD
Mycetohabitans sp. M2 (MN891944)RKRDFRQADIIKIAGNDG-------------------------------GAQALQAV--IEHGPTLRQHGFNLANIVKMAGNIGGAQALQAVLDLKPVLD
Mycetohabitans sp. M4 (MN840538)RERSFNRADIVRIAGNGGGAQALYSVRDAGPTLGKRGFSQVDIVKIAGGGAQALHTV--LELEPTLRKRGFNPTDIVRMAGNDGGAQALQAVLELEPAFR
M. endofungorum M5 (MN891945)   RKRDFRQADIIKIAGNDG-------------------------------GAQALQAV--IEHGPTLRQHGFNLADIVKMAGNIGGAQALQAVLDLKPVLD
Mycetohabitans sp. M6 (MN840537)RKRDFRQADIIKIAGNDG-------------------------------GAQALQAV--IEHGPTLRQHGFNLADIVKMAGNIGGAQALQAVLDLKPVLD
Mycetohabitans sp. M7 (MN840539)RERSFSQPDIVKMAGNNG-------------------------------GAQALQAV--LELDPAFRERGFNPTDIVKIAGNNGGAQALQAVLELEPALR
Mycetohabitans sp. M7 (MN840540)RERGFSQPDIVKMAGNDG-------------------------------GAQALQAG--LELDPAFRERGFNPTDIVKIAGNIGGAQALQAVLELEPAFR
Mycetohabitans sp. M8 (MN840541)RECDFRQADIVKIAGNGG-------------------------------SAQALKAV--LEHGPTLRQRGFSRADIVKIAANGGGAQALQAVLK------
M. rhizoxinica M1 (E5AW45)      RERGFNQATIVKIAANGGGAQALYSVLDVEPTLDKRGFSRVDIVKIAGGGAQALHTA--FELEPTLRKRGFNPTDIVKIAGNKGGAQALQAVLELEPALR
Mycetohabitans sp. M2 (MN840542)RERGFNQATIVKIAANGGGAQALYSVLDVEPTLDKRGFSRVDIVKIAGGGAQALHTA--FELEPTLRKRGFNPTDIVKIAGNKGGAQALQAVLELEPALR
Mycetohabitans sp. M3 (MN840545)REHGFSRADIVRIAGNGG-------------------------------GAQALYSV--LDVGLTLGKRSFSRADIVKMARNDGGAQALHTVLKLEPTLG
Mycetohabitans sp. M6 (MN840543)RERGFNQATIVKIAANGGGAQALYSVLDVEPTLDKRGFSRVDIVKIAGGGAQALHTA--FELEPTLRKRGFNPTDIVKIAGNKGGAQALQAVLELEPALR
Mycetohabitans sp. M7 (MN840544)REHGFSRADIVRIAGNGG-------------------------------GAQALYSV--LDVGPTLGKRGFSRADIVKIAGNGGGAQALHTVFKLEPTLG
M. rhizoxinica M1 (E5AW43)      NERDYSGADIVKIAGNGG-------------------------------GARALKAV--VMHGPTLCESGYSGADIVKIASNGGGAQALEAVAM------
Mycetohabitans sp. M2 (MN840547)NERDYSGADIVKIAGNGG-------------------------------GARALKAV--VMHGPTLCESGYSGADIVKIASNGGGAQALEAVAM------
Mycetohabitans sp. M4 (MN840549)RQRGFARVDIIKITGNDG-------------------------------GAQALKAV--IVHGSTLNERGYSGANIVKIAGNGGGARAIEAVVM------
Mycetohabitans sp. M6 (MN840546)NERDYSGADIVKIAGNGG-------------------------------GARALKAV--VMHGPTLCESGYSGADIVKIASNGGGAQALEAVAM------
Mycetohabitans sp. M7 (MN840548)RQRGFARVDIIKITGNDG-------------------------------GAQALKAV--VVHGPTLNERGYSGADIVKIAGNGGGAWALKAVVM------
AvrXa10 X. oryzae (Q56830)      QTHGLTPDQVVAIANHDG-------------------------------GKQALETV-QRLLPVLCQDHGLTPDQVVAIASNIGGKQALATVQR-LLPVL
AvrBs3 X. euvesicatoria (P14727)QAHGLTPEQVVAIASHDG-------------------------------GKQALETV-QRLLPVLCQAHGLTPEQVVAIASHDGGKQALETVQR-LLPVL
Brg11 R. solanacearum (Q8XYE3)   APYGLSTEQVVAIASHDG-------------------------------GKQALEAVGAQLVALRAAPYALSTEQVVAIASNKGGKQALEAVKAQLLELR

                                       510       520       530       540       550       560       570       580       590       600
                                ....|....|....|....|....|....|....|....|....|....|....|....|....|....|....|....|....|....|....|....|
M. rhizoxinica M1 (E5AV36)      EHGF---------------------------------SQPDIVKMAGNIGGAQALQAV--LSLGPALRERGFSQPDIVKIAGNTGGAQALQAVLDLELTL
Mycetohabitans sp. M2 (MN891944)EHGF---------------------------------SQPDIVKMAGNIGGAQALQAV--LSLGPALRERGFSQPDIVKIAGNTGGAQALQAVLDLELTL
Mycetohabitans sp. M4 (MN840538)ERGF---------------------------------SQPDIVKMAGNNGGAQALQAG--LELEPAFRERGFNPTDMVKIAGNNGGAQALQAVLELEPAL
M. endofungorum M5 (MN891945)   EHGF---------------------------------SQPDIVKMAGNIGGAQALQAV--LSLGPALRERGFSQPDIVKIAGNTGGAQALQAVLDLELTL
Mycetohabitans sp. M6 (MN840537)EHGF---------------------------------SQPDIVKMAGNIGGAQALQAV--LSLGPALRERGFSQPDIVKIAGNTGGAQALQAVLDLELTL
Mycetohabitans sp. M7 (MN840539)ERGF---------------------------------SQPDIVKMASNSGGAQALQAG--LELEP-----------------------------------
Mycetohabitans sp. M7 (MN840540)ERGF---------------------------------SQPDIVKMAGNSGGAQALQAG--LELEPAFRERGFNPTDIVKIAGNNGGAQALQAVLELEPAL
Mycetohabitans sp. M8 (MN840541)----------------------------------------------------------------------------------------------------
M. rhizoxinica M1 (E5AW45)      ERGFNQATIVKMAGNAGGAQALYSVLDVEPALRERGFSQPEIVKIAGNIGGAQALHTV--LELEPTLHKRGFNPTDIVKIAGNSGGAQALQAVLELEPAF
Mycetohabitans sp. M2 (MN840542)ERGFNQATIVKMAGNAGGAQALYSVLDVEPALRERGFSQPEIVKIAGNIGGAQALHTV--LELEPTLHKRGFNPTDIVKIAGNSGGAQALQAVLELEPAF
Mycetohabitans sp. M3 (MN840545)ERGF---------------------------------SRGDIVKMAGNIGGAQALKAV--LE--------------------------------------
Mycetohabitans sp. M6 (MN840543)ERGFNQATIVKMAGNAGGAQALYSVLDVEPALRERGFSQPEIVKIAGNIGGAQALHTV--LELEPTLHKRGFNPTDIVKIAGNSGGAQALQAVLELEPAF
Mycetohabitans sp. M7 (MN840544)ERGF---------------------------------SRGDIVKMAGNIGGAQALQAV--LELEPAFHERSFCQPDIVKMAGNIGGAQALQAVLELEPAF
M. rhizoxinica M1 (E5AW43)      ----------------------------------------------------------------------------------------------------
Mycetohabitans sp. M2 (MN840547)----------------------------------------------------------------------------------------------------
Mycetohabitans sp. M4 (MN840549)----------------------------------------------------------------------------------------------------
Mycetohabitans sp. M6 (MN840546)----------------------------------------------------------------------------------------------------
Mycetohabitans sp. M7 (MN840548)----------------------------------------------------------------------------------------------------
AvrXa10 X. oryzae (Q56830)      CQAHG-------------------------------LTPDQVVAIASHDGGKQALETV-QRLLPVLCQDHGLTPDQVVAIASNNGGKQALETVQRLLPVL
AvrBs3 X. euvesicatoria (P14727)CQAHG-------------------------------LTPQQVVAIASNGGGKQALETV-QRLLPVLCQAHGLTPEQVVAIASNSGGKQALETVQALLPVL
Brg11 R. solanacearum (Q8XYE3)   GAPYA-------------------------------LSTAQVVAIASHDGGNQALEAVGTQLVALRAAPYALSTEQVVAIASHDGGKQALEAVGAQLVAL
```

```
                                                 610       620       630       640       650       660       670       680       690       700
                                        ....|....|....|....|....|....|....|....|....|....|....|....|....|....|....|....|....|....|....|....|
M. rhizoxinica M1 (E5AV36)              VEHGFSQPDIVRITGNRGGAQALQAVLALELTLRERGFSQPDIVKIAGNSGGAQALQAVLDLELTFRER--GFSQADIVKIAGNDGGTQALHAVLDLERM
Mycetohabitans sp. M2 (MN891944)        VEHGFSQPDIVRITGNRGGAQALQAVLALELTLRERGFSQPDIVKIAGNSGGAQALQAVLDLELTFRER--GFSQADIVKIAGNDGGTQALHAVLDLERM
Mycetohabitans sp. M4 (MN840538)        RERGFSQPDIVKMASNSGGAQALQVVLELEPTLRERDFRQADIVKIASNGGSAQALNAVIKLGPTLRQR--GFSRADIVKIAAN----------------
M. endofungorum M5 (MN891945)           VERGFSQPDIVRITGNRGGAQALQAVLALELTLRERGFSQPDIVKIAGNSGGAQALHAVLDLELTFRER--GFSQADIVKIAGNDGGTQALHAVLDLERM
Mycetohabitans sp. M6 (MN840537)        VEHGFSQPDIVRITGNRGGAQALQAVLALELTLRERGFSQPDIVKIAGNSGGAQALQAVLDLELTFRER--GFSQADIVKIAGNDGGTQALHAVLDLERM
Mycetohabitans sp. M7 (MN840539)        -------------------------------TLRERDFRQADIVKMASNGGSAQALNAVIKLGPTLRQR--GFSRADIVKIAAN----------------
Mycetohabitans sp. M7 (MN840540)        RERSFSQPDIVKMASNSGGAQALQAVLVLEPTLRERDFRQADIVKMASNGGSAQALNAVIKLGPTLRQR--GFSRADIVKIAAN----------------
Mycetohabitans sp. M8 (MN840541)        ----------------------------------------------------------------------------------------------------
M. rhizoxinica M1 (E5AW45)              RERGFGQPDIVKMASNIGGAQALQAVLELEPALRERGFSQPDIVEMAGNIGGAQALQAVLELEPAFRER--GFSQSDIVKIAGNIGGAQALQAVLELEPT
Mycetohabitans sp. M2 (MN840542)        RERGFGQPDIVKMASNIGGAQALQAVLELEPALRERGFSQPDIVEMAGNIGGAQALQAVLELEPAFRER--GFSQSDIVKIAGNIGGAQALQAVLELEPT
Mycetohabitans sp. M3 (MN840545)        ----------------------------------------------------------------------------------------------------
Mycetohabitans sp. M6 (MN840543)        RERGFGQPDIVKMASNIGGAQALQAVLELEPALRERGFSQPDIVEMAGNIGGAQALQAVLELEPAFRER--GFSQSDIVKIAGNIGGAQALQAVLELEPT
Mycetohabitans sp. M7 (MN840544)        RERGFSRGDIVKMAGNIGGAQALQAGLELEPAFRERGFSRGDIVKMAGNIGGAQALQAVLELEPAFREH--GFSQPDIVKIAGNIGGAQALQAVLELEPA
M. rhizoxinica M1 (E5AW43)              ----------------------------------------------------------------------------------------------------
Mycetohabitans sp. M2 (MN840547)        ----------------------------------------------------------------------------------------------------
Mycetohabitans sp. M4 (MN840549)        ----------------------------------------------------------------------------------------------------
Mycetohabitans sp. M6 (MN840546)        ----------------------------------------------------------------------------------------------------
Mycetohabitans sp. M7 (MN840548)        ----------------------------------------------------------------------------------------------------
AvrXa10 X. oryzae (Q56830)              CQD--------------------------------HGLTPAQVVAIANHGGGKQALETVQRLLPVLCQD-HGLTPVQVVAIASN----------------
AvrBs3 X. euvesicatoria (P14727)        CQA--------------------------------HGLTPEQVVAIASNSGGKQALETVQRLLPVLCQA-HGLTPEQVVAIASH----------------
Brg11 R. solanacearum (Q8XYE3)          RAAP-------------------------------YALNTEQVVAIASSHGGKQALEAVRALFPDLRAAPYALSTAQLVAIASN----------------

                                                 710       720       730       740       750       760       770       780       790       800
                                        ....|....|....|....|....|....|....|....|....|....|....|....|....|....|....|....|....|....|....|....|
M. rhizoxinica M1 (E5AV36)              LGERGFSRADIVNVAGNNGGAQALKAVLEHEATLNERGFSRADIVKIAGNGGGAQALKAVLEHEATLDER--GFSRADIVRIAGNGGGAQALKAVLEHGP
Mycetohabitans sp. M2 (MN891944)        LGERGFSRADIVNVAGNNGGAQALKAVLEHEATLNERGFSRADIVKIAGNGGGAQALKAVLEHEATLDER--GFSRADIVRIAGNGGGAQALKAVLEHGP
Mycetohabitans sp. M4 (MN840538)        --------------------------------------------------GGGAQALQAVLKHGPTLGER--GFTLTDIVEMAGNNGGAQALKAVLEHGS
M. endofungorum M5 (MN891945)           LGERGFSRADIVNVAGNNGGAQALKAVLEHEATLNERGFSRADIVKIAGNGGGAQALKAVLEHEATLDER--GFSRADIVRIAGNGGGAQALKAVLEHGP
Mycetohabitans sp. M6 (MN840537)        LGERGFSRADIVNVAGNNGGAQALKAVLEHEATLNERGFSRADIVKIAGNGGGAQALKAVLEHEATLDER--GFSRADIVRIAGNGGGAQALKAVLEHGP
Mycetohabitans sp. M7 (MN840539)        --------------------------------------------------GGGAQALQAVLKHGPTLGER--GFTLTDIVKMASNNGGAQALKAVLEHGP
Mycetohabitans sp. M7 (MN840540)        --------------------------------------------------GGGAQALQAVLKHGPTLGER--GFTLTDIVKMAGNNGGAQALKAVLEHGP
Mycetohabitans sp. M8 (MN840541)        --------------------------------------------------------------HGPTLDER--GFTLTDIVKMAGNVGGAQALKAVLEHGP
M. rhizoxinica M1 (E5AW45)              LRESDFRQADIVNIAGNDGSTQALKAVIEHGPRLRQRGFNRASIVKIAGNSGGAQALQAVLKHGPTLDER--GFNLTNIVKIAGNGGGAQALKAVIEHGP
Mycetohabitans sp. M2 (MN840542)        LRESDFRQADIVNIAGNDGSTQALKAVIEHGPRLRQRGFNRASIVKIAGNSGGAQALQAVLKHGPTLDER--GFNLTNIVKIAGNGGGAQALKAVIEHGP
Mycetohabitans sp. M3 (MN840545)        --------------------------------------------------------------HGPTLDER--GFTLTDIVKMAGNIGGAQALKAVLEHGP
Mycetohabitans sp. M6 (MN840543)        LRESDFRQADIVNIAGNDGSTQALKAVIEHGPRLRQRGFNRASIVKIAGNSGGAQALQAVLKHGPTLDER--GFNLTNIVKIAGNGGGAQALKAVIEHGP
Mycetohabitans sp. M7 (MN840544)        FRERGFSQSNIVKIAGNIGGAQALQAVLELEPMLRECDFRQTDIVKMAGSGGSAQALNAVIKHGPTLRQR--GFSQADIVKMAGNNGGAQALKAVIEHGP
M. rhizoxinica M1 (E5AW43)              --------------------------------------------------------------HGSTLCER--GYCRTDIAKIAGNGGGAQALKAIVMHGP
Mycetohabitans sp. M2 (MN840547)        --------------------------------------------------------------HGSTLCER--GYCRTDIAKIAGNGGGAQALKAIVMHGP
Mycetohabitans sp. M4 (MN840549)        --------------------------------------------------------------HGPTLCER--GYSRTDIVKITDNNGGAQALKAVFEHGP
Mycetohabitans sp. M6 (MN840546)        --------------------------------------------------------------HGSTLCER--GYCRTDIAKIAGNGGGAQALKAIVMHGP
Mycetohabitans sp. M7 (MN840548)        --------------------------------------------------------------HGPTLCER--GYSRTDIVKIADNNGGAQALKAVFEHGP
AvrXa10 X. oryzae (Q56830)              --------------------------------------------------SGGKQALETVQRLLPVLCQD-HGLTPVQVVAIASNGGGKQALATVQRLLP
AvrBs3 X. euvesicatoria (P14727)        --------------------------------------------------DGGKQALETVQRLLPVLCQA-HGLTPEQVVAIASHDGGKQALETVQRLLP
Brg11 R. solanacearum (Q8XYE3)          --------------------------------------------------PGGKQALEAVRALFRELRAAPYALSTEQVVAIASNHGGKQALEAVRALFR
```

```
                                        810       820       830       840       850       860       870       880       890       900
                                 ....|....|....|....|....|....|....|....|....|....|....|....|....|....|....|....|....|....|....|....|
M. rhizoxinica M1 (E5AV36)       --TLNERGFNLTDIVEMAANS---------------------------------GGAQALKAVLEHGPTLRQRGLSLI--DIVEIASN-GGAQALKAVLK
Mycetohabitans sp. M2 (MN891944) --TLNERGFNLTDIVEMAANS---------------------------------GGAQALKAVLEHGPTLRQRGLSLI--DIVEIASN-GGAQALKAVLK
Mycetohabitans sp. M4 (MN840538) --TLDERGFTLTDIVKMAGNNGGAQALKAVLEHGPTLQQRGFTLTDIVKMAGNIGGAQALKAVLEHGPTLRQRDLSLI--DIVEIASN-GGAQALKAVLK
M. endofungorum M5 (MN891945)    --TLNERGFNLTDIVEMAANS---------------------------------GGAQALKAVLEHGPTLRQRGLSLI--DIVEIASN-GGAQALKAVLK
Mycetohabitans sp. M6 (MN840537) --TLNERGFNLTDIVEMAANS---------------------------------GGAQALKAVLEHGPTLRQRGLSLI--DIVEIASN-GGAQALKAVLK
Mycetohabitans sp. M7 (MN840539) --TLDERGFTLTDIVKMAG---------------------------------NIGGAQALKTVLGHGPTLRQRDLSLI--DIVEIASN-GGAQALKAVLK
Mycetohabitans sp. M7 (MN840540) --TLDERGFTLTDIVKMAGNNGGAQALKAVLEHGPTLDERGFTLTDIVKMAGNIGGAQALKVVLEHGPTLRQRDLSLI--DIVEIASN-GGAQALKAVLK
Mycetohabitans sp. M8 (MN840541) --TLRQRDLSLIDIVEIASN----------------------------------GGAQALKAVLKYGPVLTQVG--------------------------
M. rhizoxinica M1 (E5AW45)       --TLQQRGFNLTDIVEMAGKGGGAQALKAVLEHGPTLRQRGFNLIDIVEMASNTGGAQALKTVLEHGPTLRQRDLSLI--DIVEIASN-GGAQALKAVLK
Mycetohabitans sp. M2 (MN840542) --TLQQRGFNLTDIVEMAGKGGGAQALKAVLEHGPTLRQRGFNLIDIVEMASNTGGAQALKTVLEHGPTLRQRDLSLI--DIVEIASN-GGAQALKAVLK
Mycetohabitans sp. M3 (MN840545) --TLRQRDLSLIDIVEIASN----------------------------------GGAQALKAVLKYGPVLTQAGRSNE--EIVHVAARRGGAGRIRKMVA
Mycetohabitans sp. M6 (MN840543) --TLQQRGFNLTDIVEMAGNGGGAQALKAVLEHGPTLRQRGFNLIDIVEMASNTGGAQALKTVLEHGPTLRQRDLSLI--DIVEIASN-GGAQALKAVLK
Mycetohabitans sp. M7 (MN840544) --TLDERGFTLTDIVKMAGNN---------------------------------GGAQALKVVLEHGPTLRQRGFSQT--AIVKMAGNIGGAQALKAVLK
M. rhizoxinica M1 (E5AW43)       --TLCERGYSRTDIVKIADNN---------------------------------GGAQALKAVFEHGPALTQAG--------------------------
Mycetohabitans sp. M2 (MN840547) --TLCERGYSRTDIVKIADNN---------------------------------GGAQALKAVFEHGPALTQAG--------------------------
Mycetohabitans sp. M4 (MN840549) --AFTQAGRSNEDIVDMAARI---------------------------------GAAGQIRKMAAQ---LSKRQ--------------------------
Mycetohabitans sp. M6 (MN840546) --TLCERGYSRTDIVKIADNN---------------------------------GGAQALKAVFEHGPALTQAG--------------------------
Mycetohabitans sp. M7 (MN840548) --ALTQAG--------------------------------------------------------------------------------------------
AvrXa10 X. oryzae (Q56830)       -VLCQDHGLTPVQVVAIASHD---------------------------------GGKQALETVQRLLPVL-CQDHGLTPDQVVAIASNGG-KQALESIVA
AvrBs3 X. euvesicatoria (P14727) -VLCQAHGLTPEQVVAIASHD---------------------------------GGKQALETVQRLLPVL-CQAHGLTPQQVVAIASNGGGRPALETVQR
Brg11 R. solanacearum (Q8XYE3)    GLRAAPYGLSTAQVVAIASSN---------------------------------GGKQALEAVWALLPVLRATPYDLNTAQIVAIASHDGGKPALEAVWA


                                 ....|....
M. rhizoxinica M1 (E5AV36)       YGPVLMQAG
Mycetohabitans sp. M2 (MN891944) YGPVLMQAG
Mycetohabitans sp. M4 (MN840538) YGPVLTQAG
M. endofungorum M5 (MN891945)    YGPVLMQAG
Mycetohabitans sp. M6 (MN840537) YGPVLMQAG
Mycetohabitans sp. M7 (MN840539) YGPVLTQAG
Mycetohabitans sp. M7 (MN840540) YGPVLIQAG
Mycetohabitans sp. M8 (MN840541) ---------
M. rhizoxinica M1 (E5AW45)       YGPVLMQAG
Mycetohabitans sp. M2 (MN840542) YGPVLMQAG
Mycetohabitans sp. M3 (MN840545) S---LLGGN
Mycetohabitans sp. M6 (MN840543) YGPVLMQAG
Mycetohabitans sp. M7 (MN840544) YGPVLMQAG
M. rhizoxinica M1 (E5AW43)       ---------
Mycetohabitans sp. M2 (MN840547) ---------
Mycetohabitans sp. M4 (MN840549) ---------
Mycetohabitans sp. M6 (MN840546) ---------
Mycetohabitans sp. M7 (MN840548) ---------
AvrXa10 X. oryzae (Q56830)       QLSRPDPAL
AvrBs3 X. euvesicatoria (P14727) LLPVLCQAH
Brg11 R. solanacearum (Q8XYE3)   KLP------
```

Fig. S3. Alignment of predicted TAL effector proteins from endofungal *Mycetohabitans* species (MTAL) and plant pathogenic *R. solanaceraum* and *Xanthomonas* species. The core repeats of each protein were used for alignment (1). Sequences identified in this study are highlighted in bold (Table S3). GenBank accession numbers are indicated in brackets.

513 **Table S1.** Fungal strains used in this study that harbor bacterial endosymbionts (2).

**Taxon**	**Strain designation**	**Origin**	**Bacterial endosymbiont (isolate)**
Rhizopus microsporus van Tieghem	ATCC 62417	Rice seedlings, Japan	*Mycetohabitans rhizoxinica* HKI-454 (M1)
Rhizopus sp. strain F-1360	ATCC 20577	Soil, Japan	*Mycetohabitans* sp. strain HKI-512 (M2)
Rhizopus microsporus Tieghem var. *microsporus*	CBS 111563	Sufu starter culture, rice wine tablet, Vietnam	*Mycetohabitans* sp. strain HKI-455 (M3)
Rhizopus microsporus Tieghem var. *microsporus*	CBS 699.68	Soil, Ukraine	*Mycetohabitans* sp. strain HKI-402 (M4)
Rhizopus microsporus Tieghem	CBS 112285	Ground nuts, Mozambique	*Mycetohabitans endofungorum* HKI-456 (M5)
Rhizopus microsporus var. *chinensis* (Saito) Schipper & Stalpers	CBS 261.28	Not specified, USA	*Mycetohabitans* sp. strain HKI-513 (M6)
Rhizopus microsporus Tieghem var. *microsporus*	CBS 700.68	Forest soil, Georgia	*Mycetohabitans* sp. strain HKI-403 (M7)
Rhizopus microsporus Tieghem var. *microsporus*	CBS 308.87	Man, from deep necrotic tissue within the hand following a spider bite, Australia	*Mycetohabitans* sp. strain HKI-404 (M8)

514

Table S2. Predicted nuclear localization sequences (NLS) within AWR proteins from endofungal *Mycetohabitans* and plant-associated *Burkholderia* species, and plant pathogenic *R. solanaceraum* using (A) NucPred prediction software (3) and (B) cNLS Mapper (4). GenBank accession numbers are given in brackets.

**A**	**Species (Gen. Bank Acc. No)**	**NLS motif (monopartite)**	**Position in protein[1]**	**NucPred Score[2]**	**Specificity[3]**	**Sensitivity (coverage)[4]**
	Ralstonia solanacearum (BAH47286.1) AWR1	GATRRRRNSG	38 – 45	0.75	0.81	0.44
	Paraburkholderia graminis (WP 006047106.1)	TRRRM	36 – 40	0.62	0.71	0.53
	Burkholderia glumae (WP 012733093.1)	QARRRQG	78 – 84	0.55	0.70	0.62
	Burkholderia sp. CCGE1003 (ADN59567.1)	DPQRRRRFVDD	693 – 693	0.62	0.71	0.53
	Mycetohabitans rhizoxinica HKI-454 (M1)	KRSRKRKATV	577 – 586	0.68	0.71	0.53
	Mycetohabitans sp. strain HKI-512 (M2)	KRSRKRKATV	577 – 586	0.68	0.71	0.53
	Mycetohabitans sp. strain HKI-455 (M3)	KRSRKRKATV	572 – 581	0.75	0.81	0.44
	Mycetohabitans sp. strain HKI-402 (M4)	KRSRKRKATV	572 – 581	0.75	0.81	0.44
	Mycetohabitans endofungorum HKI-456 (M5)	KRSRKRKATV	572 – 581	0.68	0.71	0.53
	Mycetohabitans sp. strain HKI-513 (M6)	KRSRKRKATV	577 – 586	0.69	0.71	0.53
	Mycetohabitans sp. strain HKI-403 (M7)	KRSRKRKATV	572 – 581	0.73	0.81	0.44
	Mycetohabitans sp. strain HKI-404 (M8)	KRSRERKATV	559 – 568	0.62	0.71	0.53

B	Species (Gen. Bank Acc. No)	NLS motif (monopartite)	Position in protein[1]	cNLS Score[5]	NLS motif (bipartite)	Position in protein[1]	cNLS Score[5]
	Ralstonia solanacearum (BAH47286.1) AWR1	N/A	N/A	N/A	N/A	N/A	N/A
	Paraburkholderia graminis (WP 006047106.1)	N/A	N/A	N/A	N/A	N/A	N/A
	Burkholderia glumae (WP 012733093.1)	N/A	N/A	N/A	N/A	N/A	N/A
	Burkholderia sp. CCGE1003 (ADN59567.1)	N/A	N/A	N/A	N/A	N/A	N/A
	Mycetohabitans rhizoxinica HKI-454 (M1)	RSRKRKATVQ	578 – 587	13	KATKATKATKLPKLPKLPKLPKLPKLP	1083 – 1096	5.5
					KATKATKATKLPKLPKLPKLPKLPKLPKLP	1083 – 1112	6.1
					KATKATKLPKLPKLPKLPKLPKLPKLPKG	1086 – 1114	5.6
	Mycetohabitans sp. strain HKI-512 (M2)	RSRKRKATVQ	578 – 587	13	FMGKPVAATKATKLPKLPKLPKLPKLPKLP	1061 – 1090	5.5
					KATKLPKLPKLPKLPKLPKLPKLPKG	1070 – 1095	5.5
	Mycetohabitans sp. strain HKI-455 (M3)	RSRKRKATVQ	573 – 582	13	N/A	N/A	N/A
	Mycetohabitans sp. strain HKI-402 (M4)	RSRKRKATVQ	573 – 582	13	N/A	N/A	N/A
	Mycetohabitans endofungorum HKI-456 (M5)	RSRKRKATVQ	573 – 582	13	N/A	N/A	N/A
	Mycetohabitans sp. strain HKI-513 (M6)	RSRKRKATVQ	578 – 587	13	KATKATKATKLPKLPKLPKLPKLP	1070 – 1096	5.5
					KATKATKATKLPKLPKLPKLPKLPKLPKG	1070 – 1098	5.5
	Mycetohabitans sp. strain HKI-403 (M7)	RSRKRKATVQ	573 – 582	13	N/A	N/A	N/A
	Mycetohabitans sp. strain HKI-404 (M8)	N/A	N/A		N/A	N/A	N/A

[1] Amino acid position of predicted NLS within AWR peptide sequence.
[2] Fraction of proteins predicted to be nuclear that actually are nuclear.
[3] Fraction of true nuclear proteins that are predicted (coverage).
[4] cNLS score: > 10: protein exclusively localized to the nucleus; 7 – 8: partially localized to the nucleus; 3 – 5: localized to both the nucleus and the cytoplasm; 1 – 2: localized to the cytoplasm.
N/A: not available.

523 **Table S3.** GenBank accession numbers of genes identified in this study.

Gene	Locus Tag	Strain	GenBank Acc. No.
awr	RBRH 02721	*Mycetohabitans rhizoxinica* HKI-512 (M2)	MN840555
awr	RBRH 00074	*Mycetohabitans rhizoxinica* HKI-455 (M3)	MN840550
awr	RBRH 00457	*Mycetohabitans rhizoxinica* HKI-402 (M4)	MN840552
awr	RBRH 01365	*Mycetohabitans endofungorum* HKI-456 (M5)	MN840553
awr	RBRH 02790	*Mycetohabitans rhizoxinica* HKI-513 (M6)	MN840556
awr	RBRH 03309	*Mycetohabitans rhizoxinica* HKI-403 (M7)	MN840551
awr	RBRH 02340	*Mycetohabitans rhizoxinica* HKI-404 (M8)	MN840554
**mtal1*	*RBRH 01844	*Mycetohabitans* sp. HKI-512 (M2)	*MN891944
mtal1	RBRH 01585	*Mycetohabitans rhizoxinica* HKI-402 (M4)	MN840538
**mtal1*	*RBRH 01844	*Mycetohabitans endofungorum* HKI-456 (M5)	*MN891945
mtal1	RBRH 01580	*Mycetohabitans rhizoxinica* HKI-513 (M6)	MN840537
mtal1	RBRH 01934	*Mycetohabitans rhizoxinica* HKI-403 (M7)	MN840539
mtal1	RBRH 02907	*Mycetohabitans rhizoxinica* HKI-403 (M7)	MN840540
mtal1	RBRH 00422	*Mycetohabitans rhizoxinica* HKI-404 (M8)	MN840541
mtal2	RBRH 00895	*Mycetohabitans* sp. HKI-512 (M2)	MN840542
mtal2	RBRH 01901	*Mycetohabitans rhizoxinica* HKI-455 (M3)	MN840545
mtal2	RBRH 01694	*Mycetohabitans rhizoxinica* HKI-513 (M6)	MN840543
mtal2	RBRH 02271	*Mycetohabitans rhizoxinica* HKI-403 (M7)	MN840544
mtal3	RBRH 00894	*Mycetohabitans* sp. HKI-512 (M2)	MN840547
mtal3	RBRH 01581	*Mycetohabitans rhizoxinica* HKI-402 (M4)	MN840549
mtal3	RBRH 01695	*Mycetohabitans rhizoxinica* HKI-513 (M6)	MN840546
mtal3	RBRH 01772	*Mycetohabitans rhizoxinica* HKI-403 (M7)	MN840548

524 * Sequences identified via amplification and Sanger sequencing.

Table S4. (A) Primers used for the amplification of *mtal1* partial coding sequences in polymerase chain reactions. **(B)** Primers used for generating a T3SS-associated *awr* mutant strain (*M. rhizoxinica* Δ*awr*) or *M. rhizoxinica mtal* mutant strains (*M. rhizoxinica* Δ*mtal2* or *M. rhizoxinica* Δ*mtal3*). Control primers used in colony PCRs to confirm mutant strains are also listed. **(C)** Primers used for generating genetically complemented *M. rhizoxinica mtal* mutant strains (*M. rhizoxinica* Δ*mtal2* pBBR-*mtal2* and *M. rhizoxinica* Δ*mtal3* pBBR-*mtal3*) or *M. rhizoxinica mtal* mutant strains containing a relevant empty control vector (*M. rhizoxinica Δmtal2* pBBR∅ and *M. rhizoxinica Δmtal3* pBBR∅).

A

Gene	GenBank acc. no.	Primer Sequence (5' - 3')		Predicted amplicon size (bp)
mtal1	RBRH_01844	GCTACCACCTTGGCGTTTAT	Forward	3100 bp
		TACTTTGCGCGTAACAGCAC	Reverse	

B

Gene	Type	Name	Oligo sequence (5' -> 3')
**RBRH 03012 (*awr*)**	**Vector construction**	Awr ArmA Fw	ctatagggcgaattgggtacgttgatccatgatgtagacgg
		Awr ArmA Rv	acattcatccctggctgagaatgtcaacgg
		Awr ArmB Fw	gttcttctgaaaatttagtcgacaagcgcg
		Awr ArmB Rv	ctcgaggggggggcccggtacaacgttgccaagcggataaaatg
		Kan Awr Fw	tctcagccagggatgaatgtcagctactgg
		Kan Awr Rv	gactaaattttcagaagaactcgtcaagaag
	**Control Primers**	Awr CA fw	aatactgctggaggccaatg
		Awr C rv	gcttaccacgaagatggacg
		Awr D fw	gttcaccaccgcttcgaaac
		Awr DB rv	aattgtcgaccgaaatcagc
		Kan B Fw	agtgacaacgtcgagcacag
		Kan A Rv	cgttggctacccgtgatatt
**RBRH 01776 (*mtal2*)**	**Vector construction**	Tal1776 ArmA Fw (SpeI)	gatcactagtgccggaccgatattgtaaag
		Tal1776 ArmA Rv	cggaatgaattcactactgggtcaaaccgatgccgaac
		Tal1776 ArmB Fw	gctgagaattcagtagtaggcagcggttaccagcattg
		Tal1776 ArmB Rv (KpnI)	ctagggtaccagtgctgcgtcatagtcgtg
		Kan Tal Fw	agtagtgaattcattccggggatcagaagaactcgtcaagaaggc
		Kan Tal Rv	actactgaattctcagccaatcggatgaatgtcagctactgggc
	**Control Primers**	77 D Fw	taacgagcgcgactacagtg
		76 C Rv	gttcgagtgggctgaggtta
		76 D Fw	caatcgagcgagtatcgtca
		76 DB Rv	cacgccatatgtcatcgttc
		Kan B Fw	cgttggctacccgtgatatt

Gene	Type	Name	Sequence
		Kan A Rv	agtgacaacgtcgagcacag
RBRH 01777 (*mtal3*)	Vector construction	Tal1777 ArmA Fw(SpeI)	gatcactagtcctgcagcgccttactattc
		Tal1777 ArmA Rv	cggaatgaattcactactaatcccactgacgttgggtcattc
		Tal1777 ArmB Fw	gctgagaattcagtagtcgaaagatggctgcacaattgtc
		Tal1777 ArmB Rv (KpnI)	ctagggtacccaacgtcaagcaccgaatac
		Kan Tal Fw	agtagtgaattcattccggggatcagaagaactcgtcaagaaggc
		Kan Tal Rv	actactgaattctcagccaatcggatgaatgtcagctactgggc
	Control Primers	77 CA Fw	cgttgatgtcggttcaacac
		77 C Rv	gccattgttatcggcaatct
		77 D Fw	taacgagcgcgactacagtg
		77 DB Rv	ctggtaagctgagcccaaac
		Kan B Fw	cgttggctacccgtgatatt
		Kan A Rv	agtgacaacgtcgagcacag

C

Gene	Type	Name	Oligo binding sequence with overhang (5' → 3')
RBRH 01776 (*mtal2*)	Vector construction	*mtal2* fw	GCTTCGAAAGGACAAGCATATGCCGGCGACGTCTATGCATC
		mtal2 rv	TGGTCCTCGCGCCAGCTTAAGCTATTGCCGCTCCAGCAAGAGTG
RBRH 01777 (*mtal3*)	Vector construction	*mtal3* fw	GCTTCGAAAGGACAAGCATATGCCGGTGACGTCTGTGTATC
		mtal3 rv	TGGTCCTCGCGCCAGCTTAAGCTATTGCCGCCCCGACAATTG
gfp	Vector construction	*gfp* fw	GCGGCAATAGCTGACAATCGGATCGAGCTTCG
		gfp rv	TGGTCCTCGCGCCAGCTTAAGTTATTTGTATAGTTCATCCATGCCATG

**Insert**	**Control Primers**	*cm*^{r} seq fw	GGGTATGTGGTCGAAGGC
		BBR seq rv	GCAAGGCGACAAGGTGCTG
**Colony PCR**	**Control Primer**	pBBR_verif_fw	ATTAATTATCCACAGAATCAGGG
		pBBR_verif_rv	CGGCTACATCATTCACTTTTTC

Table S5. Predicted nuclear localisation signal (NLS) within TAL effector proteins from endofungal *Mycetohabitans* species (MTALs) using the NucPred prediction software (3).

**Species**	**MTAL protein**	**NucPred Predictions[1]**			**NLS**
		**Score**	**Specificity[2]**	**Sensitivity[3]**	
Mycetohabitans rhizoxinica HKI-454 (M1)	MTAL1	0.23	0.52	0.83	RIRK
	MTAL2	0.27	0.52	0.83	RIRK
	MTAL3	0.08	N/A	N/A	QIRK
Mycetohabitans sp. strain HKI-512 (M2)	MTAL1	0.22	0.52	0.83	RIRK
	MTAL2	0.27	0.52	0.83	RIRK
	MTAL3	0.08	N/A	N/A	QIRK
Mycetohabitans sp. strain HKI-455 (M3)	MTAL2	0.14	0.45	0.88	RIRK
Mycetohabitans sp. strain HKI-402 (M4)	MTAL1	0.23	0.52	0.83	RIRK
	MTAL3	0.09	N/A	N/A	QIRK
Mycetohabitans endofungorum HKI-456 (M5)	MTAL1	0.22	0.52	0.83	RIRK
Mycetohabitans sp. strain HKI-513 (M6)	MTAL1	0.23	0.52	0.83	RIRK
	MTAL2	0.27	0.52	0.83	RIRK
	MTAL3	0.08	N/A	N/A	QIRK
Mycetohabitans sp. strain HKI-403 (M7)	MTAL1	0.17	0.45	0.88	RIRK
	MTAL2	0.16	0.45	0.88	RIRK
	MTAL3	0.05	N/A	N/A	QIRK
Mycetohabitans sp. strain HKI-404 (M8)	MTAL1	0.12	0.45	0.88	RIRK

[1] Nuclear localisation predicted with NucPred (3).
[2] Fraction of proteins predicted to be nuclear that actually are nuclear.
[3] Fraction of true nuclear proteins that are predicted (coverage).
N/A: not available.

Table S6. (A) Approximate probabilities (*p*) of Brown-Forsythe test, (B) one-way analysis of variance (ANOVA), and (C) Tukey HSD Post Hoc Test for fungal spore counts following co-cultivation of apo-symbiotic *R. microsporus* (RMapo) with *M. rhizoxinica* wild type (M1WT), *M. rhizoxinica mtal* mutant strains (*M. rhizoxinica* Δ*mtal1*, *M. rhizoxinica* Δ*mtal2*, and *M. rhizoxinica* Δ*mtal3*), or a T3SS-associated *awr* mutant strain (*M. rhizoxinica* Δ*awr*). Homogeneous data (non-significant Brown-Forsythe) is shown in black numbers. P-values with $p<0.05$ were considered statistically significant (marked in grey).

A

**Brown-Forsythe Test**	
F (DFn, DFd)	1.135 (5, 18)
P value	0.378
P value summary	ns
Are SDs significantly different (P < 0.05)?	No

B

**ANOVA Summary**	
F	202.4
P value	<0.0001
P value summary	****
Significant diff. among means (P<0.05)?	Yes
R squared	0.9825

**ANOVA Table**	**SS**	**DF**	**MS**	**F (DFn, DFd)**	**P value**
Treatment (between columns)	1E+18	5	2E+17	F (5, 18) = 202.4	P<0.0001
Residual (within columns)	1.78E+16	18	9.89E+14		
Total	1.02E+18	23			

C

**Strain Comparison**		**Mean Diff.**	**95% CI**			**Summary**	**P value**
RMapo	M1WT	−4.96E+08	−5.67E+08	to	−4.26E+08	****	<0.0001
	Δ*awr*	−4.49E+08	−5.20E+08	to	−3.78E+08	****	<0.0001
	Δ*mtal1*	−3.26E+07	−1.03E+08	to	3.81E+07	ns	0.6895
	Δ*mtal2*	−8.66E+07	−1.57E+08	to	−1.59E+07	*	0.0116
	Δ*mtal3*	−5.66E+07	−1.27E+08	to	1.41E+07	ns	0.1631
M1WT	Δ*awr*	4.74E+07	−2.33E+07	to	1.18E+08	ns	0.3159
	Δ*mtal1*	4.64E+08	3.93E+08	to	5.34E+08	****	<0.0001
	Δ*mtal2*	4.10E+08	3.39E+08	to	4.80E+08	****	<0.0001
	Δ*mtal3*	4.40E+08	3.69E+08	to	5.10E+08	****	<0.0001
Δ*awr*	Δ*mtal1*	4.16E+08	3.46E+08	to	4.87E+08	****	<0.0001
	Δ*mtal2*	3.62E+08	2.92E+08	to	4.33E+08	****	<0.0001
	Δ*mtal3*	3.92E+08	3.22E+08	to	4.63E+08	****	<0.0001
Δ*mtal1*	Δ*mtal2*	−5.40E+07	−1.25E+08	to	1.67E+07	ns	0.1982
	Δ*mtal3*	−2.40E+07	−9.47E+07	to	4.67E+07	ns	0.8833
Δ*mtal2*	Δ*mtal3*	3.00E+07	−4.07E+07	to	1.01E+08	ns	0.7549

Abbreviations: SS: sum of squares, DF: degrees of freedom, MS: mean square, Mean Diff.: mean difference.

Table S7. (A) Approximate probabilities (p) of Brown-Forsythe test, (B) one-way analysis of variance (ANOVA), and (C) Tukey HSD Post Hoc Test for fungal spore counts following co-cultivation of apo-symbiotic *R. microsporus* with *M. rhizoxinica* wild type (M1WT), *M. rhizoxinica mtal* mutant strains (*M. rhizoxinica Δmtal1*, *M. rhizoxinica Δmtal2*, and *M. rhizoxinica Δmtal3*), *M. rhizoxinica mtal* mutant strains containing the relevant empty vector (*M. rhizoxinica Δmtal1* pBBR∅, *M. rhizoxinica Δmtal2* pBBR∅, and *M. rhizoxinica Δmtal3* pBBR∅), or *M. rhizoxinica mtal* mutant strains expressing a plasmid-borne copy of the relevant *mtal* gene (*M. rhizoxinica Δmtal1* pBBR-*mtal1*, *M. rhizoxinica Δmtal2* pBBR-*mtal2*, and *M. rhizoxinica Δmtal3* pBBR-*mtal3*). Homogeneous data (non-significant Brown-Forsythe) is shown in black numbers. P-values with $p<0.05$ were considered statistically significant (marked in grey).

A

**Brown-Forsythe Test**	
F (DFn, DFd)	1.425 (9, 30)
P value	0.2217
P value summary	ns
Are SDs significantly different (P < 0.05)?	No

B

**ANOVA Summary**					
F	75.57				
P value	<0.0001				
P value summary	****				
Significant diff. among means (P<0.05)?	Yes				
R squared	0.9578				
**ANOVA Table**	**SS**	**DF**	**MS**	**F (DFn, DFd)**	**P value**
Treatment (between columns)	1.193E+18	9	1.325E+17	F (9, 30) = 75.57	P<0.0001
Residual (within columns)	5.262E+16	30	1.754E+15		
Total	1.246E+18	39			

C

**Strain Comparison**		**Mean Diff.**	**95% CI**			**Summary**	**P value**
M1WT	*Δmtal1*	4.64E+08	3.63E+08	to	5.65E+08	****	<0.0001
	Δmtal2	4.10E+08	3.09E+08	to	5.11E+08	****	<0.0001
	Δmtal3	4.40E+08	3.39E+08	to	5.41E+08	****	<0.0001
	Δmtal1 pBBR-*mtal1*	1.21E+08	2.02E+07	to	2.22E+08	**	0.0094
	Δmtal2 pBBR-*mtal2*	1.46E+08	4.52E+07	to	2.47E+08	***	0.001
	Δmtal3 pBBR-*mtal3*	1.21E+08	2.02E+07	to	2.22E+08	**	0.0094
	Δmtal1 pBBR∅	3.94E+08	2.93E+08	to	4.95E+08	****	<0.0001
	Δmtal2 pBBR∅	4.74E+08	3.73E+08	to	5.75E+08	****	<0.0001
	Δmtal3 pBBR∅	4.53E+08	3.52E+08	to	5.54E+08	****	<0.0001
Δmtal1	*Δmtal2*	−5.40E+07	−1.55E+08	to	4.70E+07	ns	0.7161
	Δmtal3	−2.40E+07	−1.25E+08	to	7.70E+07	ns	0.9978
	Δmtal1 pBBR-*mtal1*	−3.42E+08	−4.43E+08	to	−2.41E+08	****	<0.0001
	Δmtal2 pBBR-*mtal2*	−3.17E+08	−4.18E+08	to	−2.16E+08	****	<0.0001
	Δmtal3 pBBR-*mtal3*	−3.42E+08	−4.43E+08	to	−2.41E+08	****	<0.0001
	Δmtal1 pBBR∅	−6.96E+07	−1.71E+08	to	3.15E+07	ns	0.3892
	Δmtal2 pBBR∅	9.99E+06	−9.10E+07	to	1.11E+08	ns	>0.9999
	Δmtal3 pBBR∅	−1.08E+07	−1.12E+08	to	9.02E+07	ns	>0.9999

Δ*mtal2*	Δ*mtal3*	3.00E+07	−7.10E+07	to	1.31E+08	ns	0.9891
	Δ*mtal1* pBBR-*mtal1*	−2.88E+08	−3.89E+08	to	−1.87E+08	****	<0.0001
	Δ*mtal2* pBBR-*mtal2*	−2.63E+08	−3.64E+08	to	−1.62E+08	****	<0.0001
	Δ*mtal3* pBBR-*mtal3*	−2.88E+08	−3.89E+08	to	−1.87E+08	****	<0.0001
	Δ*mtal1* pBBR∅	−1.56E+07	−1.17E+08	to	8.55E+07	ns	>0.9999
	Δ*mtal2* pBBR∅	6.40E+07	−3.70E+07	to	1.65E+08	ns	0.5027
	Δ*mtal3* pBBR∅	4.32E+07	−5.78E+07	to	1.44E+08	ns	0.8973
Δ*mtal3*	Δ*mtal1* pBBR-*mtal1*	−3.18E+08	−4.19E+08	to	−2.17E+08	****	<0.0001
	Δ*mtal2* pBBR-*mtal2*	−2.93E+08	−3.94E+08	to	−1.92E+08	****	<0.0001
	Δ*mtal3* pBBR-*mtal3*	−3.18E+08	−4.19E+08	to	−2.17E+08	****	<0.0001
	Δ*mtal1* pBBR∅	−4.56E+07	−1.47E+08	to	5.55E+07	ns	0.8657
	Δ*mtal2* pBBR∅	3.40E+07	−6.70E+07	to	1.35E+08	ns	0.9749
	Δ*mtal3* pBBR∅	1.32E+07	−8.78E+07	to	1.14E+08	ns	>0.9999
Δ*mtal1* pBBR-*mtal1*	Δ*mtal2* pBBR-*mtal2*	2.50E+07	−7.60E+07	to	1.26E+08	ns	0.9971
	Δ*mtal3* pBBR-*mtal3*	0.00E+00	−1.01E+08	to	1.01E+08	ns	>0.9999
	Δ*mtal1* pBBR∅	2.73E+08	1.72E+08	to	3.74E+08	****	<0.0001
	Δ*mtal2* pBBR∅	3.52E+08	2.51E+08	to	4.53E+08	****	<0.0001
	Δ*mtal3* pBBR∅	3.32E+08	2.31E+08	to	4.33E+08	****	<0.0001
Δ*mtal2* pBBR-*mtal2*	Δ*mtal3* pBBR-*mtal3*	−2.50E+07	−1.26E+08	to	7.60E+07	ns	0.9971
	Δ*mtal1* pBBR∅	2.48E+08	1.47E+08	to	3.49E+08	****	<0.0001
	Δ*mtal2* pBBR∅	3.27E+08	2.26E+08	to	4.28E+08	****	<0.0001
	Δ*mtal3* pBBR∅	3.07E+08	2.06E+08	to	4.08E+08	****	<0.0001
Δ*mtal3* pBBR-*mtal3*	Δ*mtal1* pBBR∅	2.73E+08	1.72E+08	to	3.74E+08	****	<0.0001
	Δ*mtal2* pBBR∅	3.52E+08	2.51E+08	to	4.53E+08	****	<0.0001
	Δ*mtal3* pBBR∅	3.32E+08	2.31E+08	to	4.33E+08	****	<0.0001
Δ*mtal1* pBBR∅	Δ*mtal2* pBBR∅	7.96E+07	−2.15E+07	to	1.81E+08	ns	0.2242
	Δ*mtal3* pBBR∅	5.88E+07	−4.22E+07	to	1.60E+08	ns	0.615
Δ*mtal2* pBBR∅	Δ*mtal3* pBBR∅	−2.08E+07	−1.22E+08	to	8.03E+07	ns	0.9993

Abbreviations: SS: sum of squares, DF: degrees of freedom, MS: mean square, Mean Diff.: mean difference.

References

1. Lange, O. et al. Programmable DNA-binding proteins from *Burkholderia* provide a fresh perspective on the TALE-like repeat domain. *Nucleic Acids Res.* **42**, 7436-7449 (2014).
2. Lackner, G. et al. Global distribution and evolution of a toxinogenic *Burkholderia*-*Rhizopus* symbiosis. *Appl. Environ. Microbiol.* **75**, 2982-2986 (2009).
3. Brameier, M., Krings, A. & MacCallum, R. M. NucPred-predicting nuclear localization of proteins. *Bioinformatics (Oxford, England)* **23**, 1159-1160 (2007).
4. Kosugi, S., Hasebe, M., Tomita, M. & Yanagawa, H. Systematic identification of cell cycle-dependent yeast nucleocytoplasmic shuttling proteins by prediction of composite motifs. *Proc. Natl. Acad. Sci. U.S.A.* **106**, 10171-10176 (2009).
